# NS1‐Induced PARP1 Phase Transitions Prolongs Host Transcriptional Pausing to Promote Influenza A Virus Replication

**DOI:** 10.1002/advs.77883

**Published:** 2026-09-27

**Authors:** Shufeng Ma, Changyuan Kang, Xixian Liu, Lichao Yang, Mengyi Li, Yuting Ye, Jianfang Zhang, Yingjun Li, Guanghui Jin, Yang Liu, Xiaohong Chen, Kexin Wang, Zifeng Yang

**Affiliations:** ^1^ State Key Laboratory of Respiratory Disease National Clinical Research Center for Respiratory Disease National Center for Respiratory Medicine Guangzhou Institute of Respiratory Health The First Affiliated Hospital of Guangzhou Medical University Guangzhou China; ^2^ Guangzhou National Laboratory Guangzhou China; ^3^ Guangzhou Key Laboratory for Clinical Rapid Diagnosis and Early Warning of Infectious Diseases KingMed School of Laboratory Medicine Guangzhou Medical University Guangzhou China; ^4^ Engineering Technology Research Center of Intelligent Diagnosis for Infectious Diseases in Guangdong Province Guangzhou China; ^5^ Guangdong Provincial Engineering Research Center for Early Warning and Diagnosis of Respiratory Infectious Diseases Guangzhou China; ^6^ Macao Institute of Systems Engineering Macao University of Science and Technology Macau SAR China

**Keywords:** biology, cell biology, chromatin, chromatin remodeling, influenza A virus, parp1, polymerase, rna polymerase ii

## Abstract

Influenza A virus (IAV) commandeers host transcriptional machinery to replicate, but mechanisms remain elusive. CRISPR screen identifies PARP1 as a proviral factor that binds viral NS1. ATAC‑seq reveals IAV drives PARP1‑dependent chromatin remodeling at promoter‑proximal regions (−100 to +300 bp), linking PARP1 to viral RNA synthesis. Mechanistically, NS1 engages PARP1's helical domain (HD), which alone forms large, irregular condensates with low fluidity, acting as a built‑in rheostat. HD deletion confers high droplet mobility, whereas NS1 binding locks PARP1 into a hyper‑condensed, gel‑like state. This phase transition traps Pol II in prolonged pause by enriching pausing factors (SPT5^‑PLD^, NELFA, NELFE) and excluding release factors (SPT5^‑PRD^, AFF4). The paused environment is hijacked for efficient cap‑snatching and viral RNA synthesis. This proviral activity is independent of PARP1's polymerase activity, revealing a non‑canonical mechanism that complements recent reports of PARP1‑mediated antiviral ADP‑ribosylation. Targeting this phase transition, we identify tectochrysin (TEC) and isoferulic acid (IFA), which restore fluidity, reverse pausing, and suppress IAV in mice and lung organoids without toxicity. Our work establishes that NS1 coopts PARP1 phase separation via the HD rheostat to prolong transcriptional pausing, introducing ‘phase transition correction’ as an antiviral paradigm.

## Graphical abstract

After IAV infection, NS1 induces PARP1 phase transition, reducing molecular mobility in condensates, prolonging host transcriptional pause, aiding RdRp cap snatching, and promoting viral transcription.

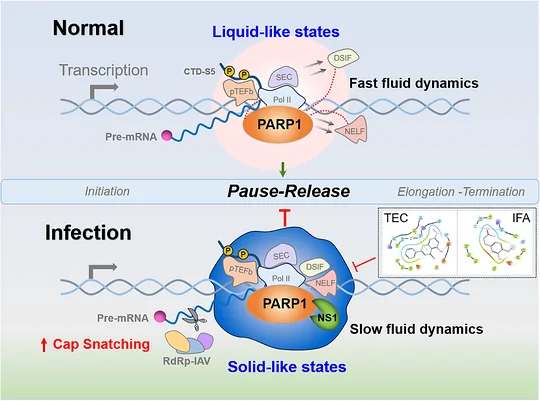

## 1 Introduction

IAV remains a leading cause of severe respiratory illness worldwide, accounting for approximately one billion infections, 3–5 million severe cases, and up to 650 000 deaths annually [1]. The efficacy of current vaccines is increasingly compromised by antigenic drift and shift [2, 3], while antiviral drugs targeting viral proteins face rapid emergence of resistance [4, 5]. Furthermore, post‐pandemic immune alterations—including impaired innate immunity [6] and dysregulated inflammatory responses [7]—have exacerbated disease severity, underscoring the urgent need for host‐directed antiviral strategies that are both effective and resilient to viral mutation.

IAV is a negative‑sense RNA virus whose entire life cycle—including attachment, entry, uncoating, nuclear import of viral ribonucleoproteins (vRNPs), transcription, replication, assembly, and release—depends on host cellular processes, particularly the nuclear transcription apparatus [8]. Upon entry into the nucleus, IAV initiates transcription via a cap‑snatching mechanism. Specifically, the viral RNA‑dependent RNA polymerase (RdRp; PA, PB1, PB2) engages the Ser5‑phosphorylated C‑terminal domain (CTD‐S5P) of host RNA polymerase II (Pol II) to snatch 5’ caps from nascent pre‑mRNAs, which serve as primers for viral mRNA synthesis [9, 10]. However, traditional antiviral strategies that directly inhibit the viral RdRp are hampered by rapid drug resistance resulting from antigenic drift and shift, while current conventional host‑targeted approaches often risk interfering with essential cellular functions. An ideal host‑directed approach should therefore exploit a vulnerability that is dispensable for normal cell homeostasis yet critical for viral transcription. Transcription pausing—a reversible promoter‐proximal arrest of RNA polymerase II (Pol II)—represents such a vulnerability [11]. During this paused state, IAV performs the aforementioned cap‑snatching, acquiring the 5′ cap from host pre‐mRNAs to prime viral mRNA synthesis while simultaneously degrading host transcripts, including antiviral genes [9, 10, 11]. Consequently, modulating the duration or dynamics of transcriptional pausing could disrupt the timing of cap snatching and suppress viral transcription, without directly inhibiting the enzymatic activity of host polymerases.

Phase separation is a fundamental biophysical process that drives the formation of dynamic, membrane‐less condensates in eukaryotic cells. Unlike canonical subcellular organelles delimited by lipid bilayers, these condensates arise from multivalent interactions among biological macromolecules and are intrinsically linked to the regulation of host transcription [12]. Emerging evidence indicates that phase separation of transcription regulators governs the transition between paused and elongating states [13, 14]. The fluidity and composition of these condensates determine whether Pol II remains paused or proceeds into productive elongation. Notably, the viral non‐structural protein NS1 has emerged as a key regulator bridging IAV replication and host transcriptional machinery [15, 16], yet its precise role in modulating host pause‐release dynamics remains incompletely understood.

Poly (ADP)‐ribose polymerase 1 (PARP1) is an abundant nuclear protein well‐known for its role in DNA repair, yet it also participates in DNA replication, transcription, and co‐transcriptional splicing in the absence of DNA damage [17]. Earlier reports have indicated that PARP1's enzymatic activity is critical for understanding its pro‑ versus anti‑IAV functions. Specifically, PARP1 has been identified as an interacting partner of IAV polymerases [18], modulating their activity [19, 20] and controlling HA‐induced degradation of the type I interferon receptor (IFNAR) [21], which favors viral survival in vivo. Additionally, the use of PARP1 inhibitors has shown antiviral phenotypes in mice [22]. Paradoxically, a recent study also demonstrated that IAV triggers PARP1‑mediated ADP‑ribosylation that restricts viral replication in cells, with NS1 counteracting this antiviral effect; notably, PARP1 knockout accelerated viral growth [23]. These discrepancies suggest that PARP1's enzymatic activity may exert context‑dependent pro‑ or antiviral effects, while its non‑catalytic roles in IAV infection remain poorly understood.

In this study, using a CRISPR screen and proteomics, we identified PARP1 as a critical proviral host factor that directly binds NS1. Unlike its well‑characterized role in DNA repair and inflammation via the catalytic ART domain responsible for poly(ADP‑ribose) polymerase activity [24, 25], we uncovered a non‑canonical function of PARP1—phase separation—mediated by HD. We show that NS1 binds the HD of PARP1 to induce aberrant phase separation, reducing droplet fluidity. This leads to sustained sequestration of pausing factors (SPT5^‑PLD^, NELFA, NELFE) and exclusion of pause‑release factors (SPT5^‑PRD^, AFF4), prolonging host transcriptional pausing and enhancing IAV RNA synthesis. Targeting this pathological phase transition, we identified TEC and IFA, which restore PARP1 droplet dynamics, reverse NS1‑induced pausing, and potently inhibit IAV in mice and human airway/alveolar organoids. Collectively, our study reveals a non‑catalytic, proviral function of PARP1 hijacked by NS1 via phase separation, and provides proof‑of‑concept that correcting virus‑induced pathological phase transitions represents a broad‑spectrum antiviral strategy.

## 2 Results

### 2.1 Identification of PARP1 Enhancing Influenza Viral Infection

**FIGURE 1:**
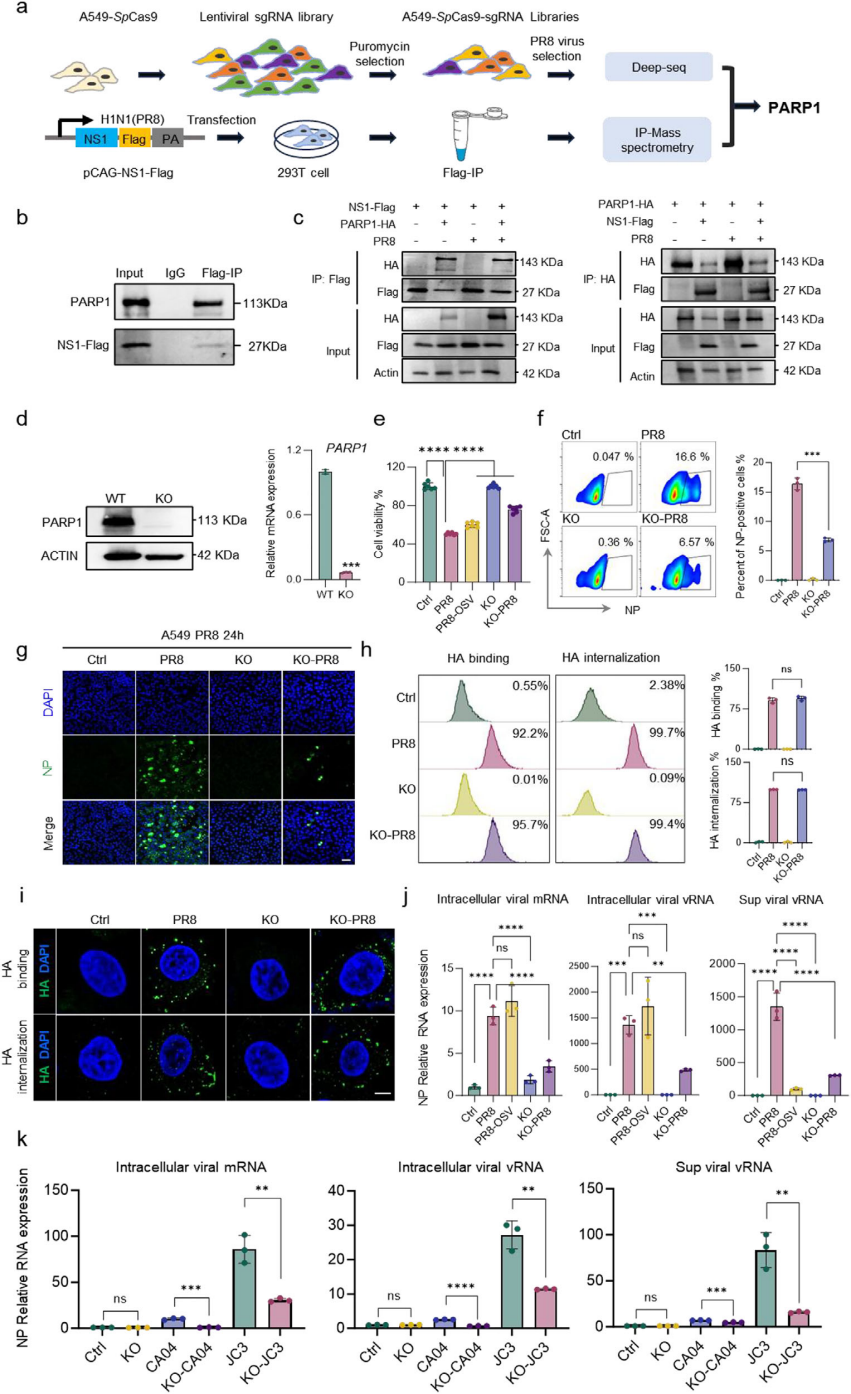
Identification of PARP1 enhancing influenza viral infection. (a) Schematic of the CRISPR‐based screening workflow to identify candidate host proteins involved in viral infection. (b, c) Co‐IP assays demonstrating the interaction between influenza virus NS1 protein and PARP1. HEK293T cells transfected with the indicated plasmids were harvested at 48 h post‐transfection for immunoprecipitation using anti‐Flag or anti‐HA antibodies, followed by western blotting. (b) Interaction between Flag‐NS1 and endogenous PARP1. (c) Interaction between HA‐PARP1 and Flag‐NS1. (d) Generation of a PARP1‐knockout (KO) A549 cell line using CRISPR–Cas9, validated by Western blot and qPCR. (e) Cell viability measured by MTT assay in PARP1‐KO and wild‐type (WT) cells at 24 h post‐infection with PR8 virus. (f, g) Analysis of viral infection efficiency by flow cytometry (f) and immunofluorescence staining (g) in the indicated cell lines. Scale bar, 50 µm. (h) Flow cytometry histograms showing H1N1 HA surface binding (left) and internalization (right) in WT and PARP1‐KO cells. (i) Representative immunofluorescence images of H1N1 HA binding (4°C) and internalization (37°C). Nuclei were stained with DAPI (blue) and HA was detected with specific antibody (green). Scale bar, 5 µm (j, k) RT PCR quantification of viral RNA levels in cell culture supernatant (vRNA in supernatant) and intracellular viral RNA (vRNA and mRNA) at 24 h post‐infection with PR8 or CA04, or JC3. Experimental groups: Ctrl (WT A549, uninfected), PR8/CA04/JC3 (WT A549, infected), KO (PARP1‐KO, uninfected), KO‐PR8/CA04/JC3 (PARP1‐KO, infected). PR8: A/Puerto Rico/8/1934. CA04: A/California/04/2009. JC3: GZ/JC3/2024. Data are presented as mean ± SD from three independent biological replicates. Statistical significance was determined by one‐way ANOVA: **p < 0.01, ***p < 0.002, ****p < 0.001, ns, not significant.

To identify host factors involved in IAV transcription, we performed a CRISPR screen using an RNA‐binding protein knockout library combined with sgRNA sequencing of infected versus mock‐treated cells, along with NS1 immunoprecipitation mass spectrometry (Figure 1a). Among the candidates, PARP1 emerged as a top hit. Co‑immunoprecipitation (Co‑IP) assays confirmed that ectopically expressed Flag‑tagged NS1 co‑precipitated both endogenous PARP1 and exogenous HA‑tagged PARP1, demonstrating a specific interaction (Figure 1b,c).

To assess the functional role of PARP1 in IAV infection, we generated a PARP1‑knockout (KO) A549 cell line (Figure 1d). Upon PR8 virus infection, PARP1 KO significantly improved cell viability from 60.0% (wild‑type infected) to 75.9% (KO‑PR8) at 24 h post‑infection (hpi) (Figure 1e). Flow cytometry and immunofluorescence analyses revealed that the percentage of infected cells dropped from 16.4% in the PR8 group to 6.87% in the KO‑PR8 group (Figure 1f,g). Dissection of the viral life cycle showed that PARP1 loss did not affect HA surface binding or internalization (Figure 1h,i), but strongly inhibited subsequent intracellular viral transcription and progeny release. Quantification of viral RNA demonstrated that intracellular vRNA and mRNA levels in KO‑PR8 cells were reduced by 64.6% and 63.4%, respectively (Figure 1j). Notably, while the neuraminidase inhibitor oseltamivir (OSV) potently blocked virus release but not intracellular RNA synthesis, PARP1 knockout inhibited both, indicating a distinct antiviral mechanism (Figure 1j). We extended these findings to two additional human H1N1 strains—A/California/04/2009 (CA04) and a clinical isolate, GZ/JC3/2024 (JC3)—and consistently observed marked suppression of viral transcription and replication, phenocopying the results obtained with PR8 infection (Figure 1k). Collectively, these data position PARP1 as an early, transcription‑dependent host factor that acts at a post‑entry step of the IAV life cycle.

RNA‑Seq analysis revealed that PR8 infection profoundly altered host transcription in a time‑dependent manner (Figure S1). Differentially expressed genes at 3 hpi (transcription onset) were enriched in innate immune pathways; at 6 hpi (transition phase) additional transcriptional changes emerged; and at 12 hpi (replication phase) adaptive immune responses and membrane remodeling genes were induced (Figure S1e). Gene clustering identified a cluster (cluster 2) associated with ADP‑ribosylation and transcriptional repressors (Figure S1f,g). PARP1 levels increased modestly between 3 and 6 hpi, coinciding with the rise in IAV transcription (Figure S2a). PARP1 knockout enhanced cell survival and suppressed viral transcription/replication at multiple time points (Figure S2b–d). Thus, targeting PARP1 to inhibit IAV transcription represents an early antiviral intervention strategy.

### 2.2 IAV Infection Drives PARP1‐Dependent Chromatin Remodeling at Promoter‐Proximal Regions

**FIGURE 2:**
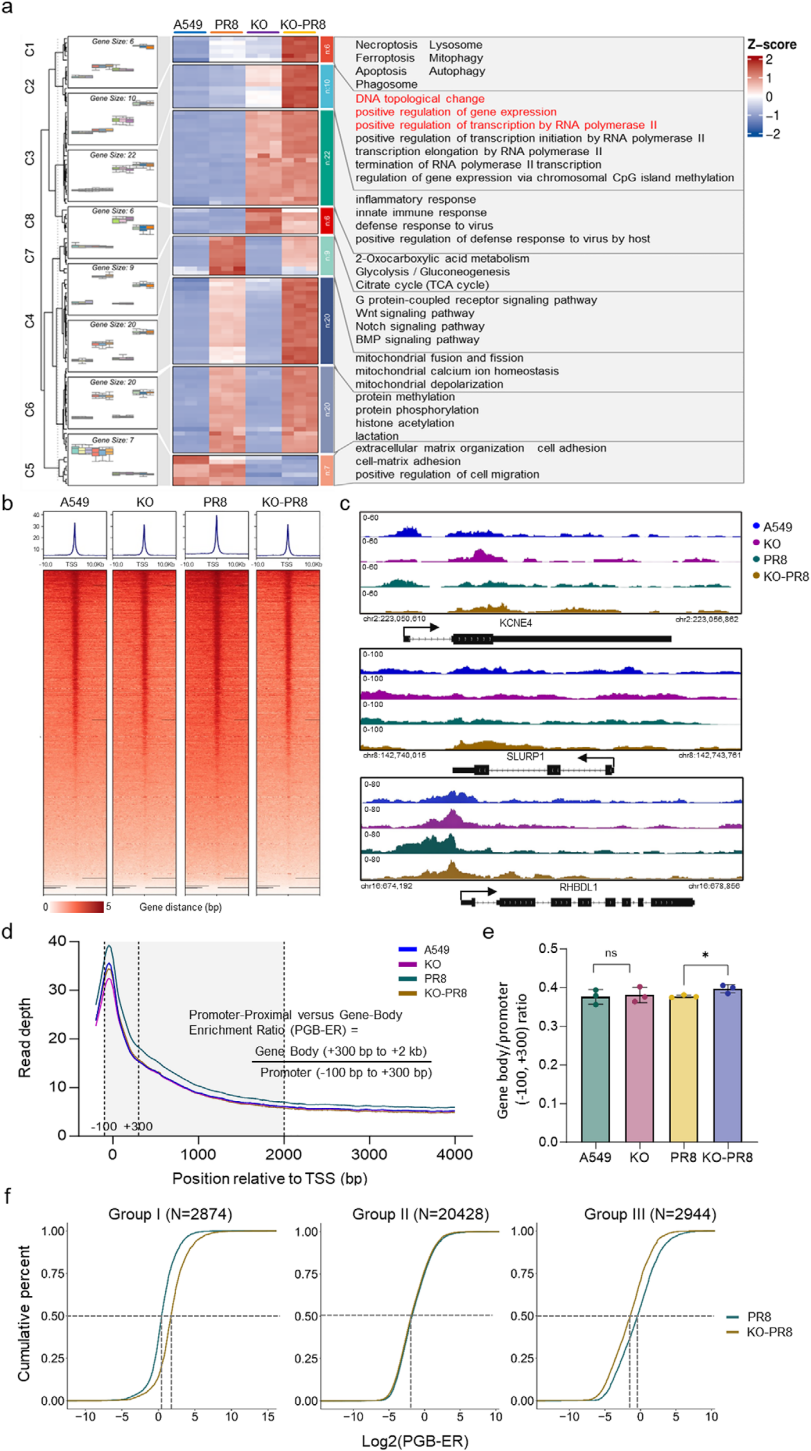
IAV infection drives PARP1‐dependent chromatin remodeling at promoter‐proximal regions. (a) Heatmap derived from RNA‑seq data showing unsupervised hierarchical clustering of genes involved in eight major biological processes. Red indicates up‑regulation, blue indicates down‑regulation. (b) Genome‑wide ATAC‑seq peak matrix. Each column represents a merged consensus peak; color intensity reflects chromatin accessibility (red, high; white, low). (c) Genome‑browser tracks displaying chromatin accessibility at representative loci (KCNE4, SLURP1, RHBDL1). (d) Meta‑profile of average chromatin accessibility across gene bodies and flanking regions (−200 bp to +4000 bp relative to the transcription start site, TSS). (e) Ratio of gene‑body accessibility to promoter accessibility under each condition. (f) ECDF plots comparing the PGB‑ER for differentially affected gene groups. PGB‑ER was calculated as the mean accessibility in the gene body (+300 bp to +2 kb) divided by that in the promoter‑proximal region (−100 bp to +300 bp) in d. Genes were classified into three groups: Group I (higher PGB‑ER in KO‑PR8 vs PR8; fold change > 1.5, log_2_FC > 0.585), Group II (comparable; |log_2_FC| < 0.585), and Group III (higher PGB‑ER in PR8 vs KO‑PR8; fold change > 1.5, log_2_FC > 0.585). Experimental groups: A549 (wild‑type, uninfected), PR8 (wild‑type + PR8 virus), KO (PARP1‑knockout, uninfected), KO‑PR8 (PARP1‑knockout + PR8 virus). Data are presented as mean ± SD of n  =  3 biological replicates; statistical significance was assessed by one‑way ANOVA: *p < 0.05, ns, not significant.

To elucidate how PARP1 regulates IAV transcription, we performed RNA‑Seq on wild‑type and PARP1‑KO cells with or without PR8 infection. Unsupervised hierarchical clustering showed that PARP1 knockout substantially reshaped the expression profiles of genes involved in eight major biological processes (Figure 2a). Gene ontology analysis linked PARP1 to core transcriptional steps, including RNA polymerase II initiation, elongation, and termination, as well as DNA topological changes and CpG island methylation‑mediated regulation (Figure 2a). These findings suggested that PARP1 might modulate transcription by remodeling chromatin accessibility.

We therefore performed ATAC‑seq to assess genome‑wide chromatin accessibility (Figure 2a and Figure S3). IAV infection remodeled the chromatin accessibility landscape, and this remodeling was markedly attenuated in PARP1‑KO cells (Figure 2b). At specific loci (e.g., KCNE4, SLURP1, RHBDL1), PARP1 depletion reduced infection‑induced accessibility at promoter‑proximal regions (Figure 2d). Meta‑profile analysis across gene bodies and flanking regions (−200 bp to +4000 bp from TSS) showed that infection preferentially increased promoter accessibility in wild‑type cells, an effect that was diminished upon PARP1 knockout (Figure 2d). Quantification of the gene‑body‑to‑promoter accessibility ratio revealed a significantly lower ratio in infected wild‑type cells than in infected PARP1‑KO cells, indicating that PARP1 drives a relative increase in promoter over gene‑body accessibility during infection (Figure 2e). Classification of genes by promoter‑to‑gene‑body enrichment ratio (PGB ER) and empirical cumulative distribution function (ECDF) analysis further confirmed that PARP1 mediates promoter‑biased chromatin opening (Figure 2f). Collectively, these data indicate that PARP1 reprograms chromatin accessibility toward promoter‑proximal regions, offering an indirect mechanistic basis for its proviral function that is independent of viral polymerase activity.

### 2.3 NS1 Induces PARP1 Phase Transition to Promote Transcriptional Pausing

**FIGURE 3:**
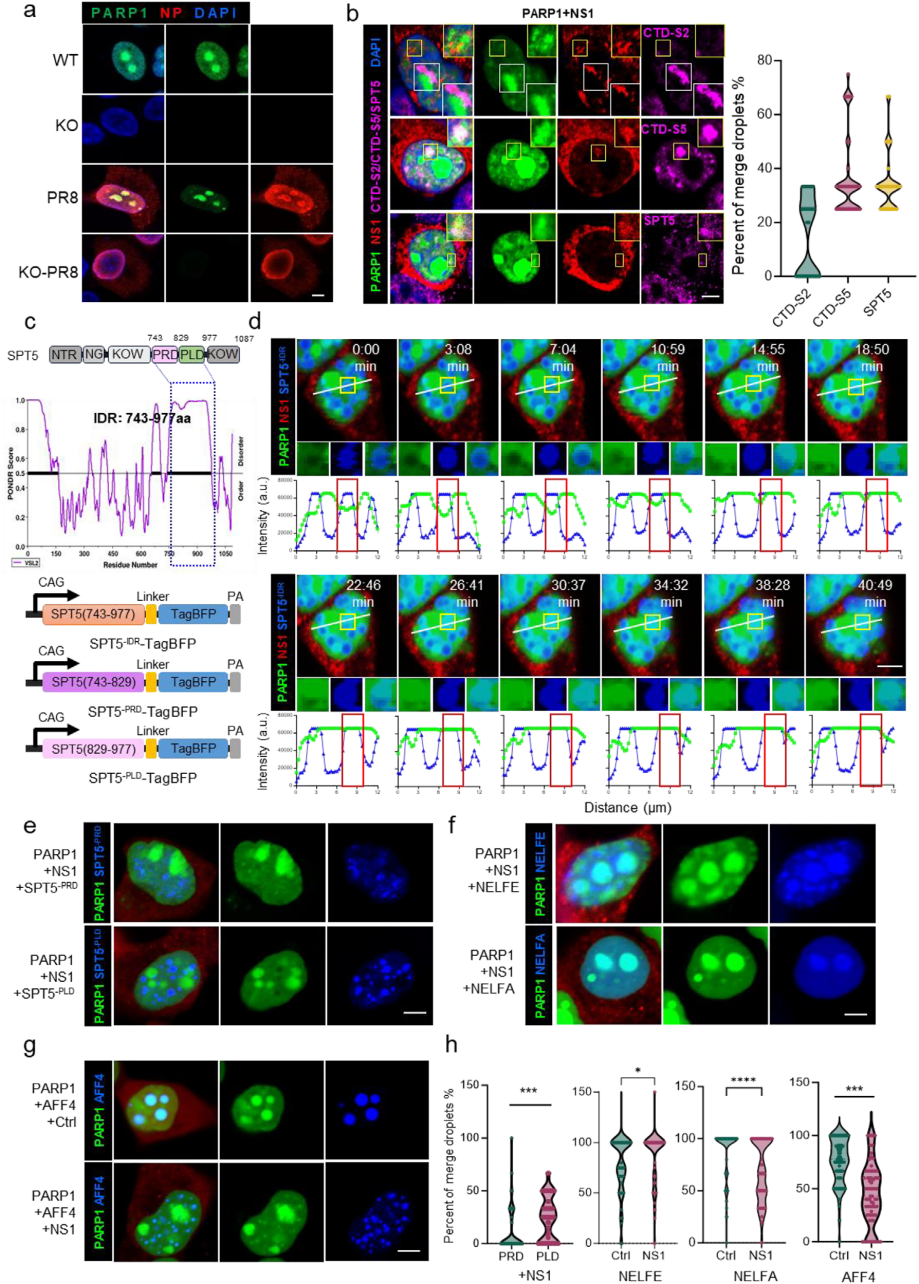
NS1 induces PARP1 phase transition to promote transcriptional pausing. (a) Immunofluorescence staining showing co‐localization of Influenza viral NP protein and endogenous PARP1 in A549 cells 24 h after influenza virus infection. Green: PARP1; red: NP; blue: DAPI (nuclei). (b) HEK293T cells co‐expressing NS1‐mCherry and PARP1‑mNeonGreen were stained for endogenous RNA polymerase II phosphorylated at Ser2 (CTD‑S2P), Ser5 (CTD‑S5P), and the pause‑related factor SPT5 at 48 h post‑transfection. Quantification of co‐localization efficiency is shown on the right, n ≥36 cells. (c) Prediction of intrinsically disordered regions (IDRs) in SPT5 using PONDR‑VSL2. Blue shaded boxes indicate predicted IDR domains. A score of more than 0.5 indicates a disordered region. (d) Live‑cell time‑lapse imaging of PARP1‑mNeonGreen and SPT5^‑CTR^‑TagBFP condensates in HEK293T cells co‑transfected with NS1‑mCherry for 12 h. (e–g) HEK293T cells were co‑transfected with TagBFP‑tagged constructs (SPT5^‑PRD^, SPT5^‑PLD^, or IDRs of NELFA, NELFE, and AFF4) together with NS1‑mCherry (or Ctrl‑mCherry) and PARP1‑mNeonGreen. After 12 h, fluorescence microscopy was used to assess recruitment of the TagBFP‑tagged proteins into PARP1 condensates. (h) The percentage of co‑localized droplets was quantified, n ≥53 cells. Data are presented as mean ± SD from three independent biological replicates. Scale bars, 5 µm. Statistical significance was determined by one‑way ANOVA: *p < 0.05, ***p < 0.002, ****p < 0.001; ns, not significant.

Next, to clarify how PARP1 regulates IAV transcription and whether this relates to the increase in promoter‐proximal accessibility identified in Figure 2, we investigated the potential role of PARP1 in NS1‐mediated viral transcription. Given the established involvement of PARP1 in modulating transcriptional pause‐release and the reliance of IAV on host cap‐snatching for transcription [26, 27], we hypothesized that NS1 may recruit PARP1 to perturb the host pause‐release machinery, thereby creating a permissive transcriptional environment for the virus. To test this hypothesis, we first examined the subcellular localization of PARP1 and viral components. Notably, immunofluorescence staining revealed a clear co‐localization of endogenous PARP1 with the viral NP within the nucleus of infected cells, suggesting a physical association at transcriptionally active sites (Figure 3a). To directly probe the biophysical properties of PARP1, we co‐expressed NS1‐mCherry and PARP1‐mNeonGreen in HEK293T cells. PARP1 readily formed dynamic, phase‐separated condensates. Furthermore, immunostaining of these cells showed that PARP1 condensates co‐localized with markers of transcriptionally paused RNA polymerase II (phosphorylated at Ser5 of the C‐terminal domain, CTD‐S5P) and the pause factor SPT5, but not with the elongation marker CTD‐S2P [28] (Figure 3b).

Previous studies have reported that SPT5 regulates transcriptional pause‐release through phase separation of its intrinsically disordered regions (IDR, SPT5^−IDR^:743‐977aa) [14]. Based on these findings, we hypothesized that PARP1 might interact with SPT5 via phase separation. To address this, we constructed SPT5^−IDR^‐TagBFP plasmid and co‐transfected it with NS1‐mCherry and PARP1‐mNeonGreen plasmids into HEK293T cells (Figure 3c). After 12 h of expression, live‐cell imaging revealed that PARP1 droplets were highly dynamic and significantly enriched SPT5^−IDR^‐TagBFP within 18 min (Figure 3d). Given that SPT5^−IDR^ includes the PRD domain (involved in Pol II elongation) and the PLD domain (involved in maintaining paused Pol II) [29], we separately constructed SPT5^−PRD^‐TagBFP and SPT5^−PLD^‐TagBFP plasmids. After transfecting HEK293T cells, SPT5^−PRD^‐TagBFP formed smaller and irregular foci, while SPT5^−PLD^‐TagBFP formed foci similar in size and shape to those of SPT5^−CTD^ (Figure S4a,b). Upon co‐transfection with NS1‐mCherry, PARP1‐mNeonGreen, and either SPT5^−PRD^‐TagBFP or SPT5^−PLD^‐TagBFP for 12 h, PARP1‐mNeonGreen colocalized with SPT5^−PLD^‐TagBFP at a higher rate (24.0%) than with SPT5^−PRD^‐TagBFP (10.9%) (Figure 3e,h). This suggests that PARP1 preferentially interacts with the SPT5^−PLD^ domain to regulate transcriptional pausing.

To further explore the interactions between PARP1 and key regulators of transcriptional pause‐release via phase separation, we co‐transfected plasmids encoding the IDR domain of NELF (pause), NELFE (pause), and AFF4 (pause‐release) with NS1‐mCherry and PARP1‐mNeonGreen into HEK293T cells. As hypothesized, PARP1 droplets were highly enriched by transcriptional pausing factors (NELFA and NELFE), with colocalization rates exceeding 70% (Figure 3f,h). In contrast, NS1 inhibited the interaction between PARP1 and the pause‐release factor AFF4, reducing the colocalization rate from 75.1% to 45.3% (Figure 3g,h). These findings confirm that NS1 promotes the involvement of PARP1 droplets in transcriptional pausing through phase separation.

Furthermore, to determine whether PARP1 modulates host transcriptional pause‑release dynamics during IAV infection, we performed ChIP‑qPCR to assess RNA Pol II occupancy at the promoter and gene‑body regions of two representative genes, GAPDH and MYC. The results showed that IAV infection significantly increased RNA Pol II enrichment at the gene promoter compared with the gene‑body regions (Figure S4c,d), whereas PARP1 knockout restored these levels to those of uninfected cells. Collectively, these findings suggest that during IAV infection, NS1 may promote PARP1 droplets to interact with pause factors or inhibit interactions with pause‐release factors, shifting host transcription toward a paused state and facilitating viral transcription.

### 2.4 The HD Domain Governs PARP1 Phase Separation and Mediates NS1‐Induced Reduction of Droplet Fluidity

**FIGURE 4:**
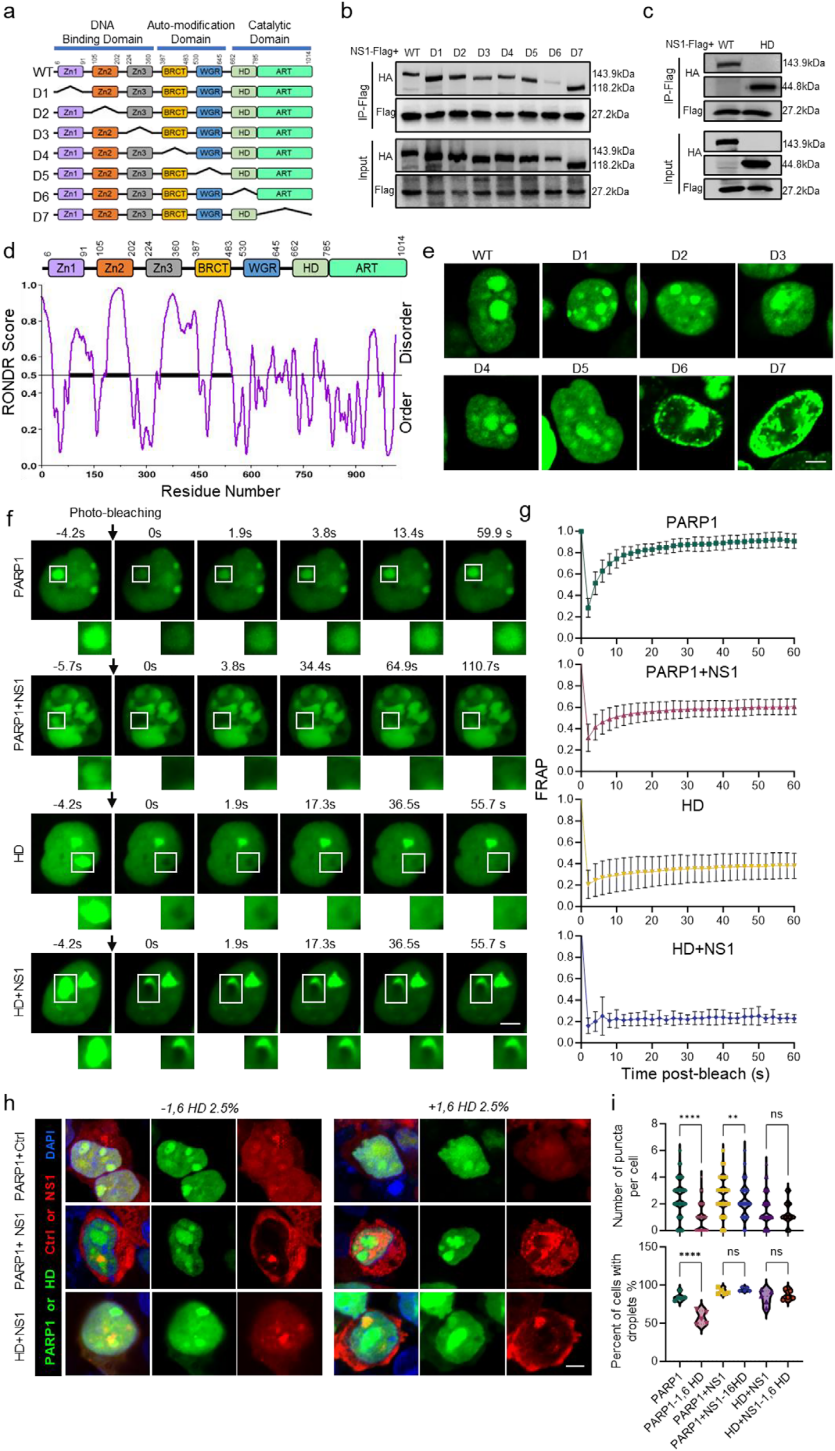
The HD domain governs PARP1 phase separation and mediates NS1‐induced reduction of droplet fluidity. (a) Schematic of PARP1 domain deletion variants. (b) HEK293T cells co‐transfected with NS1‐Flag and PARP1‐HA deletion variants were subjected to Flag‐IP, followed by WB to identify the PARP1 domain interacting with NS1. (c) HEK293T cells co‐transfected with NS1‐Flag and HD‐HA plasmids. After 48 h, Flag‐IP was performed, followed by WB with anti‐HA antibody to identify HD‐NS1 interaction. (d) PONDR‐VSL2 prediction of PARP1 IDRs and Predicted disorder segments with residue ranges and lengths. A score of more than 0.5 indicates a disordered region. (e) HA‐tagged and mNeonGreen‐labeled PARP1 variants, including wild‐type (WT) and mutants (D1, D2, D3, D4, D5, D6, and D7), were constructed. The expression vectors were transfected into 293T cells, and fluorescence was observed to assess the expression and localization of the variants. (f, g) HEK293T cells were transfected with the indicated plasmids. After 48 h, FRAP was performed to evaluate the recovery of fluorescence at bleached foci. Data are plotted as means ± SD (n = 6). (h, i) HEK293T cells co‐transfected with corresponding fluorescent plasmids (PARP1 or HD‐mNeonGreen, Ctrl or NS1‐mCherry) were treated with 2.5% 1,6‐hexanediol for 10 min. Fluorescence microscopy was used to observe condensates (h), and the number of condensates per cell and the proportion of cells with condensates were quantified (i), n ≥116 cells. Mean values were presented with SD, n = 3 biological triplicates. Scale bar, 5µm. One‐way ANOVA test, *p < 0.05, ***p < 0.002, ns, not significant.

To map the domain required for NS1‑PARP1 interaction, we generated HA‑tagged PARP1 deletion mutants lacking ZN1, ZN2, ZN3, BRCT, WGR, HD, or ART domains and co‑expressed them with NS1‑Flag. Pull‑down assays showed that deletion of the HD domain markedly reduced NS1 binding, identifying HD as the critical interaction domain (Figure 4a–c). Direct interaction between NS1 and the isolated HD domain was confirmed by Co‑IP (Figure 4c).

Having established that the HD domain is the key mediator of NS1‐PARP1 interaction, we next examined its role in PARP1 phase separation. We constructed mNeonGreen‐tagged PARP1 deletion mutants (D1‐D7) and transfected them into HEK293T cells (Figure 4d). Notably, the HD‐deleted mutant (D6) exhibited markedly altered phase separation characteristics, forming small, dense foci predominantly localized along the nuclear periphery (Figure 4e). In contrast, the HD domain alone (HD‐mNeonGreen) formed large, irregular foci and displayed poor fluorescence recovery in FRAP assays (Figure 4f,g), suggesting that the HD domain acts as a key switch governing PARP1 phase‑separation fluidity: its deletion promotes high fluidity, whereas its overexpression results in a more condensed, solid‑like state.

To directly assess whether NS1 modulates PARP1 droplet fluidity through the HD domain, we co‐transfected HEK293T cells with NS1‐mCherry or control‐mCherry together with either PARP1‐mNeonGreen or HD‐mNeonGreen, and performed fluorescence recovery after photobleaching (FRAP) experiments. PARP1 droplets recovered from 20% to over 80% within 20 s, reaching up to 98% at 60 s. However, in the presence of NS1, PARP1 droplet recovery was significantly reduced to 50%–60% within 60 s. HD droplets exhibited even lower basal recovery (from 20% to only 30% within 60 s), and this recovery was further diminished upon NS1 co‑expression, with almost no recovery observed (Figure 4f,g). These findings indicate that the HD domain, which directly interacts with NS1, controls the fluidity of PARP1 phase‑separated droplets, and that NS1 binding reduces this fluidity.

Furthermore, we used 1,6‐hexanediol, which is commonly used to dissolve phase‐separated condensates [30], to disrupt condensates in both liquid‐ and solid‐like states (Figure 4h,i). After 10 min of treatment, the percentage of cells with PARP1 droplets decreased from 84% to 57%, while cells co‐transfected with NS1 maintained a high droplet‐positive rate (around 90%). Similarly, cells co‐transfected with NS1 and HD showed no significant change in droplet‐positive rates. The number of PARP1 droplets per cell decreased from 2.3 to 0.9 after 1,6‐hexanediol (1,6 HD) treatment in the absence of NS1, but remained unchanged (PARP1: 2.3 per cell; HD: 1.4 per cell) in the presence of NS1. These results indicate that NS1 stabilizes PARP1 droplets, making them more condensed and resistant to disruption. Collectively, our findings show that NS1 enhances PARP1 droplet condensation, facilitating transcriptional pausing and IAV transcription.

### 2.5 Targeting PARP1 Phase Transition Identifies TEC and IFA as Antiviral Agents

**FIGURE 5:**
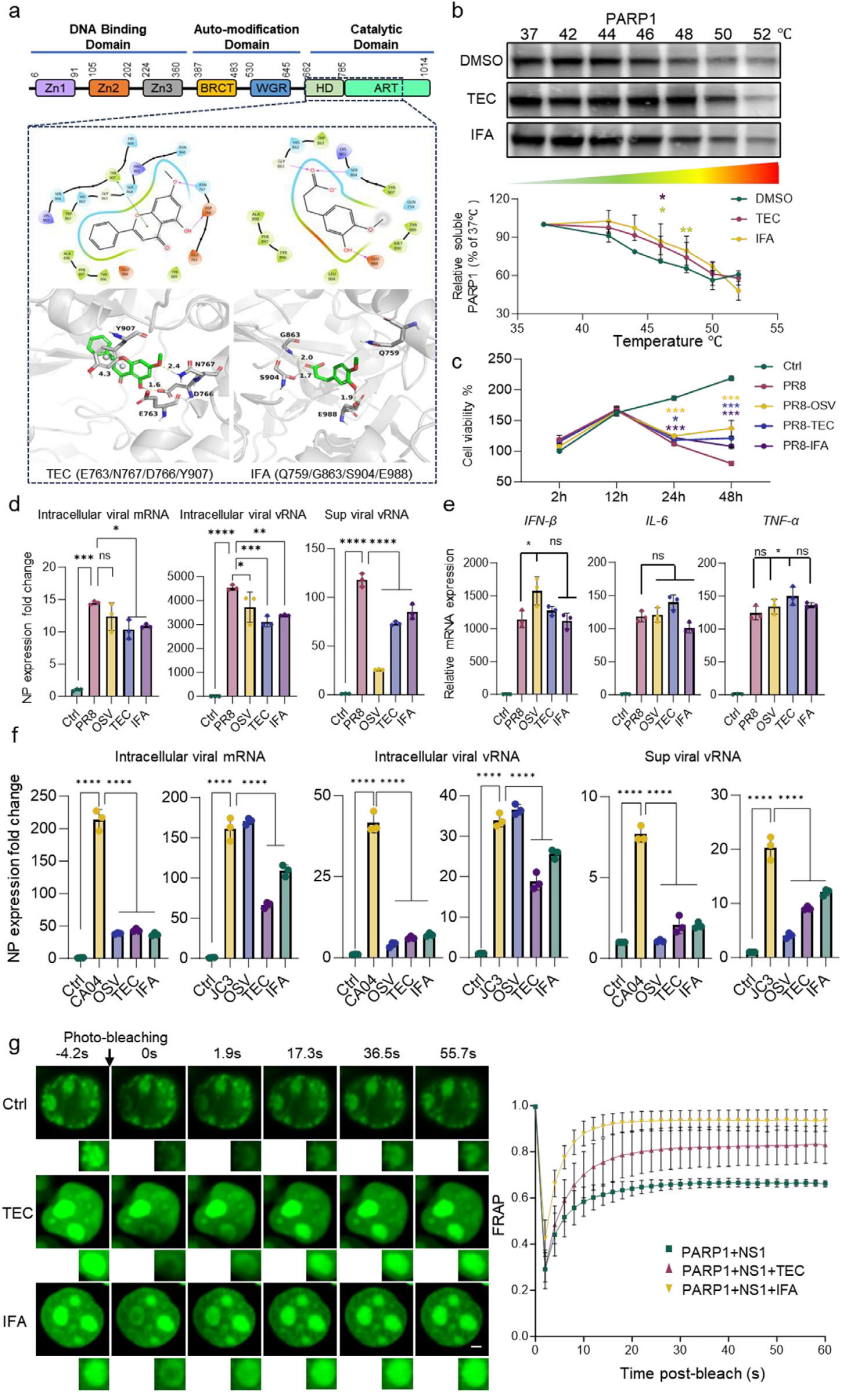
Targeting PARP1 phase transition identifies TEC and IFA as antiviral agents. (a) Molecular docking models depicting the interactions of TEC and IFA with the helical domain (HD) of PARP1. (b) The thermal stability of PARP1 was assessed by CETSA followed by Western blot analysis in lysates treated with DMSO, 1 mM TEC, or 1 mM IFA (Top), and the bands were quantified (bottom). (c) MTT assay of cell viability in IAV‑infected A549 cells treated with TEC or IFA at 2, 12, 24, and 48 h post‐infection. (d, e) qPCR quantification of intracellular viral RNA (d) and mRNA levels of inflammatory cytokines (e) under the indicated treatments. (f) qPCR quantification of viral RNA levels at 24 h post‑infection with PR8, CA04, or JC3, with corresponding drug treatments. (g) FRAP analysis of PARP1‑mNeonGreen condensate mobility in HEK293T cells co‑expressing NS1‑mCherry, treated with or without the indicated compounds for 24 h. Data are plotted as means ± SD (n = 6). Experimental groups in (e, f): Ctrl (A549, mock‐infected), PR8 (A549, IAV‐infected), PR8‑OSV (infected + 10 µM oseltamivir), PR8‑TEC (infected + 2 µg mL^−^^1^ TEC, pretreated 12 h before infection), PR8‑IFA (infected + 10 µg mL^−^^1^ IFA, pretreated 12 h before infection). PR8: MOI = 0.05. Scale bar, 2 µm. Data are mean ± SD (n  =  3). Statistical significance was determined by one‑way ANOVA: *p < 0.05, **p < 0.01, ***p < 0.002, ****p < 0.001, ns, not significant.

Our studies suggest that NS1 may induce PARP1 phase transition to promote IAV transcription, providing a potential example of an NS1–host phase separation interaction mechanism in IAV transcription. These findings prompted us to propose a phase transition–targeted antiviral strategy. Given that the HD domain was critical for NS1–PARP1 phase transition, we performed molecular docking simulations using a traditional Chinese medicine monomer library and the PARP1 HD structure, identifying two HD‐targeting monomers: Tectochrysin (TEC) and Isoferulic acid (IFA) (Figure 5a). Both compounds possess well‑documented anti‑inflammatory and antioxidant properties, with IFA also showing anti‑inflammatory effects in viral infection contexts [31, 32]; however, their direct involvement in IAV infection has been scarcely investigated. We therefore conducted docking analysis to examine their potential interaction with PARP1. Docking results showed that TEC interacts with PARP1 residues E763, N767, D766, and Y907, while IFA interacts with Q759, G863, S904, and E988 (Figure 5a). To assess whether these sites impact PARP1 phase separation and IAV transcription, we introduced mutations (Q759A, E763A, D766A, N767A, G863F, G863A, S904F, S904A, Y907A, E988F, and E988A) using alanine (A) or phenylalanine (F) to simplify or alter the original interactions. FRAP analysis revealed that while all mutants retained phase separation, their condensates exhibited altered characteristics compared to wild‐type PARP1 (Figure S6a). Specifically, mutants Q759A, E763A, D766A, and N767A formed additional faint, small aggregates, and showed reduced fluorescence recovery (approximately 60%) compared to wild‐type PARP1 (80%). In the context of viral transcription, we performed rescue experiments and observed that only wild‑type PARP1 restored viral transcription, whereas the mutants did not (Figure S6b). Furthermore, cellular thermal shift assay (CETSA) demonstrated that both TEC and IFA treatment stabilized PARP1 against thermal denaturation (Figure 5b). Collectively, these results suggest that the residues bound by TEC and IFA are important for PARP1 phase separation, and the compounds may exert their effects by modulating this process.

Next, we evaluated the antiviral effects of TEC and IFA using an A549 PR8 infection model. A549 cells were pre‐treated with TEC or IFA for 12 h before PR8 infection. MTT assays performed at 2, 12, 24, and 48 hpi showed that cell viability was significantly higher in TEC‐ or IFA‐treated groups compared to the PR8 group at 24 and 48 h (Figure 5c). Additionally, qPCR analysis of IAV RNA levels at 24 hpi revealed that TEC and IFA significantly reduced all types of RNA, including both vRNA and mRNA (Figure 5d and Figure S6c,d). Specifically, vRNA levels in the supernatant decreased by 37.9% with TEC and 27.8% with IFA, while intracellular vRNA levels decreased by 31.1% with TEC and 25.4% with IFA. Viral mRNA levels were reduced by 28.6% with TEC and 24.6% with IFA. Additionally, the effects of TEC and IFA on host inflammatory responses, including IFN‐β, IL‐6, and TNF‐α, were evaluated, but no significant differences were observed (Figure 5e). We then extended the analysis to two additional Influenza A virus, CA04 and JC3. Consistent with the results observed for PR8, both TEC and IFA similarly suppressed viral transcription and replication in these strains (Figure 5f). Collectively, these findings indicate that TEC and IFA inhibit influenza A virus infection at the transcriptional phase, and their antiviral effect is not strain‑restricted.

We also assessed the antiviral efficacy of several existing PARP1 inhibitors (Figure S5a), including Olaparib (which primarily targets the ADP‐ribosyl transferase [ART] domain) [33], AG14361 (which also mainly targets the ART domain) [34], Gossypol (targeting the BRCT domain) [35], and NAD^+^ (the substrate) [36], using an A549 cell model infected with the PR8 influenza virus. The results demonstrated that pre‐treatment of A549 cells with these inhibitors failed to significantly suppress viral RNA transcription or replication at 24 hpi and did not significantly modulate the levels of inflammatory cytokines (IFN‐β, IL‐6, TNF‐α) (Figure S5b,c). In contrast, our findings indicated that TEC and IFA differ from conventional PARP1 inhibitors by targeting the phase transition of NS1‐induced PARP1 condensates, thereby significantly inhibiting influenza A virus (IAV) transcription.

Next, to determine whether TEC and IFA could reverse the NS1‐induced PARP1 phase transition, we performed fluorescence recovery after FRAP and 1,6‐hexanediol treatment assays. FRAP analysis revealed that in cells co‐transfected with PARP1‐mNeonGreen and NS1‐mCherry, fluorescence recovery increased from 30% to 60% within 60 s. However, in the presence of TEC, recovery reached 80%, and with IFA, it reached 90% (Figure 5g). Similarly, 1,6‐hexanediol treatment enhanced droplet dissolution: from 92.1% to 91.9% in Ctrl, from 91.4% to 73.5% with TEC, and from 90.3% to 73.5% with IFA (Figure S6e). Notably, the number of intracellular droplets remained unchanged. Collectively, our results demonstrate that TEC and IFA can restore NS1‐induced PARP1 droplet fluidity and inhibit IAV via PARP1 phase transition targeting.

### 2.6 TEC and IFA Ameliorate IAV in Animal Models

**FIGURE 6:**
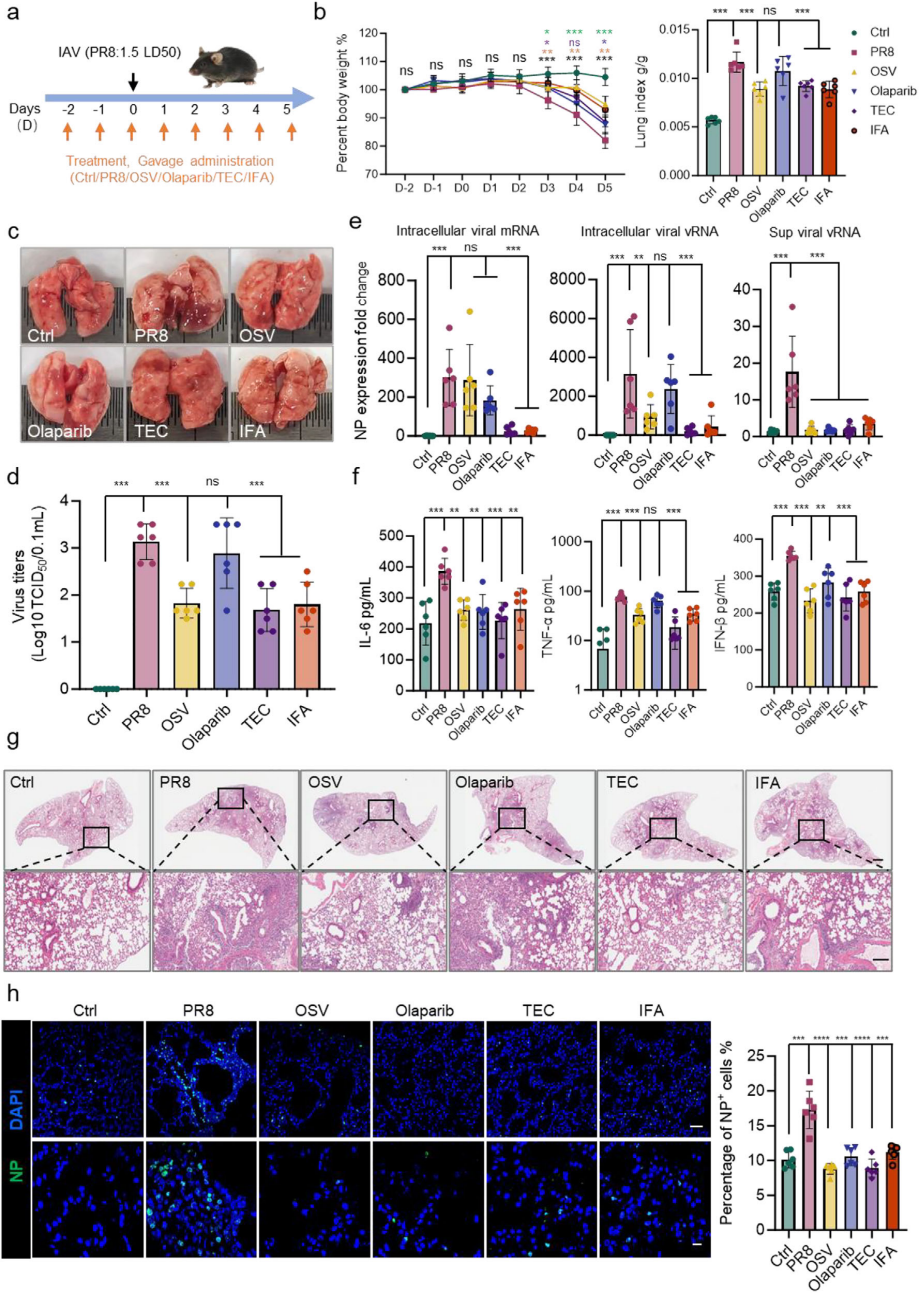
TEC and IFA ameliorate influenza viral infection in animal models. (a) Schematic of the mouse influenza infection model and treatment. Drug dosages: Oseltamivir (OSV), 30 mg/kg; Olaparib, 10 mg/kg; Tectochrysin (TEC), 25 mg/kg; Isoferulic acid (IFA), 20 mg/kg. (b) Body weight (left) and lung index (right) of mice following IAV infection. (c) Lung morphology at 5 days post‐infection (dpi). (d) Viral titers in lung homogenates determined by TCID_50_ assay. (e, f) qPCR analysis of viral RNA (e) and host inflammatory cytokine mRNA (f) levels in lung tissues. (g) H&E staining of lungs. Scale bars: 700 µm (top) and 200 µm (bottom). (h) Immunofluorescence staining and percentage of viral nucleoprotein (NP, red). Nuclei were counterstained with DAPI (blue). Scale bars: 50 µm (top) and 10 µm (bottom). Data are presented as mean ± SD, n = 6 mice; representative images are shown. Statistical significance was determined by one‐way ANOVA: *p < 0.05, **p < 0.01, ***p < 0.002, ****p < 0.001, ns, not significant.

To evaluate the antiviral efficacy of TEC and IFA in vivo, we utilized a mouse model of IAV infection. BALB/c mice were pretreated with TEC (0.5 mg) or IFA (0.4 mg) by oral gavage for two days prior to infection and daily for five days post‐infection (dpi) (Figure 6a). Compared to vehicle‐treated infected controls, mice receiving TEC or IFA showed significantly attenuated body weight loss and reduced lung index (Figure 6b). Lung viral titers were decreased by 1–2 log values in the compound‐treated groups (Figure 6d), accompanied by significant reductions in viral mRNA and vRNA levels (Figure 6e). Cytokine analysis revealed 1‐ to 2‐fold decreases in the levels of IL‑6, TNF‑α, and IFN‑β (Figure 6f). Histopathological examination showed that while control mice exhibited extensive pulmonary hemorrhage and inflammatory infiltration, TEC‐ and IFA‐treated lungs displayed only minor hemorrhage and minimal alveolar thickening (Figure 6g). Immunofluorescence staining for viral NP confirmed the reduction in viral load, with percentage of NP^+^ cells declining from 17.28 in the PR8 group to 8.92 (TEC) and 11.21 (IFA) (Figure 6h). Notably, the PARP1 inhibitor olaparib, included for mechanistic comparison, reduced inflammatory markers and infiltration but did not significantly affect viral transcription (Figure 6e). Collectively, TEC and IFA treatment suppresses IAV replication and mitigates viral pneumonia in a mouse model of infection.

### 2.7 TEC and IFA Reduce Cytotoxic Lymphocyte Infiltration and Preserve Pulmonary Barrier Integrity

**FIGURE 7:**
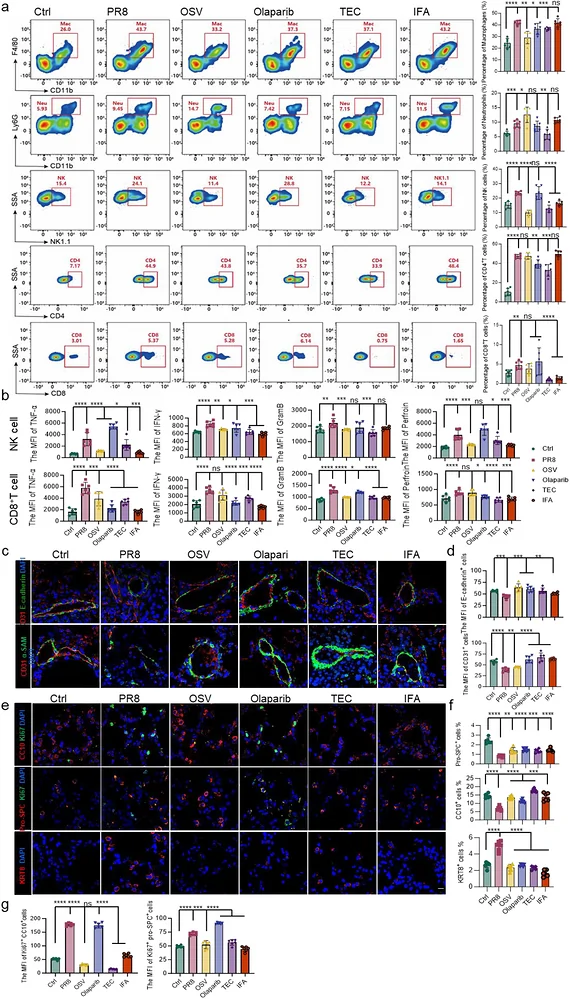
TEC and IFA reduce cytotoxic lymphocyte infiltration and preserve pulmonary barrier integrity. (a) Representative flow cytometry dot plots showing gating strategy for inflammatory infiltrating immune cells in lung tissue, including F4/80^+^ macrophages, Ly6G^+^ neutrophils, NK1.1^+^ NK cells, CD4^+^ T cells, and CD8^+^ T cells (left). Quantitative summary of immune cell frequencies as percentages of total CD45^+^ leukocytes (right). n = 6 mice. (b) Flow cytometric analysis of Granzyme B, Perforin, IFN‑γ, and TNF‑α expression in NK cells and CD8^+^ T cells. n = 6 mice. (c) Immunofluorescence staining of lung tissue showing epithelial integrity (E‐cadherin^+^), endothelial cells (CD31^+^), and vascular structures (α‐SAM^+^). (d) Quantitative analysis of staining intensity and positive area for markers shown in (c). (e) Immunofluorescence staining assessing epithelial injury and repair, showing alveolar stem cell markers (Pro‐SPC^+^ and CC10^+^), proliferative activity (Ki67^+^), and differentiation status (Krt8^+^). (f) Quantification of the proportion of corresponding cells. (g) Quantitative analysis of staining intensity and positive area for the markers shown in (e). n = 6 mice per group. Scale bar, 10µm. One‐way ANOVA test, *p < 0.05, **p < 0.01, ***p < 0.002, ****p < 0.001, ns, not significant.

To evaluate pulmonary inflammatory infiltration and epithelial barrier damage, we performed flow cytometry analysis of immune cells in lung tissues from different treatment groups. The results demonstrated that both TEC and IFA treatment significantly reduced the infiltration of cytotoxic immune cells (Figure 7a). Specifically, the proportion of NK cells decreased from 23.4% in the PR8‐infected control group to 12.63% in the TEC group and 16.42% in the IFA group. Concurrently, CD8^+^ T cell infiltration was reduced from 4.73% to 1.00% (TEC) and 1.35% (IFA). Furthermore, TEC also exhibited a marked inhibitory effect on the infiltration of macrophages, neutrophils, and CD4^+^ T cells. Consistent with the reduced infiltration, TEC and IFA treatment decreased the expression of Granzyme B, Perforin, IFN‑γ, and TNF‑α in both NK cells and CD8^+^ T cells, indicating a dampened cytotoxic response (Figure 7b).

We next assessed lung barrier integrity, focusing on epithelial and endothelial components, as well as epithelial regeneration markers. Consistent with the reduction in immune cell infiltration, TEC and IFA treatment attenuated infection‐induced damage (Figure 7c,d). The mean fluorescence intensity for the epithelial adhesion protein E‐cadherin, which declined from 56.42 to 44.55 post‐PR8 infection, was restored to 57.04 with TEC and 51.12 with IFA. Similarly, the endothelial marker CD31 (MFI decreased from 57.54 to 38.52 after infection) recovered to 67.63 (TEC) and 63.41 (IFA). Staining for α‐smooth muscle actin (α‐SMA) indicated that vascular architecture disruption upon infection was ameliorated by both treatments.

Considering that lung injury activates regenerative pathways, we analyzed the proliferation status of stem and progenitor cell populations (Figure 7e–g). IAV infection triggered a regenerative response, as evidenced by a significant increase in Ki67 expression within bronchiole stem cells (club cells, CC10^+^) and alveolar epithelial type 2 cells (AT2 cells, proSP‐C^+^). The expansion of these proliferating stem cell populations, along with an increase in the repair‐intermediate cell marker KRT8, indicated active tissue repair and substantial epithelial damage. Notably, treatment with TEC or IFA effectively modulated this injury‐induced hyper‐proliferative state.

In summary, TEC and IFA treatment reduces cytotoxic CD8^+^ T cell and NK cell infiltration into infected lungs, attenuates the infection‐triggered hyper‐proliferation of lung stem cells, and promotes the preservation of pulmonary epithelial and endothelial barrier integrity.

### 2.8 TEC and IFA Suppress IAV Infection in Human Airway Organoids

**FIGURE 8:**
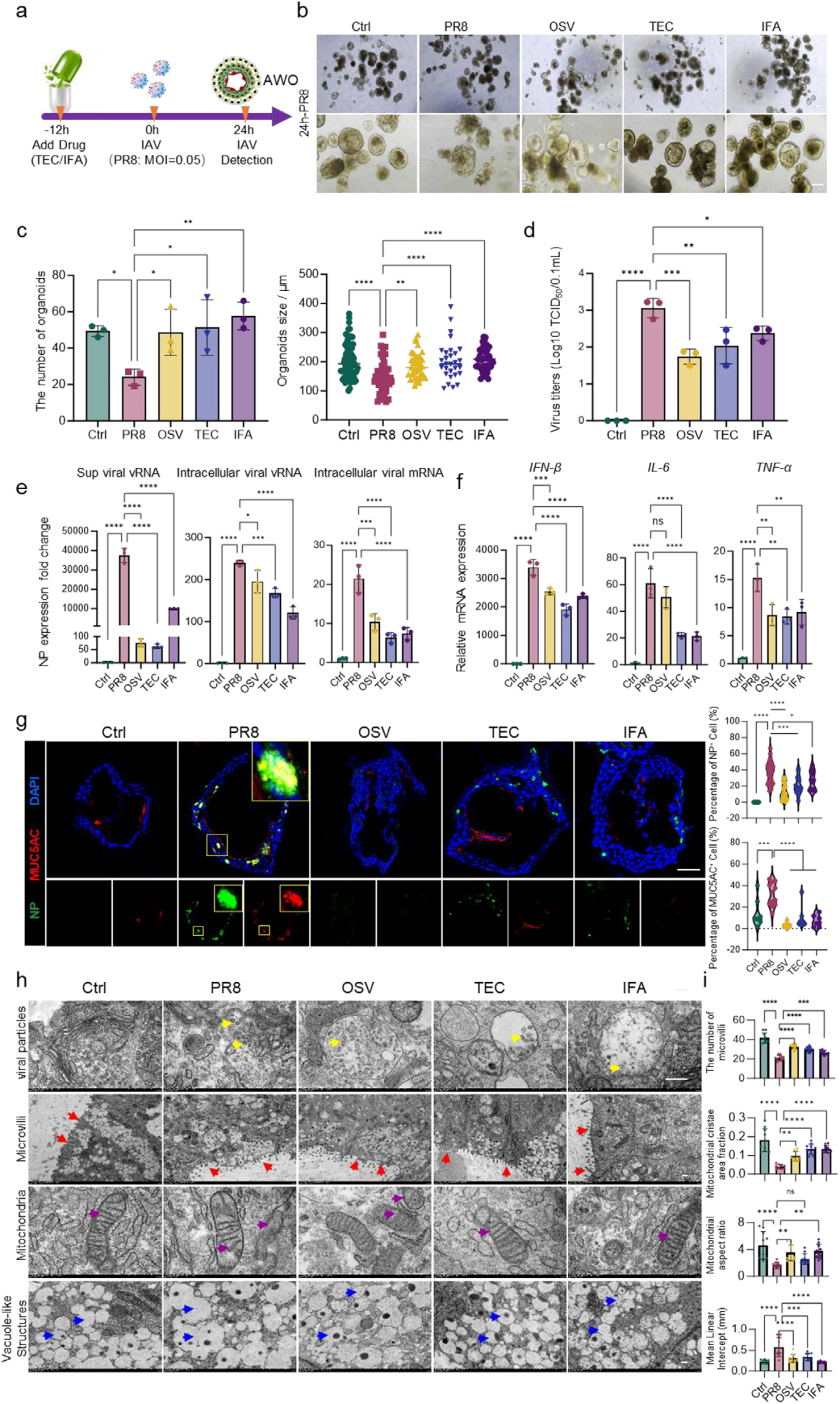
TEC and IFA suppress IAV infection in human airway organoids. (a) Schematic of herbal monomer intervention in airway organoids. (b, c) Airway organoids infected with virus were treated with herbal monomers. Organoid size and number were quantified. Scale bar, 200 µm. (d) TCID50 assay of viral titer in the supernatant post‐treatment. (e, f) qPCR analysis of viral RNA and host inflammatory gene expression in lung tissue. (g) Immunofluorescence staining of viral NP and changes in two types of cells in airway organoids, including goblet cells (MUC5AC) and Clara cells (CC10). Scale bar, 50 µm. (h) TEM analysis of viral particles (yellow arrows), microvilli (red arrows), mitochondria (purple arrows), and vacuole‑like structures (blue arrows). Scale bar, 0.2 µm. (i) Quantification of microvilli number, mitochondria cristae area fraction, mitochondria aspect ratio, and vacuole‑like structure intercept from TEM images. n = 10 fields/group. Mean values were presented with SD, n = 3 biological triplicates. One‐way ANOVA test, *p < 0.05, **p < 0.01, ***p < 0.002, ****p < 0.001, ns, not significant.

To further evaluate the efficacy of TEC and IFA, we utilized human airway organoids (AWO) derived from H9 embryonic stem cells. TEC and IFA were administered 12 h prior to PR8 infection at an MOI of 0.05, and viral replication and host responses were assessed 24 hpi (Figure 8a). IAV infection significantly inhibited organoid growth, as evidenced by a reduction in both size and number (Figure 8b,c). Specifically, PR8 infection reduced organoid counts from 49 to 24 at 4× magnification (a field covering almost the entire 3D gel droplet), whereas OSV, TEC, and IFA reversed this decline, yielding 48, 51, and 57 organoids, respectively. The diameter of organoids also changed, shrinking from 202.6 µm in the control group to 147.9 µm in the PR8‐infected group, but increased to 187.9 µm (OSV), 203.3 µm (TEC), and 208.5 µm (IFA) after treatment. Viral titers decreased by approximately 1 log in treated groups (Figure 8d). QPCR analysis showed significant reductions in viral RNA in both supernatants and cells, along with decreased expression of host inflammatory cytokines (IFN‐β, IL‐6, and TNF‐α) (Figure 8e,f).

Clinically, IAV infection may induce epithelial cell repair, increase goblet cells, and enhance mucus secretion. To investigate these effects, we used immunofluorescence staining to measure the levels of goblet cells and club cells, as well as their co‐expression with viral NP. Upon infection, the proportion of goblet cells increased from 13.8% to 31.3% (Figure 8g), while club cells remained at approximately 30% (Figure S7c,d). However, following treatment with OSV, TEC, and IFA, goblet cell proportions decreased to 3.0%, 10.0%, and 7.0%, respectively, while club cell proportions increased to 45.6%, 46.4%, and 55.5%. The reduction in goblet cells likely limits mucus secretion, while the increase in club cells, acting as stem cells, may enhance epithelial regeneration and repair. Consistent with these observations, NP positivity rates were significantly reduced by drug treatment: from 37.1% in the PR8 group to 13.0% for OSV, 21.08% for TEC, and 27.0% for IFA. Specifically, NP positivity rates in goblet cells dropped from 49.0% in the PR8 group to 9.8% for OSV, 26.8% for TEC, and 7.4% for IFA, with the most significant reduction observed in goblet cells. In club cells, NP positivity rates decreased from 55.8% in the PR8 group to 24.4% for OSV, 40.1% for TEC, and 51.0% for IFA (Figure S7d). Altogether, in the AWO infection model, we observed goblet cell proliferation and mucus secretion, mirroring clinical findings, while treatment with TEC and IFA inhibited mucus secretion and promoted progenitor cell proliferation, potentially contributing to epithelial repair.

Furthermore, transmission electron microscopy (TEM) revealed that PR8 infection led to ciliary disruption, mitochondrial swelling with reduced cristae, increased secretions, and accumulation of viral particles (Figure 8h,i). In contrast, treatment with TEC and IFA restored ciliary structure and mitochondrial integrity, reduced viral load, and normalized cellular ultrastructure. Specifically, treated cells exhibited increased cilia number and alignment, restored mitochondrial morphology and cristae structure, decreased secretions, and reduced intracellular viral particles. Collectively, these findings confirm the antiviral efficacy of TEC and IFA in human airway organoids.

### 2.9 TEC and IFA Inhibit IAV Replication in Human Alveolar Organoids

**FIGURE 9:**
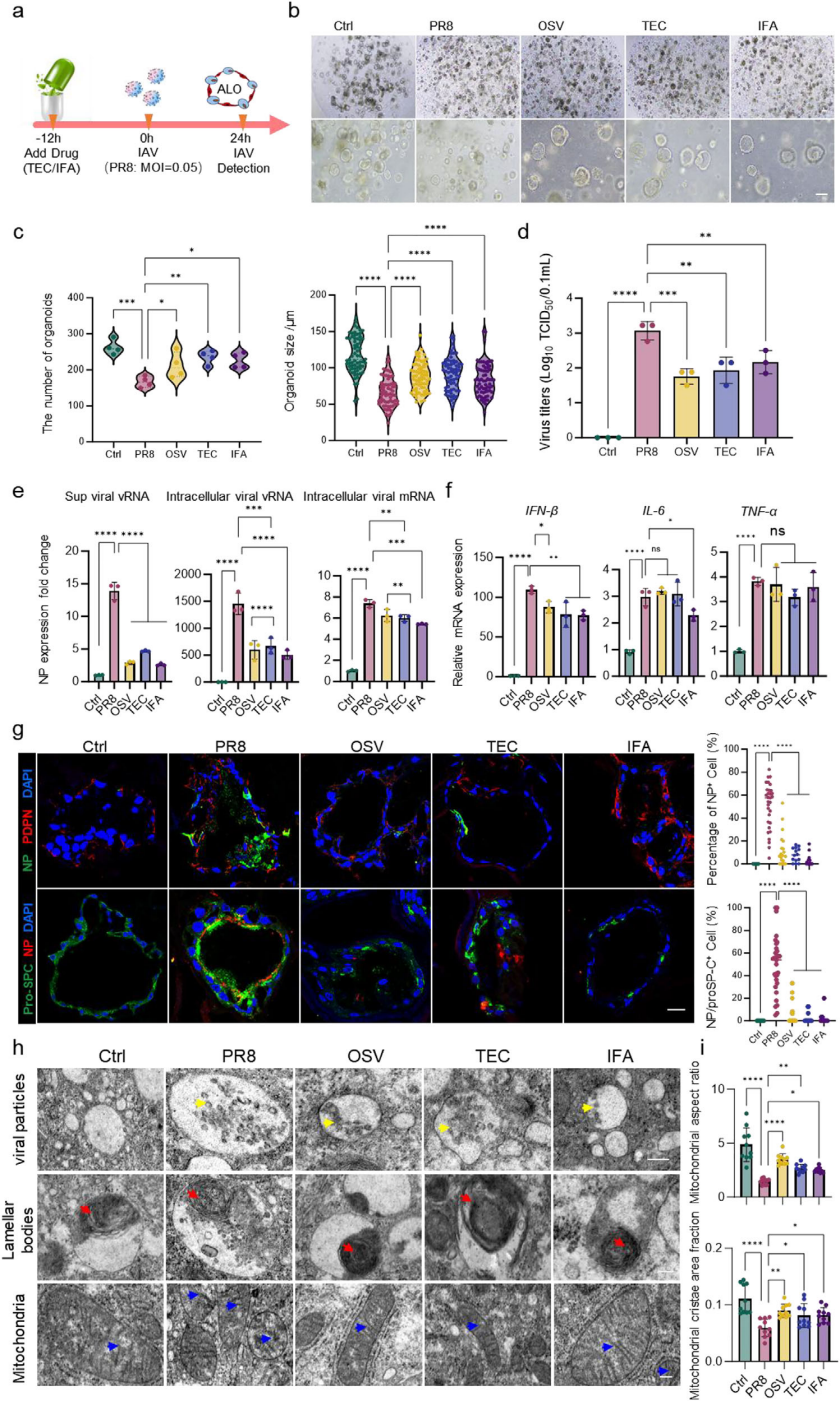
TEC and IFA inhibit IAV replication in human alveolar organoids. (a) Schematic of drug treatment and IAV infection model. (b, c) Morphology, quantity, and size of alveolar organoids. Scale bar, 200µm. (d) TCID50 assay for viral titer. (e, f) qPCR analysis of viral RNA and host inflammatory gene expression (IFN‐β, IL‐6, and TNF‐a) in alveolar organoids. (g) Immunofluorescence staining of viral NP and changes in two types of cells in alveolar organoids, including AT2 cells (proSP‐C) and AT1 cells (PDPN). Scale bar, 50 µm. (h) TEM analysis of viral particles (yellow arrows), lamellar bodies (red arrows), and mitochondria (blue arrows). Scale bar, 0.2 µm. (i) Quantification of mitochondria cristae area fraction and aspect ratio from TEM images. n = 10 fields/group. Mean values were presented with SD, n = 3 biological triplicates. One‐way ANOVA test, *p < 0.05, **p < 0.01, ***p < 0.002, ****p < 0.001, ns, not significant.

To further evaluate the antiviral efficacy of TEC and IFA, we conducted experiments on alveolar organoids (ALO) using the same protocol as with AWO. Specifically, TEC and IFA were applied 12 h prior to PR8 infection, and assessments were performed 24 hpi (Figure 9a). Consistent with our previous findings, TEC and IFA significantly restored PR8‐induced suppression of organoid growth, including both number and size (Figure 9b,c). At 4X magnification, the number of alveolar organoids decreased from 262 in the control group to 166 in the PR8‐infected group, but was restored to 212 (OSV), 235 (TEC), and 225 (IFA) after treatment. The diameter of alveolar organoids also changed, shrinking from 117.5 µm in the control group to 66.5 µm in the PR8‐infected group, but increased to 87.9 µm (OSV), 92.2 µm (TEC), and 83.7 µm (IFA) after treatment. Viral titers decreased by approximately 1 log in treated groups (Figure 9d). The levels of viral RNA (vRNA and mRNA) were significantly reduced in the drug‐treated groups, as determined by QPCR analysis (Figure 9e). Additionally, the expression of inflammatory cytokines decreased slightly, with only IFN‐β showing a significant reduction (Figure 9f).

Next, we used immunofluorescence staining to examine the changes in alveolar epithelial cells (AT1 and AT2) and their viral NP positivity rates (Figure 9g and Figure S8). The overall NP positivity rate decreased significantly from 50.8% in the PR8‐infected group to 12.0% (OSV), 7.9% (TEC), and 3.1% (IFA) after treatment. Specifically, the NP positivity rate in AT2 cells decreased more significantly, from 49.5% to 6.11% (OSV), 2.4% (TEC), and 1.9% (IFA). In contrast, in AT1 cells, the NP positivity rate changed from 18.0% to 29.9% (OSV), 2.6% (TEC), and 7.2% (IFA), showing a downward trend in the latter two. These results suggest that the drugs may be more effective in reducing NP positivity in the highly infected proSP‐C^+^ AT2 cells, although further evidence is needed to confirm this observation. We also quantified the proportions of AT1 and AT2 cells and found opposing trends. The proportion of proSP‐C^+^ AT2 cells decreased in both the PR8‐infected and treated groups (62.3% for non‐infected Ctrl, 52.5% for PR8, 40.9% for TEC, and 46.2% for IFA). In contrast, the proportion of PDPN^+^ AT1 cells increased from 28.7% (Ctrl) to 56.38% after PR8 infection, but decreased significantly after treatment to 31.8% (OSV), 22.5% (TEC), and 34.6% (IFA). These findings indicate that PR8 infection alters the proportion of AT2 and AT1 cells and the types of cells infected.

Finally, TEM analysis revealed that PR8 infection led to the accumulation of viral particles in the lamellar bodies of AT2 cells. In cells treated with TEC and IFA, this accumulation was significantly reduced, indicating the antiviral effects of these compounds (Figure 9h). Moreover, PR8 infection caused mitochondrial swelling and disruption of cristae, which are hallmarks of viral‐induced cellular stress and damage. Notably, these pathological changes were mitigated by treatment with TEC and IFA (Figure 9h,i). Collectively, these findings confirm the antiviral efficacy of TEC and IFA in human alveolar organoids and highlight their potential as therapeutic agents against IAV infection.

## 3 Discussion

In this study, we identified PARP1 as a critical host factor that promotes IAV transcription. Mechanistically, the viral NS1 protein binds the HD of PARP1, induces aberrant phase separation, and reduces droplet fluidity. This results in sustained sequestration of transcriptional pausing factors (SPT5^‑PLD^, NELFA, NELFE) and exclusion of pause‑release factors (SPT5^‑PRD^, AFF4), thereby prolonging host transcriptional pausing and creating a permissive environment for IAV cap‑snatching and viral RNA synthesis. Targeting this pathological phase transition, we identified TEC and IFA, which restore PARP1 droplet dynamics, reverse NS1‑induced pausing, and potently suppress IAV replication in mice and human airway/alveolar organoids. Our work reveals a non‑catalytic proviral PARP1 function and establishes “phase transition correction” as a host‑directed strategy that modulates host transcription, not polymerase inhibition.

### 3.1 PARP1: A Newly Identified Proviral Function via Phase Separation

Previous studies have reported distinct roles for PARP1 during IAV infection, including interaction with the viral polymerase [18, 19, 20] and antiviral ADP‑ribosylation activity [23]. Our study now provides a unifying perspective by revealing a previously unrecognized, non‑catalytic function of PARP1. We demonstrate that NS1 binds the HD domain to induce aberrant phase separation that prolongs host transcriptional pausing and promotes viral transcription—a proviral role independent of the canonical ART domain. Thus, PARP1 can exert both antiviral (via enzymatic activity) and proviral (via HD‑mediated phase separation) functions, depending on which domain is engaged. These findings reconcile previous observations and establish PARP1 as a multi‑faceted host factor.

### 3.2 Phase Separation as a Nexus Between Viral Transcription and Host Pausing Machinery

Phase separation has emerged as a fundamental principle underlying transcriptional regulation [37] and viral infection [38]. Specifically, IAV ribonucleoproteins form liquid organelles near endoplasmic reticulum exit sites, relying on ER‐Golgi vesicular cycling [39], and some studies explored the physical and thermodynamic properties of liquid condensates formed during influenza A virus infection [40]. However, the role of phase separation in virus‐host interactions remains underexplored and is not yet fully understood. Our study reveals that IAV NS1 manipulates PARP1 phase separation to prolong transcriptional pausing. NS1 reduces PARP1 droplet fluidity, leading to persistent association with pausing factors (SPT5^‑PLD^, NELFA, NELFE) and impaired recruitment of pause‑release factors (SPT5^‑PRD^, AFF4). This pathological shift locks host Pol II in a prolonged paused state, which is exploited by IAV for efficient cap‑snatching. Notably, viral NP co‑localizes with PARP1 in the nucleus, suggesting that PARP1 condensates may serve as spatial hubs that concentrate viral transcription components. Our findings thus establish a physical and functional link between IAV replication and host transcriptional pausing via phase separation.

### 3.3 The HD Domain: A Non‑Canonical Regulator of PARP1 Phase Separation

Most studies on PARP1 have focused on its C‑terminal ART domain, which catalyzes poly(ADP‑ribosyl)ation [17, 41, 42] and is targeted by clinically used inhibitors such as olaparib [43, 44]. In contrast, the role of the HD domain in phase separation has been largely unexplored. We found that the HD domain alone forms large, irregular condensates with severely reduced fluidity, and that NS1 binding nearly abolishes HD droplet dynamics (Figure 4f). Deletion of the HD domain (D6 mutant) resulted in small, dense nuclear periphery foci with distinct properties (Figure 4e). These observations suggest that the HD domain acts as a key determinant of PARP1's phase separation behavior. Importantly, molecular docking and mutational analysis identified residues within the HD domain (e.g., Q759, E763, D766, N767, G863, S904, Y907, E988) that are critical for phase separation and are targeted by TEC and IFA (Figure S6a,b). This domain‑specific targeting avoids interfering with the essential catalytic functions of PARP1 in uninfected cells, offering a precision antiviral strategy. Additionally, the D7 mutant (ART deletion) also exhibited altered PARP1 foci size and distribution (Figure 4e), hinting at an additional ART‑dependent layer of phase‑separation regulation that warrants further exploration.

### 3.4 Targeting Phase Transition Versus Enzymatic Activity: A New Therapeutic Paradigm

Two PARP1‑targeting antiviral strategies are identified. Conventional ART‑domain inhibitors (olaparib, AG14361) suppress inflammatory cytokines and immune infiltration but fail to inhibit viral transcription/replication (Figures 6e,h, and 7b). In contrast, TEC and IFA—representing a novel non‑enzymatic phase‑transition strategy—bind the HD domain, reverse NS1‑induced PARP1 condensation, restore droplet fluidity, and inhibit viral replication without affecting cell viability. This distinction is therapeutically relevant: ART inhibitors carry on‑target DNA‑repair toxicity, whereas phase‑transition correction may spare PARP1's housekeeping functions. In vivo, TEC and IFA outperformed olaparib, reducing lung pathology, viral load, and inflammatory cytokines with no obvious adverse effects. Moreover, they reduced infiltration and activity of CD8^+^ T and NK cells, and lowered effector cytokine production in infected lungs. These results suggest that their antiviral efficacy likely stems from both direct replication inhibition and immunomodulation, providing new clues for future mechanistic exploration.

### 3.5 Limitations of the Study

Several questions remain to be addressed in future studies. First, while we found that PARP1 knockout affects chromatin accessibility and transcriptional pausing, the upstream mechanism by which PARP1 drives chromatin remodeling remains elusive and is rarely reported, meriting future exploration. Second, we observed that both HD‑deleted (D6) and ART‑deleted (D7) mutants form similar dense foci predominantly localized at the nuclear periphery, suggesting that PARP1 phase separation is regulated by both the HD and ART domains—the former in a catalytic‑activity‑independent manner (which is the focus of the present study) and the latter in an activity‑dependent manner. How ART‑mediated phase separation contributes to viral infection, and whether it intersects with HD‑mediated regulation, remain interesting questions for future exploration. Third, our pharmacological evaluations across three platforms—cell lines, organoids, and mouse models—revealed that the in vivo efficacy of TEC and IFA was markedly superior to that observed in vitro. Moreover, we found that TEC and IFA modulated the cytotoxic activity of CD8^+^ T cells and NK cells, indirectly preserving lung epithelial barrier integrity and limiting infection spread. These findings highlight the importance of multi‑platform drug evaluation and suggest the possibility of additional target engagement beyond PARP1, which merits further investigation.

## 4 Conclusion

In summary, we have uncovered a previously unrecognized mechanism by which IAV NS1 hijacks PARP1 phase separation to prolong host transcriptional pausing and promote viral replication. We have identified TEC and IFA as first‑in‑class compounds that correct this pathological phase transition by targeting the HD domain of PARP1, demonstrating potent antiviral efficacy in vitro, in vivo, and in human organoids. Our study establishes “phase transition correction” as a novel host‑directed antiviral strategy, with implications beyond IAV to other viruses that manipulate host transcriptional condensates.

## 5 Materials and Methods

### 5.1 Plasmid Construction

#### 5.1.1 pCAG‐NS1‐Flag‐PA

A DNA fragment containing NS1‐Flag was synthesized (Genewiz, China) and inserted into the Xmal I and Nhe I sites in the pCAG‐mNeonGreen‐PA plasmid.

#### 5.1.2 pCAG‐NS1‐mCherry

The DNA fragment NS1 was amplified from the NS1‐Flag template and inserted into the AgeI and Nhe I sites in the pCAG‐mCherry plasmid.

#### 5.1.3 pCAG‐HA‐PARP1(WT/D1/D2/D3/D4/D5D/D6/D7/HD)‐mNeoGreen‐PA

DNA fragments of PARP1 and its mutants were amplified from human cell cDNA and inserted into the Age I and Sal I sites of the pCAG‐mNeonGreen‐PA plasmid.

#### 5.1.4 pCAG‐HA‐SPT5‐TagBFP‐PA

DNA fragments of SPT5‐TagBFP were synthesized (Genewiz, China) and inserted into the AgeI and MfeI sites of the pCAG‐HA‐PARP1‐mNeoGreen‐NLS‐PA plasmid.

#### 5.1.5 pCAG‐HA‐SPT5^−PLD^/SPT5^−PRD^/NELFE^−IDR^/NELFA^−IDR^/AFF4^−IDR^‐TagBFP‐PA

DNA fragments of SPT5^−PLD/^SPT5^−PRD^/NELFE^−IDR^/NELFA^−IDR^/AFF4^−IDR^ were amplified from human cell cDNA and inserted into the Age I and Mlu I sites of the pCAG‐3XHA‐SPT5‐TagBFP‐PA plasmid.

### 5.2 Cell Culture and Transfection

Cells were obtained from the ATCC. HEK293T and A549 cells were cultured in DMEM containing 10% FBS and 1% penicillin/streptomycin at 37°C with 5% CO_2_. For transfection, 1 × 10^5^ cells were seeded into each well of a 24‐well plate one day prior. Polyethyleneimine (23966‐1, Polysciences) was used to transfect cells with 0.5 µg of plasmid DNA per well, with each plasmid used in an equimolar ratio. 12 h after transfection, the medium was replaced with fresh DMEM and cells were incubated for an additional 48 h.

### 5.3 Differentiation of Human Airway and Alveolar Organoids

H9 hESCs were differentiated into hAWOs and hALOs using a staged protocol. Briefly, cells were first treated with Activin A and CHIR99021 in RPMI1640 for 3 days. Subsequently, they were exposed to Noggin, FGF4, CHIR99021, and SB431542 for 4 days to induce foregut fate. The cells were then embedded in Matrigel and cultured with BMP4, all‐trans retinoic acid (ATRA), and CHIR99021 for 7 days to specify lung progenitor identity. Next, lung progenitors were expanded in medium containing FGF10, KGF, and DAPT for 7 days. Finally, hAWOs and hALOs were matured in respective lineage‐specific media with periodic matrigel renewal. Differentiation efficiency was verified by qRT‐PCR and immunofluorescence for lineage markers.

### 5.4 CRISPR Screens

We constructed an RNA‐binding protein CRISPR knockout library with 12 472 gRNAs targeting 1078 genes, based on the lentiCRISPR v2 backbone. The library was packaged into lentivirus using the second‐generation system (pMD2.G and psPAX2) and transduced into A549‐Cas9 cells (MOI = 0.01). After 24 h, cells were selected with puromycin for 2 days, then allowed to recover for 3–5 days. Next, 1 × 10^6^ library cells were infected with PR8 (MOI = 5) for 3 days, with a non‐target control group included. Surviving cells were reseeded, recovered for 3–5 days, and then infected with PR8. This process was repeated for three rounds. After the final round, cells were harvested for Deep‐seq. Genomic DNA was extracted using the TIANamp Genomic DNA Kit. The gRNA insertion sites were amplified via two rounds of PCR: the first round with forward and reverse primers specific to the gRNA site, and the second round with primers containing P5 and P7 sequences to construct the sequencing library. The amplicon libraries were sequenced in PE150 mode.

Raw paired‐end sequencing data were processed using Trim Galore (version 0.6.10) to remove adapter sequences and trim low‐quality bases. Reads were then merged using FLASH (version 1.2.11) to reconstruct full‐length fragments, and unpaired reads were discarded. Subsequently, custom Python scripts were employed to extract the 19‐nucleotide variable sequences. Processed reads were mapped to the reference sgRNA library using MAGeCK to quantify sgRNA abundance. Statistical analysis was performed to evaluate sgRNA depletion or enrichment between experimental conditions, followed by gene‐level scoring to identify significant hits.

### 5.5 IP Mass Spectrometry

Flag‐tagged NS1 (Flag‐NS1) or an empty vector was overexpressed in HEK293T cells for 12 h. Subsequently, the cells were infected with PR8 virus at an MOI of 0.05 for 36 h. Immunoprecipitation was performed using a Flag‐tagged protein immunoprecipitation kit (P2181M, Beyotime). The eluted proteins were mixed with 5X SDS‐PAGE Sample Loading Buffer, heated at 95°C for 5 min, and then subjected to SDS‐PAGE. Gel bands were excised and sent to a company for mass spectrometric analysis. The mass spectrometry data were analyzed using bioinformatics tools. In this pipeline, search engine results were pre‐processed and re‐scored using Percolator to enhance matching accuracy. The output was filtered by a false discovery rate (FDR) of 1% at the spectral level (PSM‐level FDR ≤ 0.01) to generate a list of significantly identified spectra and peptides.

### 5.6 Co‐Immunoprecipitation and Western Blotting

For co‐immunoprecipitation, HEK293T cells co‐transfected with the indicated plasmids for 48 h were immunoprecipitated using Flag (P2181M, Beyotime) or HA (P2185M, Beyotime) kits. Alternatively, A549 cells infected with virus at an MOI of 0.05 were used. Proteins were extracted with RIPA buffer (P0013C, Beyotime) plus inhibitors (ST506, Beyotime) and quantified by BCA assay (P0011, Beyotime). For Western blotting, proteins were separated by 10% SDS‐PAGE, transferred to PVDF membranes, blocked with 5% milk in TBS‐T, and incubated with primary antibodies against HA (51064‐2‐AP, proteintech) or FLAG (66008‐4‐lg, proteintech) or PARP1 (66520‐1‐lg, proteintech) or beta‐Actin (T0022, Affinity) overnight at 4°C. After washing, membranes were incubated with anti‐mouse IgG HRP secondary antibody (S0002‐2, Affinity) or anti‐rabbit IgG HRP secondary antibody (S0001‐2, Affinity) for 1 h, and proteins were detected with ECL substrate and imaged.

### 5.7 Immunofluorescence Staining

For immunofluorescence staining, cells or tissue sections were fixed in 4% paraformaldehyde for 15 min at room temperature, followed by permeabilization with 0.1% Triton X‐100 for 10 min. Non‐specific binding was blocked with 5% bovine serum albumin (BSA) for 1 h at room temperature. Subsequently, the samples were incubated with primary antibodies against PARP1 (66520‐1‐lg, Proteintech), AQP5 (20334‐1‐AP, Proteintech), proSP‐C (AB3786, EMD‐Millipore), Mucin 5AC (ab3649, Abcam), CC10 (10490‐1‐AP, Proteintech), NP (11675‐MM03T, Sino Biological), or NP (11675‐T62, Sino Biological) overnight at 4°C. After three washes with phosphate‐buffered saline (PBS), the samples were incubated with secondary antibodies for 1 h at room temperature. The secondary antibodies used were Goat anti‐Rabbit IgG (H+L)‐Alexa Fluor 488 (R37116, Invitrogen), Goat anti‐Rabbit IgG (H+L)‐Alexa Fluor 594 (R37117, Invitrogen), Goat anti‐Rabbit IgG (H+L)‐Alexa Fluor 647 (A‐21245, Invitrogen), Goat anti‐Mouse IgG (H+L)‐Alexa Fluor 488 (A‐11001, Invitrogen), Goat anti‐Mouse IgG (H+L)‐Alexa Fluor 594 (A‐11005, Invitrogen), or Goat anti‐Mouse IgG (H+L)‐Alexa Fluor 647 (A‐21235, Invitrogen). Nuclei were counterstained with DAPI for 5 min. Coverslips were mounted onto slides using Prolong Gold antifade reagent. Images were captured using a Nikon A1 confocal microscope.

### 5.8 FRAP and Live‐Cell Imaging

HEK293T cells grown on a glass bottom microwell dish were transfected with 2 µg of plasmids encoding the fluorescently tagged proteins. At 48 h after transfection, cells were imaged. During image acquisition, cells were maintained in DMEM supplemented with 25 mM HEPES. FRAP experiments were performed using a Nikon A1 confocal microscope with a ×60 oil objective, as described in our previous study [45]. Briefly, a single droplet was photobleached using a 488 nm laser. Fluorescence recovery was tracked for 50 frames post‐bleaching. Intensities from the bleached region (Fs), an unbleached control region (Fc), and background (Fb) were measured. The photobleaching rate (r) was calculated as (r = Fc/Fc0), normalized fluorescence intensity (F) as (F = [Fs – Fb]/r), and mobile fraction (Fm) as (Fm = F∞/F0). Time‐lapse imaging was conducted to observe the dynamic behaviors of droplets, with images captured every 50 s over a period of 1 h, and fluorescence intensity was analyzed using ImageJ software.

### 5.9 Cellular Thermal Shift Assay (CETSA)

CETSA was performed in cellular lysates to evaluate target engagement through thermal stabilization. Briefly, harvested cells were lysed in buffer containing protease inhibitors, and the clarified supernatant was divided into two aliquots. These were treated with either DMSO (vehicle) or the test compound (1 mM final concentration) at 4°C for 1 h. Aliquots of each lysate were then heated across a temperature gradient (37–52°C) for 6 min in a thermal cycler, cooled, and centrifuged. The soluble fractions were collected, denatured with SDS‐PAGE loading buffer at 100°C, and analyzed by Western blot. Band intensities were quantified to generate thermal denaturation curves, comparing target protein stability between compound‐treated and control samples.

### 5.10 Viral Infection and Compound Treatment

Prior to infection, differentiated 3D organoids (hAWOs and hALOs) of comparable dimensions were meticulously selected to ensure inter‑group uniformity. Matrigel was enzymatically dissociated using Cell Recovery Solution (Corning, 354253), and the released organoids were resuspended in culture medium to generate a homogeneous single‑organoid suspension, which was then equally aliquoted across all experimental groups. This rigorous normalization procedure guaranteed that any subsequent heterogeneity in organoid size, number, or cellular composition was attributable to infection‑induced phenotypes rather than to pre‑existing variations.

For infection and compound treatment, A549 cell, hAWOs and hALOs were infected with IAV at a multiplicity of infection (MOI) of 0.05. Following a 2 h adsorption period, the inoculum was removed and replaced with fresh organoid medium. For antiviral assessment, the cell lines and organoids were pretreated with TEC (2 µg mL^−^^1^) or IFA (10 µg mL^−^^1^) for 12 h before viral infection. At 24 h post‐infection, the cell lines and organoids were collected for downstream analyses. Viral replication was assessed by qPCR and by TCID_50_ assay. Host antiviral and inflammatory responses were evaluated via qPCR of relevant genes and immunofluorescence staining for viral protein. Cell viability was assessed using the MTT assay. Organoid morphology, number, and size were quantified using ImageJ.

### 5.11 Mouse Models

Female BALB/c mice (6–7 weeks old, 16–18 g) were purchased from Vital River Laboratory Animal Technology (Beijing, China). Mice were pre‐treated with the drug via gavage for 2 days (25 mg/kg for TEC and 20 mg/kg for IFA). Subsequently, mice were anesthetized with isoflurane and intranasally inoculated with 1.5×LD50 of virus (50 µL per mouse). Drug treatment via gavage continued throughout the experiment. Mice were monitored daily for body weight and clinical symptoms. A 35% body weight loss was considered as death. On day 5 post‐inoculation, mice were euthanized, and lung tissues were collected for further analysis. Left Lung: Homogenized and centrifuged. The supernatant was used for virus titer determination by TCID50 assay on MDCK cells. The pellet was used for RNA extraction and subsequent QPCR analysis. Right Lung: Divided into two portions. One portion was fixed in 4% paraformaldehyde for histopathological analysis using H&E staining. The other portion was stored at ‐80°C for future use. Animal experiments were conducted with ethics approval (GZLAB‐AUCP‐2023‐10‐A5).

### 5.12 ATAC‐Seq Library Preparation and Data Processing

A total of 50 000 cells were harvested, washed with cold PBS, and lysed to isolate nuclei. Genomic DNA was tagmented using Tn5 Transposase at 37°C for 30 min. Following purification, the DNA fragments were amplified by PCR to construct sequencing libraries, which were then sequenced on an Illumina platform.

Raw sequencing reads were trimmed and filtered using Cutadapt to obtain high‐quality clean reads, which were aligned to the reference genome using Bowtie2. Sample correlation was assessed by calculating Pearson correlation coefficients based on read counts in 10‐kb genomic bins. Peaks were called using MACS2 (FDR < 0.05) and annotated to genomic features with ChIPseeker. Functional enrichment analyses (GO and KEGG) for promoter‐associated peak‐linked genes were performed using clusterProfiler. Motif discovery was conducted with MEME‐ChIP and Tomtom. Differential chromatin accessibility analysis was performed using DiffBind and DESeq2 (|fold change| > 1.5, *p* < 0.05), followed by functional and motif enrichment analyses of the differential peaks.

### 5.13 ChIP‐PCR

ChIP‑PCR was performed using a ChIP Kit (Beyotime, P2080) according to the manufacturer's instructions. Cells (8 × 10^6^) cultured on 10‑cm dishes were cross‐linked, and chromatin was sheared to 200–1000 bp with a sonicator (Bioruptor PICO). Immunoprecipitation employed an anti‑Rpb1 NTD (D8L4Y) (Cell Signaling Technology, 14958) or an isotype IgG control (proteintech, 98136‐1‐RR). The precipitated DNA and a diluted aliquot of the input chromatin were quantified by qPCR using SYBR Premix Ex Taq II on a CFX96 Touch system (Bio‑Rad). Relative enrichment for each target was calculated by normalization to the input control.

### 5.14 RNA‐Seq

RNA was isolated using the RNA rapid extraction reagent (400‐100, Goonie). The constructed sequencing libraries were sequenced on the Illumina HiSeq platform with 150 bp paired‐end reads. The raw paired‐end reads were preprocessed to remove adapters using Cutadapt (version 1.9.1). The reads were mapped to the human genome (hg38) using the HISAT2 aligner (version 2.2.1). Gene expression levels were calculated using HTSeq (version 0.6.1), employing the FPKM (Fragments Per Kilobase of transcript per Million mapped reads) method. Differential gene expression analysis was performed using DESeq2 (version 1.26.0) from the Bioconductor package. Genes with a fold change of at least 2 and an adjusted *p*‐value (*q*‐value, FDR) of ≤ 0.05 were considered significantly differentially expressed. Gene ontology (GO) enrichment analysis was conducted using GOseq, and pathway analysis was performed using the KEGG (Kyoto Encyclopedia of Genes and Genomes) database.

### 5.15 RNA‐Extraction and qPCR

RNA was extracted using the RNA rapid extraction reagent (400‐100, Goonie). Subsequently, 500 ng of RNA was reverse‐transcribed into cDNA using the Evo M‐MLV Reverse Transcription Kit II (AG11711, AG). RT–qPCR was conducted on a CFX96 Touch Real‐Time PCR Detection System (Bio‐Rad) with SYBR Green Pro Taq HS Premix qPCR Kit III (AG11739, AG). The ΔΔCt method was utilized to quantify relative gene expression levels after normalization to glyceraldehyde‐3‐phosphate dehydrogenase (GAPDH) expression. For the detection of IAV‐specific mRNA and vRNA, we followed a previously reported protocol [46].

### 5.16 Statistics Analysis

Statistical analysis was performed using GraphPad Prism 10.1.2 (GraphPad Software). For in vitro experiments (cell lines and organoids), data are presented as mean ± SD from at least three independent biological replicates (n = 3). For in vivo experiments, data are presented as mean ± SD, with n = 6 mice per group. Data were analyzed using two‐tailed unpaired Student's t‐test for comparisons between two groups and one‐way ANOVA followed by Tukey's multiple comparison test for comparisons among multiple groups. For paired samples, a two‐tailed paired Student's t‐test was used. *p < 0.05, **p < 0.01, ***p < 0.002, ****p < 0.001, ns, *p* ≥ 0.05.

## Author Contributions

Z.Y., K.W., and S.M. conceived the idea, designed the experiments, analyzed the data, and wrote the manuscript. S.M. and C.K. performed most of the experiments. X.L. and M.L. conducted FRAP, live‐cell imaging, H&E staining, immunofluorescence staining, and statistical analysis. L.Y. was responsible for cell culture, plasmid construction, and transfection. Y.Y. performed bioinformatics analyses. J.Z. and Y.L. conducted molecular docking. G.J. performed some organoid cultures. Y.L. and X.C. conducted some IAV virus infection models. S.M. and X.C. provided funding support.

## Conflicts of Interest

The authors declare no conflicts of interest.

## Supporting information




**Supporting File 1**: advs77883‐sup‐0001‐SuppMat.pdf.

**Supporting File 2**: advs77883‐sup‐0002‐Table1.pdf.

**Supporting File 3**: advs77883‐sup‐0003‐Table2.pdf.

**Supporting File 4**: advs77883‐sup‐0004‐Table3.pdf.

## Data Availability

All primer sequences are available in Table S1. NS1 IP mass spectrometry gene lists are available in Table S2. The RNA‐seq data have been deposited on the National Center for Biotechnology Information database GSE299052. The ATAC‐seq raw sequence data have been deposited in the Genome Sequence Archive in the National Genomics Data Center, China National Center for Bioinformation / Beijing Institute of Genomics, Chinese Academy of Sciences (GSA‐Human: HRA016711) that are publicly accessible at https://ngdc.cncb.ac.cn/gsa‐human.
