# Supplementary material for: NS1‐Induced PARP1 Phase Transitions Prolongs Host Transcriptional Pausing to Promote Influenza A Virus Replication
Source: Adv Sci (Weinh). 2026 Sep 27:e77883. Online ahead of print. doi: 10.1002/advs.77883 (PMC13616264; doi:10.1002/advs.77883)
Supplement: Supplementary file 1 [file ADVS-9999-e77883-s001.pdf]

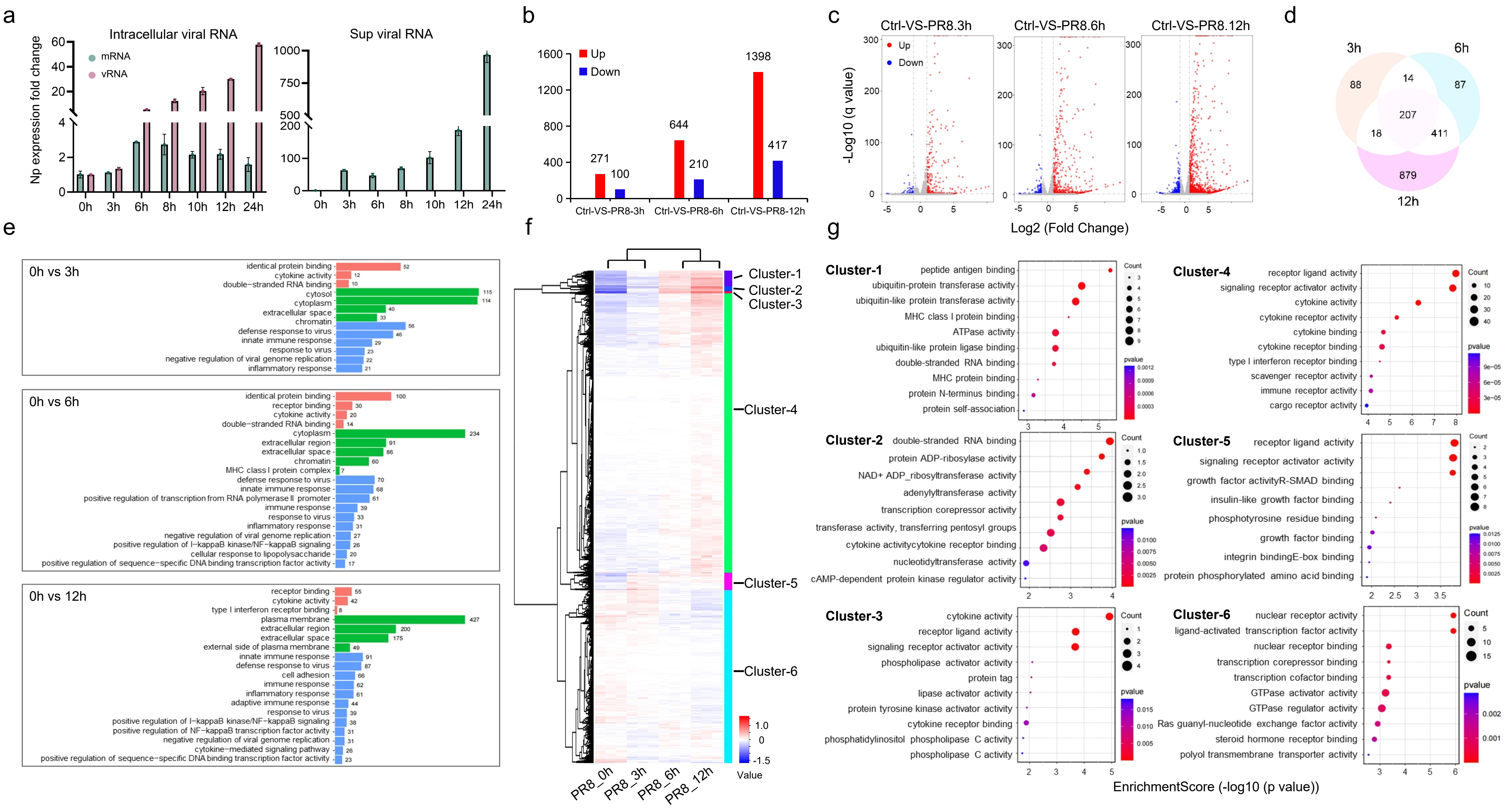

Supplementary Figure 1. Cell transcription is altered upon IAV infection

Supplementary Figure 1. Cell transcription is altered upon IAV infection. (a) RT-PCR analysis of viral mRNA and vRNA levels in PR8-infected A549 cells at 0, 3, 6, 8, 10, 12, and 24 hpi. (b-d) RNA-Seq analysis of PR8-infected A549 cells at 3, 6, and 12 hpi. (b) Number of differentially expressed genes. (c) Volcano plot of gene expression changes. (d) Venn diagram of overlapping differentially expressed genes. (E) KEGG pathway enrichment analysis. (f, g) Hierarchical clustering and pathway analysis of gene expression patterns (6 clusters). Mean values were presented with SD, n = 3 biological triplicates.

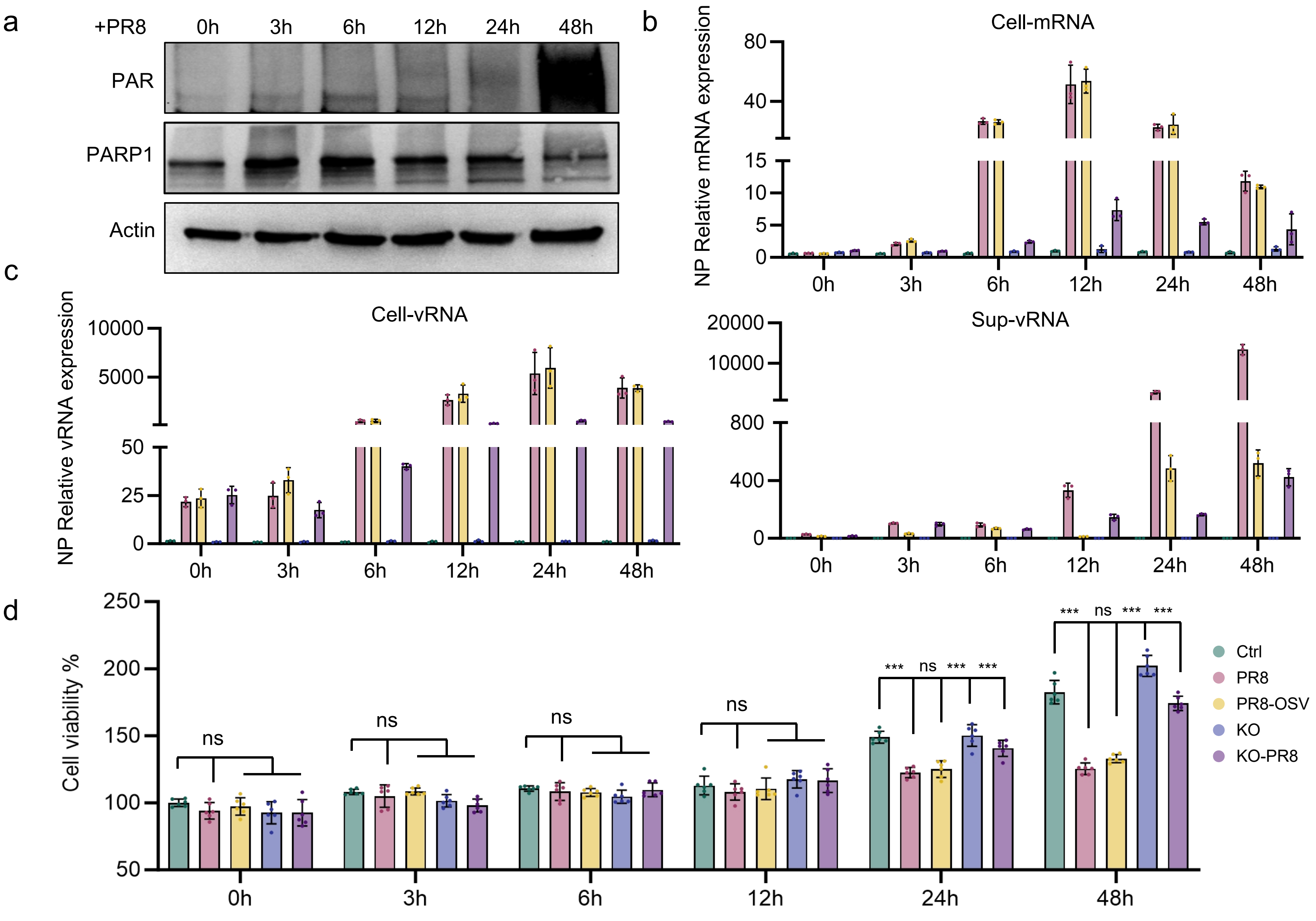

Supplementary Figure 2. Host factor PARP1 is associated with IAV transcription

Supplementary Figure 2. Host factor PARP1 is associated with IAV transcription. (a) WB analysis of endogenous PARP1 protein and PAR levels in PR8-infected A549 cells at 3, 6, and 12 hpi. (b-c) RT-PCR analysis of viral mRNA and vRNA levels in PR8-infected A549 cells or PARP1-knockout A549 cells (KO) at 3, 6, and 12 hpi. (d) MTT assay for cell viability. Mean values were presented with SD, n = 3 biological triplicates. One-way ANOVA test, ***$p < 0.002$, ns, not significant.

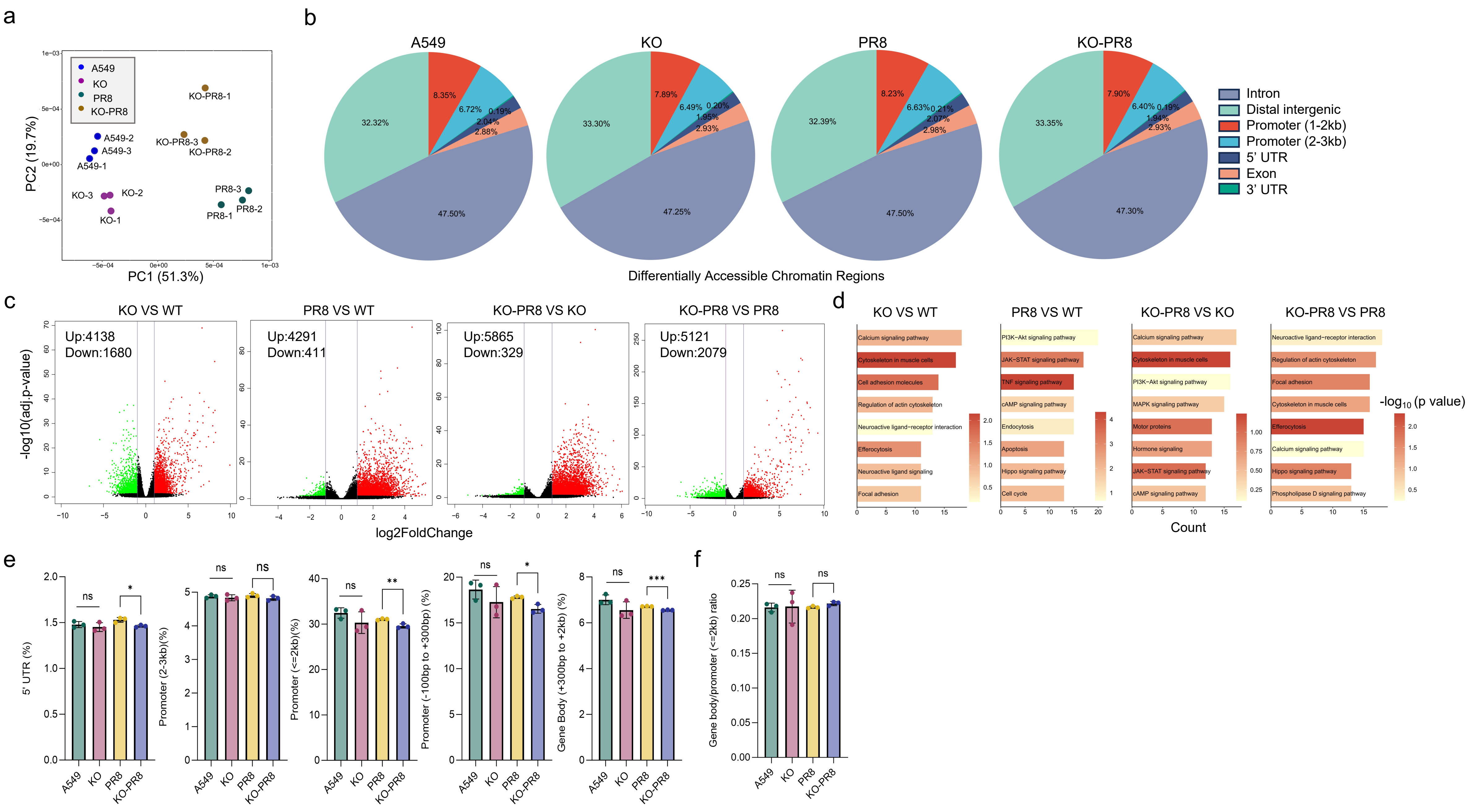

Supplementary Figure 3. Landscape of chromatin accessibility across experimental conditions

Supplementary Figure 3. Landscape of chromatin accessibility across experimental conditions. (a) Principal component analysis (PCA) of accessible chromatin regions determined by ATAC-seq, with each point representing an independent biological replicate. (b) Genomic annotation of consensus peaks across all samples, showing distribution relative to gene features. (c) Volcano plot depicting differentially accessible regions (DARs), with statistical significance ($-\log_{10}$ P-value) plotted against fold change. (d) Pathway enrichment analysis of DARs across four pairwise comparisons: KO versus A549 (genetic knockout effect), PR8 versus A549 (viral infection response), KO-PR8 versus KO (viral infection in knockout background), and KO-PR8 versus PR8 (genetic modification during infection). Significantly enriched pathways (FDR < 0.05) are shown for each contrast. (e) Fraction of total peaks mapping to 5’UTR, promoter (≤3 kb from TSS) and gene body (+300bp to +2000bp) regions for each condition. (f) Ratio of gene-body accessibility to promoter accessibility for each condition. A549: Wild-type A549 cells without viral infection. PR8: Wild-type A549 cells infected with PR8 virus. KO: PARP1 knockout A549 cell line without viral infection. KO-PR8: PARP1 knockout cell line infected with PR8 virus. Mean values were presented with SD, n = 3 biological triplicates. One-way ANOVA test, **p < 0.01, ***p < 0.002, ns, not significan.

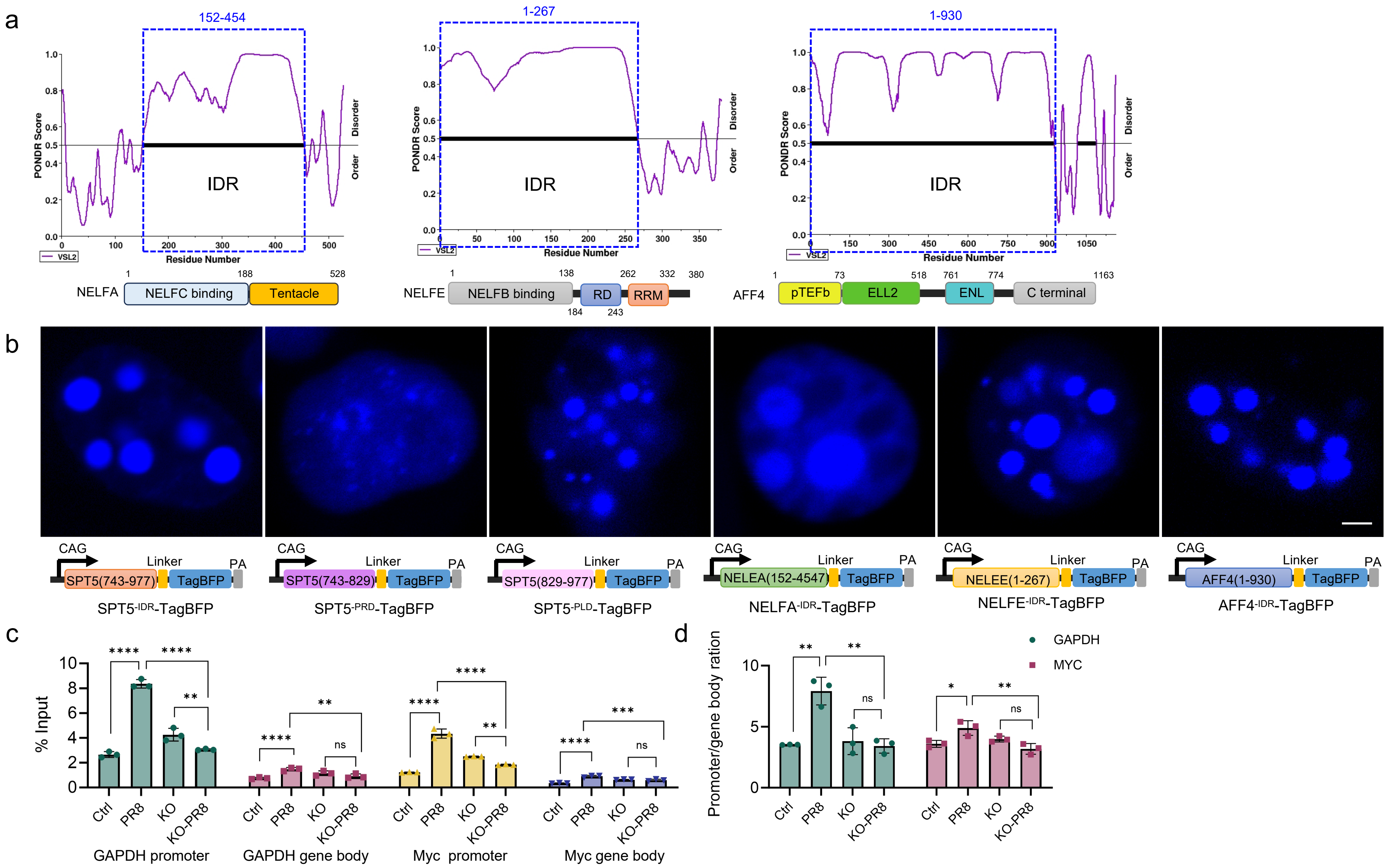

Supplementary Figure 4. Transcription pause-release regulatory factors form phase separation

Supplementary Figure 4. Transcription pause-release regulatory factors form phase separation. (a) IDR structures of SPT5, NELFA, NELFE, and AFF4 were predicted using PONDR-VSL2, with blue boxes highlighting the IDR domains used in this study. (b) IDR-TagBFP expression vectors were constructed and transfected into 293T cells, followed by fluorescence observation after 12 hours. Scale bar, 2μm. (c) ChIP-qPCR to confirm increased retention of Pol II in the transcriptional pause phase. (d) Ratio of promoter to gene-body. A549: Wild-type A549 cells without viral infection. PR8: Wild-type A549 cells infected with PR8 virus. KO: PARP1 knockout A549 cell line without viral infection. KO-PR8: PARP1 knockout cell line infected with PR8 virus. Mean values were presented with SD, n = 3 biological triplicates. One-way ANOVA test, $*p < 0.05$, $**p < 0.01$, $***p < 0.002$, $****p < 0.001$, ns, not significan.

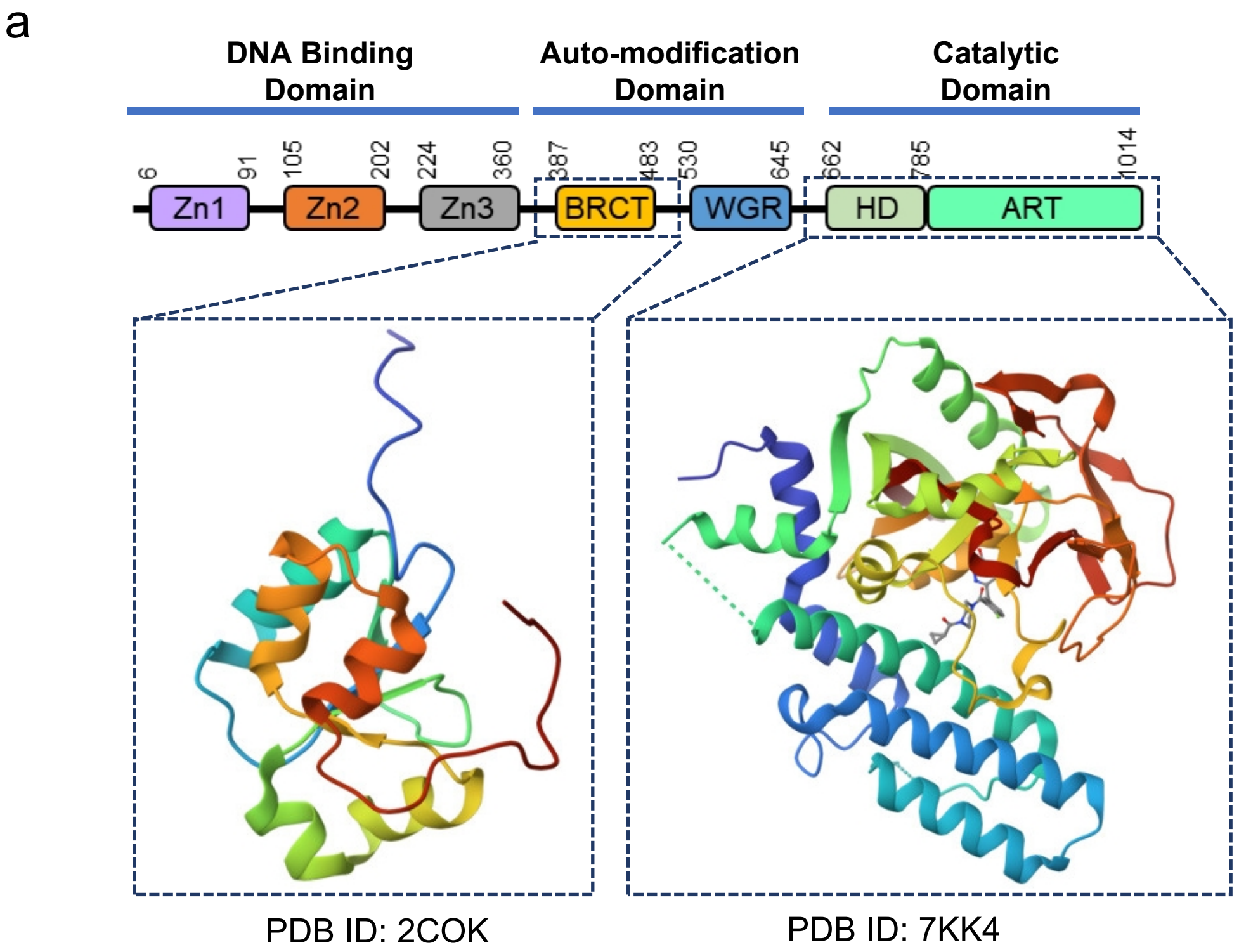

Inhibitor	Function
Olaprid	PARP1/2, majorly targets the ART domain
AG14361	PARP1, majorly targets the ART domain
Gossypol	BRCT domain-targeting PARP1
NAD+	The substrate for PARP1

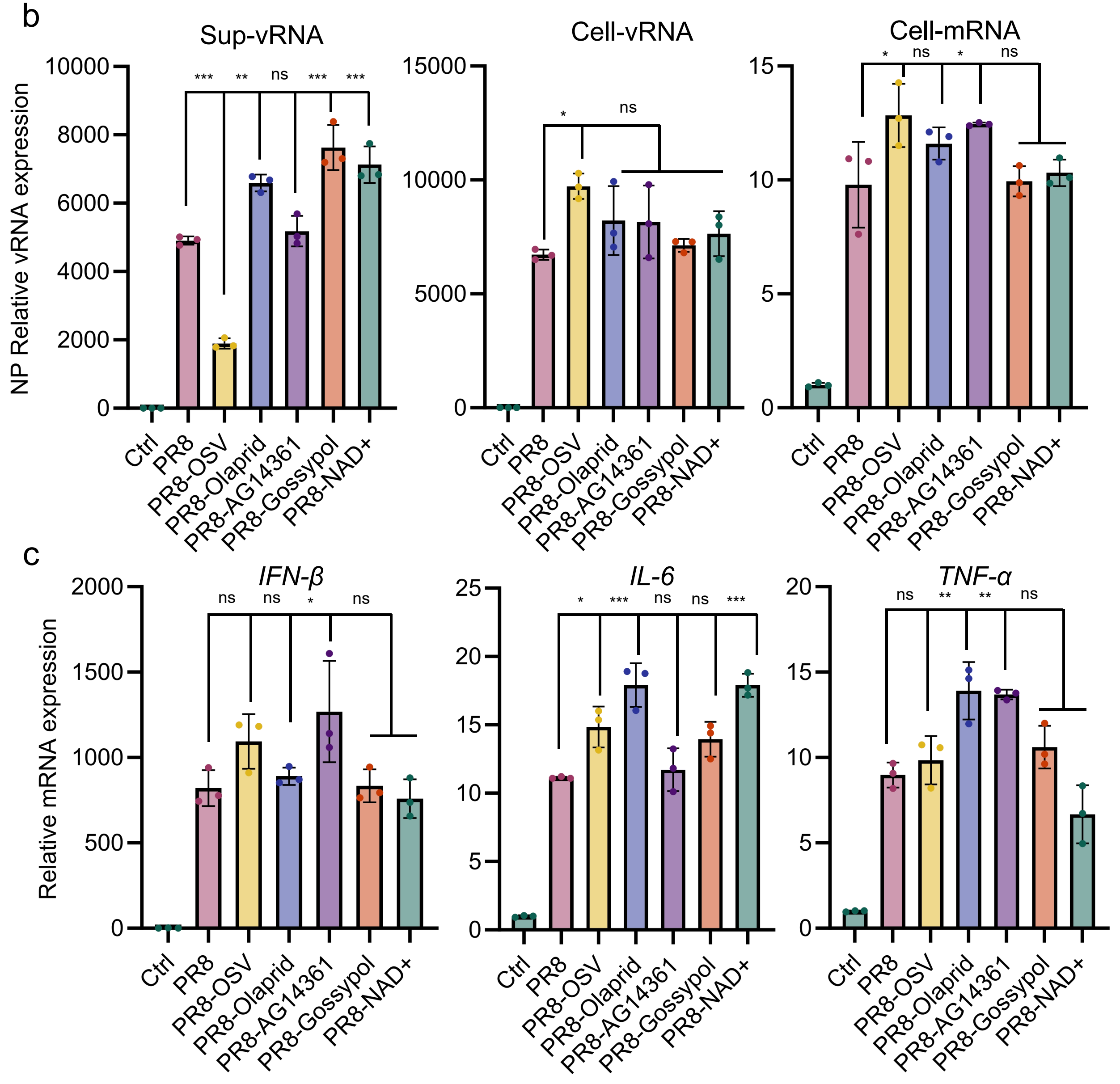

Supplementary Figure 5. PARP1 inhibitors (Olaparib, AG14361, Gossypol) and substrate NAD^+ are ineffective in PR8-infected A549 cells

Supplementary Figure 5. PARP1 inhibitors (Olaparib, AG14361, Gossypol) and substrate NAD^+ are ineffective in PR8-infected A549 cells. (a) Structure of PARP1 domains and representative inhibitors. (b, c) RT-PCR analysis of viral RNA levels and host inflammatory cytokine expression in PR8-infected A549 cells under indicated treatments. Mean values were presented with SD, n = 3 biological triplicates. One-way ANOVA test, *$p < 0.05$, **$p < 0.01$, ***$p < 0.002$, ns, not significant.

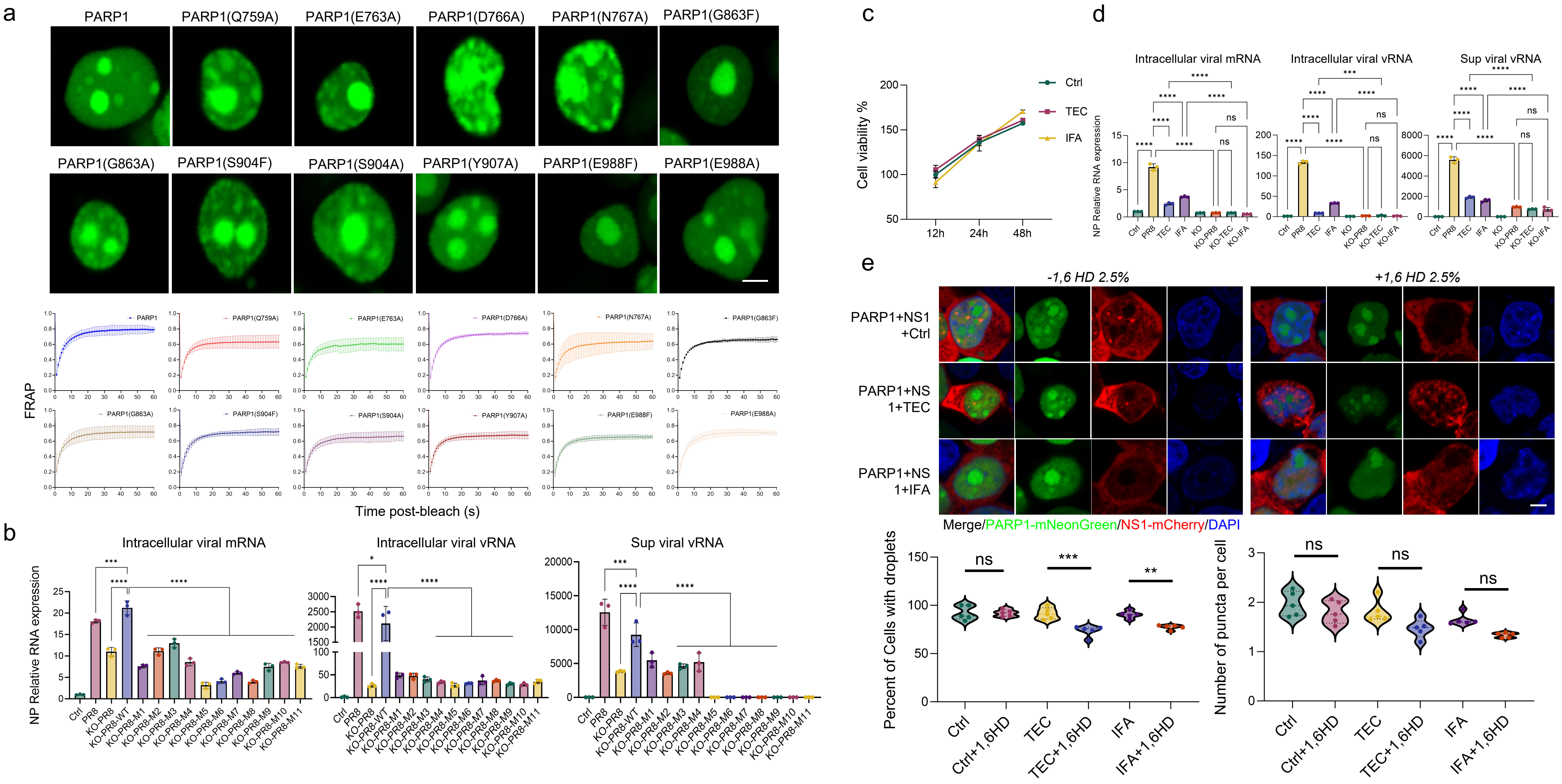

Supplementary Figure 6. TEC and IFA promote the mobility of PARP1 phase separation induced by NS1

Supplementary Figure 6. TEC and IFA promote the mobility of PARP1 phase separation induced by NS1. (a) HEK293T cells were transfected with plasmids encoding mNeonGreen‐ tagged wild‐ type (WT) or mutant PARP1 (Q759A, E763A, D766A, N767A, G863F, G863A, S904F, S904A, Y907A, E988F, E988A) for 24 h. Top: fluorescence images showing condensate formation. Bottom: FRAP analysis of condensate dynamics for the indicated constructs. Data are plotted as means ± SD (n = 6). Scale bar, 5μm. (b) qPCR quantification of viral RNA in rescue experiments. PARP1‐ knockout cells were reconstituted with either wild‐ type PARP1 or the indicated point mutants (M1–M11, corresponding to Q759A, E763A, D766A, N767A, G863F, G863A, S904F, S904A, Y907A, E988F, and E988A, respectively). (c) MTT assay of cell viability in A549 cells treated with TEC or IFA at 12h, 24 and 48 h post‐ infection. (d) RT-PCR analysis of viral RNA levels in PR8-infected A549 cells or PARP1-KO cells under indicated treatments. (e) Cells were treated with 2.5% 1,6-hexanediol for 10 min to observe condensate morphology and quantify the number of condensates per cell and the proportion of cells with condensates. Mean values were presented with SD, n = 3 biological triplicates. One-way ANOVA test, , *p < 0.05, **p < 0.01, ***p < 0.002, ****p < 0.001, ns, not significant. Scale bar, 5μm.

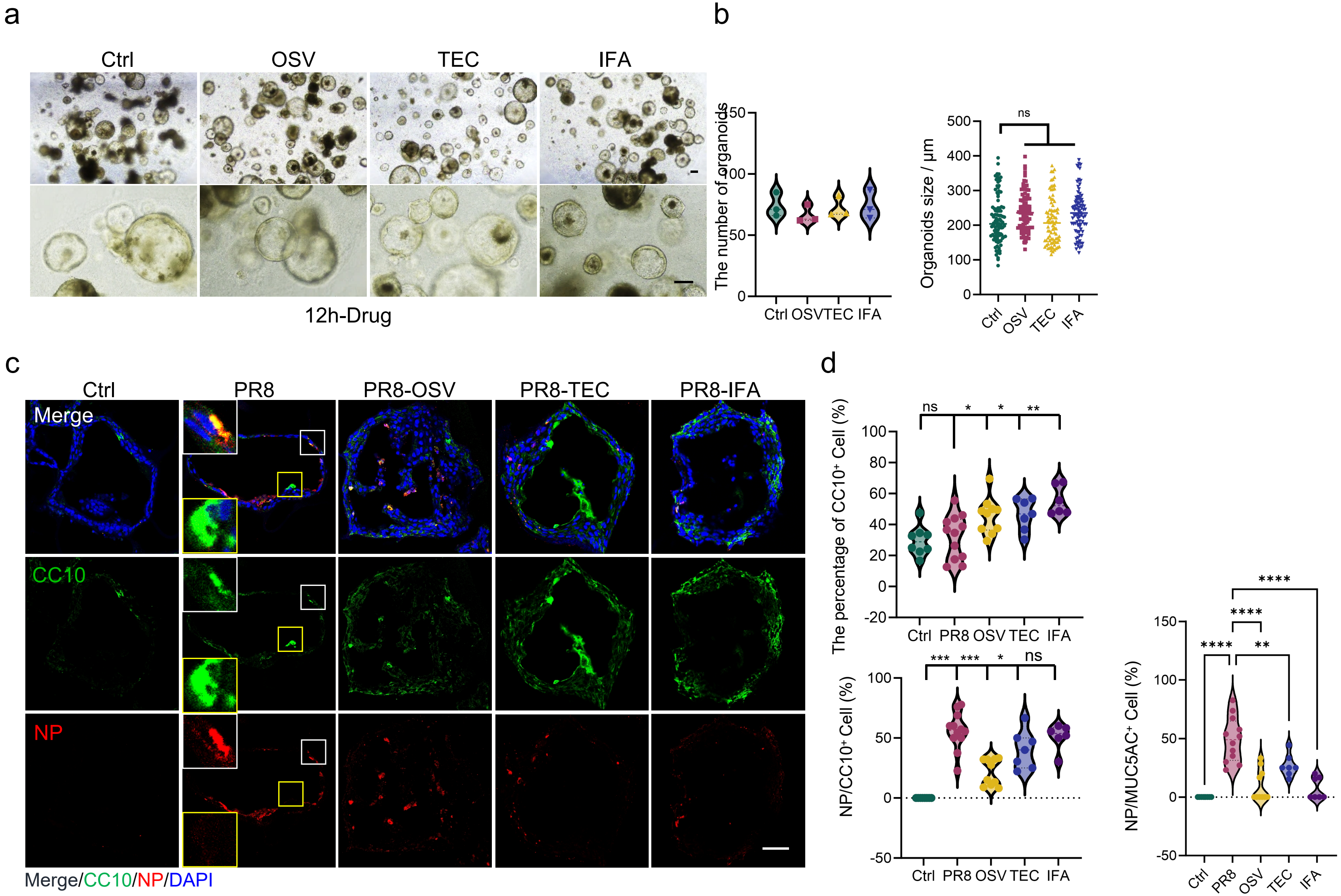

Supplementary Figure 7. Effects of TEC and IFA pre-treatment on airway organoids

Supplementary Figure 7. Effects of TEC and IFA pre-treatment on airway organoids. (a, b) Morphology of airway organoids (AWO) pre-treated with TEC and IFA. Size and number of organoids were quantified. $n \geq 80$ organoids. (c) immunofluorescence staining of viral NP and Club cells. Scale bar, 50μm. (d) Quantification of the proportion of corresponding cells and NP positivity rate. $n \geq 6$ organoids. Mean values were presented with SD, $n = 3$ biological triplicates. One-way ANOVA test, $^{*}p < 0.05$, $^{**}p < 0.01$, $^{***}p < 0.002$, ns, not significant.

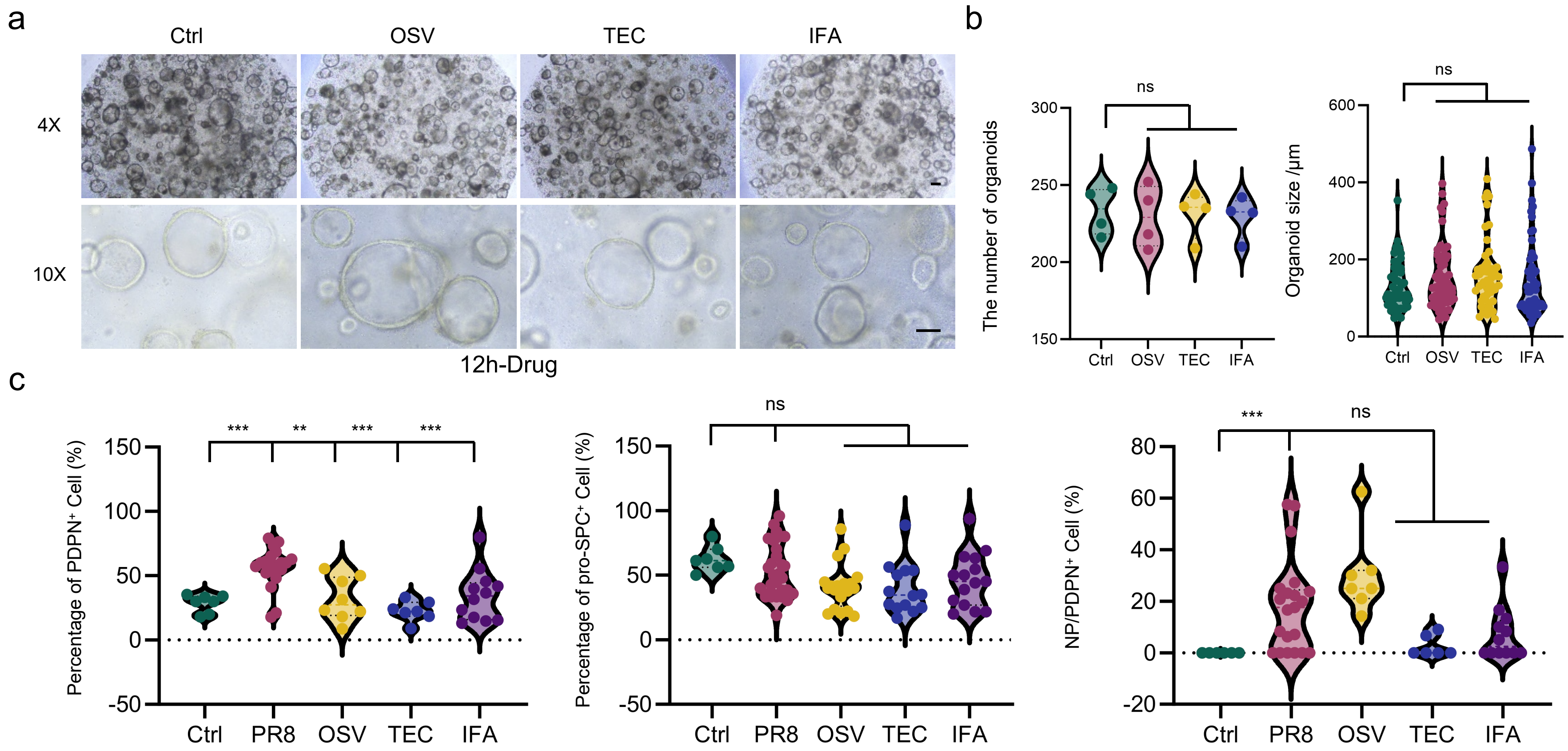

Supplementary Figure 8. Effects of TEC and IFA pre-treatment on alveolar organoids

Supplementary Figure 8. Effects of TEC and IFA pre-treatment on alveolar organoids. (a, b) Morphology of ALO pre-treated with TEC and IFA. Size and number of organoids were quantified. n ≥ 50 organoids. Scale bar, 200μm. (c) Quantification of the proportion of corresponding cells and NP positivity rate, after immunofluorescence staining of viral NP and AT1 ($PDPN^+$) and AT2 ($Pro\text{-}SPC^+$). n ≥ 7 organoids. Mean values were presented with SD, n = 3 biological triplicates. One-way ANOVA test, **p < 0.01, ***p < 0.002, ns, not significant.
