# Supplementary material for: NS1‐Induced PARP1 Phase Transitions Prolongs Host Transcriptional Pausing to Promote Influenza A Virus Replication
Source: Adv Sci (Weinh). 2026 Sep 27:e77883. Online ahead of print. doi: 10.1002/advs.77883 (PMC13616264; doi:10.1002/advs.77883)
Supplement: Supplementary file 3 [file ADVS-9999-e77883-s003.pdf]

Supplementary Table 1. The sequence information of primers	
**RT-PCR Primers**	**Sequence (5'-3')**
RT-NP-mRNA (RT)	CCAGATCGTTCGAGTCGTTTTTTTTTTTTTTTTTCTTTAATTGTC
RT-NP-vRNA (RT)	GGCCGTCATGGTGGCGAATGAATGGACGGAGAACAAGGATTGC
RT-GAPDHtag	GCATTATCGATCAGTACCAGTCGTACCACCCTGTTGCTGTAGCCAA
NP(mRNA)-F	CGATCGTGCCCTCCTTTG
NP(mRNA)-R	CCAGATCGTTCGAGTCGT
NP(vRNA)-F	GGCCGTCATGGTGGCGAAT
NP(vRNA)-R	CTCAATATGAGTGCAGACCGTGCT
qGAPDH-F	GTCTCCTCTGACTTCAACAGCG
qGAPDHtag-R	GCATTATCGATCAGTACCAGTCGT
IL-6-F1	AGACAGCCACTCACCTCTTCAG
IL-6-R1	TTCTGCCAGTGCCTCTTTGCTG
TNF-α-F1	CTCTTCTGCCTGCTGCACTTTG
TNF-α-R1	ATGGGCTACAGGCTTGTCACTC
IFN-β-F1	CTTGGATTCCTACAAAGAAGCAGC
IFN-β-R1	TCCTCCTTCTGGAACTGCTGCA
mIL-6-F1	TACCACTTCACAAGTCGGAGGC
mIL-6-R1	CTGCAAGTGCATCATCGTTGTTC
mIL-8-F1	GGTGATATTCGAGACCATTTACTG
mIL-8-R1	GCCAACAGTAGCCTTCACCCAT
mTNF-α-F1	GGTGCCTATGTCTCAGCCTCTT
mTNF-α-R1	GCCATAGAACTGATGAGAGGGAG
mIL-1β-F1	TGGACCTTCCAGGATGAGGACA
mIL-1β-R1	GTTCATCTCGGAGCCTGTAGTG
mIFN-β-F1	GCCTTTGCCATCCAAGAGATGC
mIFN-β-R1	ACACTGTCTGCTGGTGGAGTTC
