# Supplementary material for: NS1‐Induced PARP1 Phase Transitions Prolongs Host Transcriptional Pausing to Promote Influenza A Virus Replication
Source: Adv Sci (Weinh). 2026 Sep 27:e77883. Online ahead of print. doi: 10.1002/advs.77883 (PMC13616264; doi:10.1002/advs.77883)
Supplement: Supplementary file 4 [file ADVS-9999-e77883-s004.pdf]

Supplementary Table 3. CRISPR screen gene list

Gene	beta	z	wald-p-value	wald-fdr	Chrom
SFSWAP	-2.0491	-4.4879	7.1939e-06	0.0032436	chr5
RBMS3	-1.4427	-4.4212	9.8137e-06	0.0032436	chr9
IARS	-2.5795	-4.3939	1.1133e-05	0.0032436	chr13
ZFP36L2	-1.5095	-4.3695	1.2455e-05	0.0032436	chr17
PURB	-2.021	-4.3533	1.341e-05	0.0032436	chr11
RBM22	-2.1482	-4.3279	1.5055e-05	0.0032436	chr18
MRPL43	-1.2369	-4.2498	2.1399e-05	0.0033344	chr19
DHX40	-1.7717	-4.2252	2.3877e-05	0.0033344	chr11
ZC3HAV1L	-2.5191	-4.224	2.3999e-05	0.0033344	chr6
MRPL46	-1.4644	-4.2159	2.4876e-05	0.0033344	chr7
RPLP1	-1.9057	-4.2038	2.6247e-05	0.0033344	chr9
CARS2	-1.8971	-4.1967	2.7082e-05	0.0033344	chr8
MRPS9	-2.6427	-4.1931	2.7514e-05	0.0033344	chr2
KHSRP	-2.5764	-4.1666	3.0913e-05	0.0035102	chr17
POP7	-1.3154	-4.1542	3.2649e-05	0.0035102	chr5
PUS1	-2.6208	-4.1422	3.4396e-05	0.0035102	chr5
SRP72	-1.2058	-4.1085	3.9821e-05	0.0038102	chr5
HNRNPUL1	-2.3463	-4.0867	4.376e-05	0.0038102	chr7
HNRNPH3	-1.8794	-4.0634	4.8366e-05	0.0038102	chr10
GTPBP4	-1.8645	-4.0577	4.9555e-05	0.0038102	chr13
BOP1	-2.1265	-4.0486	5.1517e-05	0.0038102	chr15
PRPF40B	-1.9348	-4.0418	5.3035e-05	0.0038102	chr15
EIF2B2	-1.4814	-4.0247	5.7039e-05	0.0038102	chr12
RBMY1A1	-2.0831	-4.0013	6.2999e-05	0.0038102	chrY
NUPL2	-1.2638	-3.9994	6.3495e-05	0.0038102	chr5
RPS27L	-2.0584	-3.9852	6.7417e-05	0.0038102	chr9
MTO1	-1.2595	-3.9846	6.7584e-05	0.0038102	chr9
PAPOLA	-2.3882	-3.9837	6.7846e-05	0.0038102	chr12
CPEB3	-1.8782	-3.9748	7.043e-05	0.0038102	chr2
INTS12	-1.363	-3.9723	7.1179e-05	0.0038102	chr3
RPUSD1	-1.522	-3.9675	7.2637e-05	0.0038102	chr17
RPL22	-1.8716	-3.9545	7.6695e-05	0.0038102	chr4
ADAD2	-2.6081	-3.9463	7.9362e-05	0.0038102	chr8
RPUSD4	-1.4141	-3.9184	8.9127e-05	0.0038102	chr9
MTIF3	-1.4823	-3.9013	9.567e-05	0.0038102	chr5
SRSF3	-1.3888	-3.8898	0.00010033	0.0038102	chr17
HARS	-1.4815	-3.874	0.00010708	0.0038102	chr18
TARS	-1.5246	-3.8609	0.00011298	0.0038102	chr15
RBM26	-2.1155	-3.8572	0.00011471	0.0038102	chr14
ATXN1	-1.6776	-3.8456	0.00012026	0.0038102	chr13
PAN2	-1.4353	-3.8445	0.00012082	0.0038102	chr7
EIF4H	-1.9235	-3.8391	0.00012347	0.0038102	chr5
CHERP	-2.1628	-3.8357	0.00012521	0.0038102	chr8
SRSF5	-1.1706	-3.8328	0.00012669	0.0038102	chr12
EEF1A2	-1.08	-3.8298	0.00012823	0.0038102	chr2
INTS1	-1.1113	-3.8247	0.00013093	0.0038102	chr14
PAN3	-1.5443	-3.8234	0.00013162	0.0038102	chr5
PCF11	-1.8747	-3.82	0.00013347	0.0038102	chr7
PABPC4L	-2.5321	-3.8165	0.00013538	0.0038102	chr3

MAGOHB	-1.3635	-3.8102	0.00013887	0.0038102	chr6
RBM38	-2.8631	-3.8015	0.00014385	0.0038102	chr2
RBMY1E	-1.6051	-3.7998	0.00014479	0.0038102	chrY
GATC	-2.0103	-3.7931	0.00014879	0.0038102	chr5
TSEN34	-2.1014	-3.7871	0.00015241	0.0038102	chr7
MSI1	-2.5354	-3.7858	0.00015321	0.0038102	chr5
EIF2D	-1.5848	-3.7842	0.00015422	0.0038102	chr6
POLR2A	-1.5002	-3.7841	0.00015426	0.0038102	chr11
RNASE10	-1.9741	-3.7818	0.00015567	0.0038102	chr16
ZNF346	-2.4793	-3.7759	0.00015942	0.0038102	chr5
YTHDC2	-1.1713	-3.7709	0.00016263	0.0038102	chr18
SNRPF	-1.0785	-3.7703	0.00016306	0.0038102	chr10
ADARB2	-1.7974	-3.7652	0.00016644	0.0038102	chr13
ABT1	-1.6431	-3.765	0.00016655	0.0038102	chr13
NCBP2L	-2.137	-3.7632	0.00016775	0.0038102	chrX
LIN28A	-1.3613	-3.7625	0.0001682	0.0038102	chr4
AIMP2	-1.583	-3.76	0.00016994	0.0038102	chr5
C17orf85	-1.5632	-3.7599	0.00016997	0.0038102	chr17
SNRPA	-1.2279	-3.7563	0.00017245	0.0038102	chr7
NR0B1	-2.6628	-3.755	0.00017336	0.0038102	chrX
SF3B4	-1.5626	-3.752	0.00017545	0.0038102	chr3
PRKAB2	-1.7617	-3.7462	0.00017951	0.0038102	chr3
TRNT1	-1.2317	-3.7403	0.00018377	0.0038102	chr6
YTHDF3	-1.2317	-3.7403	0.00018377	0.0038102	chr3
SYF2	-1.1431	-3.7381	0.00018545	0.0038102	chr4
GPKOW	-1.2027	-3.7376	0.00018582	0.0038102	chrX
DDX31	-1.9631	-3.736	0.00018698	0.0038102	chr2
MRPS5	-1.0468	-3.7354	0.00018744	0.0038102	chr2
MRPL48	-1.7819	-3.7343	0.0001882	0.0038102	chr7
LOC81691	-1.6983	-3.734	0.00018849	0.0038102	chr1
SRP9	-2.3054	-3.7333	0.00018902	0.0038102	chr1
RPLP0	-2.216	-3.7317	0.00019016	0.0038102	chr5
RBM18	-2.0187	-3.7311	0.00019067	0.0038102	chr2
QARS	-1.3845	-3.731	0.00019069	0.0038102	chr9
SCAF8	-1.259	-3.728	0.00019302	0.0038102	chr17
IFIT3	-1.0462	-3.7264	0.00019423	0.0038102	chr19
EIF4A2	-1.0793	-3.7257	0.00019479	0.0038102	chr16
PTBP1	-2.1952	-3.7238	0.00019622	0.0038102	chr10
UTP6	-1.142	-3.722	0.00019763	0.0038102	chr11
PAIP2	-0.93816	-3.7165	0.00020203	0.0038102	chr18
MRPL35	-1.9764	-3.7161	0.00020231	0.0038102	chr6
DDX51	-1.3823	-3.7156	0.00020271	0.0038102	chr5
GTPBP1	-1.9313	-3.7131	0.00020475	0.0038102	chr15
ANG	-2.0173	-3.7114	0.00020608	0.0038102	chr14
HNRNPH1	-2.6142	-3.7098	0.00020744	0.0038102	chr11
ZRSR2	-1.3121	-3.7082	0.00020872	0.0038102	chrX
RTCA	-1.2851	-3.7032	0.00021288	0.0038102	chr1
GARS	-1.8458	-3.7	0.00021564	0.0038102	chr6
ENOX2	-2.5185	-3.6979	0.00021739	0.0038102	chrX
CPEB4	-1.7926	-3.6977	0.00021752	0.0038102	chr11
SF1	-1.6425	-3.6947	0.00022019	0.0038102	chr19

TUBA1B	-1.5395	-3.6893	0.0002249	0.0038102	chr15
MRPL16	-1.3367	-3.6857	0.0002281	0.0038102	chr19
RBM17	-1.3365	-3.6834	0.00023011	0.0038102	chr2
TDRKH	-2.0305	-3.68	0.00023326	0.0038102	chr3
EIF4ENIF1	-2.0029	-3.6771	0.00023589	0.0038102	chr11
RAVER2	-2.3319	-3.6741	0.0002387	0.0038102	chr4
MRPL30	-1.9613	-3.671	0.00024165	0.0038102	chr1
SKIV2L	-1.842	-3.6682	0.00024422	0.0038102	chr17
DDX3Y	-1.4507	-3.6658	0.00024655	0.0038102	chrY
ZRSR1	-1.5176	-3.6631	0.00024913	0.0038102	chr11
PPARGC1A	-1.9603	-3.6616	0.00025061	0.0038102	chr5
MRPS31	-0.93748	-3.6605	0.00025176	0.0038102	chr8
EDC4	-0.93748	-3.6605	0.00025176	0.0038102	chr8
NONO	-0.9747	-3.6604	0.00025179	0.0038102	chrX
IPO8	-1.3857	-3.66	0.00025218	0.0038102	chr6
MRPL47	-1.6185	-3.6555	0.00025668	0.0038102	chr3
IGF2BP1	-0.89956	-3.6511	0.0002611	0.0038102	chr11
AGO2	-1.495	-3.6509	0.00026137	0.0038102	chr8
SNW1	-1.4095	-3.6499	0.00026235	0.0038102	chr12
IFIT1B	-1.4095	-3.6499	0.00026235	0.0038102	chr10
RPS19	-1.3351	-3.649	0.00026326	0.0038102	chr19
SYMPK	-2.0018	-3.647	0.00026537	0.0038102	chr7
LSM1	-2.6529	-3.6454	0.000267	0.0038102	chr8
KARS	-1.1079	-3.645	0.00026737	0.0038102	chr8
SAFB2	-0.86067	-3.6441	0.00026835	0.0038102	chr19
EPRS	-1.688	-3.6374	0.0002754	0.0038102	chr1
RPLP2	-1.656	-3.6369	0.00027597	0.0038102	chr7
EIF2AK2	-1.0746	-3.633	0.00028016	0.0038102	chr17
UHMK1	-2.214	-3.6323	0.00028088	0.0038102	chr1
NAA38	-1.9739	-3.6281	0.00028549	0.0038102	chr6
CCDC86	-1.3594	-3.6275	0.00028614	0.0038102	chr19
YTHDF1	-2.0542	-3.6272	0.00028648	0.0038102	chr2
APOBEC3F	-1.8444	-3.624	0.00029007	0.0038102	chr22
NSUN5	-2.0501	-3.6227	0.0002915	0.0038102	chr5
IARS2	-1.5394	-3.6217	0.00029264	0.0038102	chr1
TUBB	-1.6746	-3.6216	0.00029275	0.0038102	chr6
PSMC4	-1.3332	-3.6189	0.00029588	0.0038102	chr7
DAZ1	-2.0673	-3.6182	0.00029666	0.0038102	chrY
RNASE6	-1.9739	-3.6177	0.00029722	0.0038102	chr14
MRPS22	-1.2804	-3.6143	0.00030114	0.0038102	chr9
TLR7	-1.5362	-3.6123	0.00030344	0.0038102	chrX
ELAVL2	-1.6506	-3.6115	0.0003044	0.0038102	chr4
HNRNPM	-1.3834	-3.6087	0.00030777	0.0038102	chr17
NXF5	-1.3834	-3.6087	0.00030777	0.0038102	chrX
CCT7	-1.9361	-3.6084	0.00030806	0.0038102	chr6
VARS	-1.4073	-3.6055	0.00031158	0.0038102	chr17
DHX34	-2.2374	-3.6012	0.00031681	0.0038102	chr7
RPS11	-1.9188	-3.5998	0.00031842	0.0038102	chr7
RPL31	-0.81763	-3.5967	0.00032224	0.0038102	chr1
HNRNPLL	-2.6035	-3.5964	0.00032268	0.0038102	chr2
SPATS2L	-1.693	-3.5963	0.00032281	0.0038102	chr1

RNMT	-2.3277	-3.5955	0.00032381	0.0038102	chr18
RNF17	-1.9227	-3.5905	0.00033	0.0038102	chr14
TARBP2	-1.5969	-3.5884	0.00033273	0.0038102	chr15
MRPS28	-2.0122	-3.5864	0.00033534	0.0038102	chr15
DHX30	-1.6733	-3.5841	0.00033828	0.0038102	chr9
TIA1	-1.4652	-3.5839	0.00033851	0.0038102	chr6
PSMD1	-1.4988	-3.5834	0.00033911	0.0038102	chr1
IGF2BP2	-1.041	-3.5824	0.00034049	0.0038102	chr16
DDX25	-2.588	-3.5823	0.00034054	0.0038102	chr9
PPIE	-1.728	-3.5819	0.00034109	0.0038102	chr4
MRPL21	-1.5968	-3.5808	0.00034257	0.0038102	chr19
RPL10A	-1.3311	-3.5732	0.0003526	0.0038102	chr6
HARS2	-1.8292	-3.5713	0.00035527	0.0038102	chr18
PCBP4	-1.6155	-3.5702	0.00035673	0.0038102	chr9
DNAJC17	-2.1315	-3.57	0.00035694	0.0038102	chr6
RNASE2	-1.4524	-3.57	0.00035699	0.0038102	chr14
LSM14B	-2.0651	-3.5686	0.00035886	0.0038102	chr2
MOV10L1	-1.7262	-3.5676	0.00036022	0.0038102	chr15
LARS	-1.8452	-3.5663	0.00036208	0.0038102	chr18
RBPMS	-2.6923	-3.5656	0.00036301	0.0038102	chr8
MRPL17	-2.0525	-3.5634	0.00036604	0.0038102	chr7
RBM3	-2.5474	-3.5595	0.00037151	0.0038102	chrX
EIF4G1	-1.4749	-3.5591	0.00037218	0.0038102	chr16
RRP12	-1.4047	-3.5575	0.00037441	0.0038102	chr16
SF3B2	-1.4047	-3.5575	0.00037441	0.0038102	chr19
RBM48	-1.4054	-3.5574	0.0003745	0.0038102	chr7
SF3A1	-1.4054	-3.5574	0.0003745	0.0038102	chr11
SRRM3	-1.7787	-3.5567	0.00037551	0.0038102	chr5
RBM15	-2.025	-3.5556	0.00037717	0.0038102	chr3
DKC1	-1.3562	-3.5555	0.00037732	0.0038102	chrX
EIF2AK3	-1.7096	-3.5543	0.000379	0.0038102	chr6
SART3	-1.1664	-3.5543	0.00037897	0.0038102	chr5
NHP2	-1.7619	-3.5541	0.00037921	0.0038102	chr11
RBM20	-1.6903	-3.5541	0.00037931	0.0038102	chr10
MOV10	-1.4045	-3.5541	0.00037925	0.0038102	chr3
ADAT2	-1.8516	-3.5533	0.00038039	0.0038102	chr10
RPA2	-2.1082	-3.5526	0.00038144	0.0038102	chr4
NKRF	-1.6176	-3.5525	0.0003816	0.0038102	chrX
STRBP	-1.0338	-3.5525	0.00038164	0.0038102	chr2
CNOT11	-1.2784	-3.5522	0.00038206	0.0038102	chr2
PABPC5	-2.5914	-3.5497	0.00038573	0.0038102	chrX
MRPS35	-2.1146	-3.5486	0.00038732	0.0038102	chr6
PSMA3	-1.7787	-3.5481	0.00038808	0.0038102	chr12
RPS27A	-1.8408	-3.5456	0.00039168	0.0038102	chr2
SNRPB2	-1.4741	-3.5411	0.00039849	0.0038102	chr2
RAVER1	-2.6817	-3.5406	0.00039927	0.0038102	chr9
LARP4B	-1.4927	-3.5388	0.00040198	0.0038102	chr13
RPL36AL	-1.4963	-3.5385	0.00040237	0.0038102	chr14
AUH	-2.2848	-3.5381	0.00040304	0.0038102	chr13
EIF1AD	-1.9436	-3.5339	0.0004094	0.0038102	chr19
EARS2	-2.05	-3.5338	0.0004097	0.0038102	chr7

MRPL40	-1.7094	-3.5335	0.00041003	0.0038102	chr16
ZFC3H1	-2.4122	-3.5308	0.00041429	0.0038102	chr10
FBXO17	-1.2488	-3.5281	0.00041853	0.0038102	chr7
GPATCH1	-1.9964	-3.5275	0.00041958	0.0038102	chr7
EEF1D	-1.9156	-3.5265	0.00042116	0.0038102	chr8
CSTF3	-0.72879	-3.5242	0.00042477	0.0038102	chr2
RBMXL2	-2.1637	-3.5241	0.00042493	0.0038102	chr7
MRPL52	-2.07	-3.5232	0.00042642	0.0038102	chr14
EIF3CL	-1.674	-3.5225	0.00042748	0.0038102	chr16
EIF1AX	-1.8105	-3.5219	0.00042851	0.0038102	chr5
GPATCH8	-1.761	-3.5215	0.00042917	0.0038102	chr11
HEATR1	-1.552	-3.5212	0.00042957	0.0038102	chr13
EIF3I	-1.5184	-3.5209	0.00043007	0.0038102	chr4
GTPBP2	-2.4284	-3.5169	0.00043656	0.0038102	chr17
RPS25	-1.5172	-3.5158	0.00043843	0.0038102	chr9
RBM23	-1.5172	-3.5158	0.00043843	0.0038102	chr14
DDX17	-2.3648	-3.5151	0.00043959	0.0038102	chr15
SND1	-2.3357	-3.5148	0.00044013	0.0038102	chr6
SFPQ	-2.0272	-3.5131	0.00044299	0.0038102	chr4
RBMS2	-0.77322	-3.5112	0.00044609	0.0038102	chr12
MRPL32	-1.5126	-3.5104	0.00044744	0.0038102	chr13
DGCR8	-2.0582	-3.5088	0.00045011	0.0038102	chr16
ZFP36L1	-1.4945	-3.5052	0.00045629	0.0038102	chr12
EIF1B	-2.1088	-3.5048	0.00045688	0.0038102	chr9
MRPS17	-2.2884	-3.5044	0.00045758	0.0038102	chr5
IFIT2	-2.0763	-3.503	0.00046005	0.0038102	chr19
KHDRBS2	-2.2519	-3.5022	0.00046149	0.0038102	chr1
SETD1B	-1.2501	-3.5021	0.00046165	0.0038102	chr12
TNRC6A	-1.004	-3.5009	0.00046375	0.0038102	chr7
ZGPAT	-1.8418	-3.4995	0.00046615	0.0038102	chr2
TNRC6C	-1.9581	-3.4974	0.00046978	0.0038102	chr4
MRPL3	-1.1327	-3.4964	0.00047163	0.0038102	chr9
NSUN2	-1.1327	-3.4964	0.00047163	0.0038102	chr5
RBM47	-1.273	-3.4956	0.00047294	0.0038102	chr1
CNOT7	-1.8734	-3.4954	0.00047327	0.0038102	chr8
MBNL1	-1.7233	-3.4927	0.00047818	0.0038102	chr3
TLR3	-1.5791	-3.49	0.00048308	0.0038102	chr8
TSEN2	-1.5155	-3.4889	0.00048504	0.0038102	chr6
RBFOX3	-1.0067	-3.4881	0.00048643	0.0038102	chr11
IPO7	-1.0409	-3.4874	0.00048767	0.0038102	chr7
PUS3	-0.97152	-3.4857	0.00049078	0.0038102	chr9
METTL1	-1.4936	-3.4848	0.00049252	0.0038102	chr10
TNPO1	-0.97109	-3.4828	0.00049625	0.0038102	chr13
RBM43	-0.97109	-3.4828	0.00049625	0.0038102	chr2
DHX15	-0.97109	-3.4828	0.00049625	0.0038102	chr5
MRPL9	-0.92965	-3.4813	0.00049902	0.0038102	chr3
RNASE1	-1.6139	-3.4811	0.00049937	0.0038102	chr14
DARS2	-1.5992	-3.4799	0.00050158	0.0038102	chr1
MRPL44	-0.93465	-3.4786	0.00050413	0.0038102	chr1
EXOSC2	-1.1917	-3.4781	0.00050495	0.0038102	chr2
ICK	-1.1055	-3.476	0.00050895	0.0038102	chr9

ALKBH8	-1.8782	-3.4743	0.00051221	0.0038102	chr9
MRPL39	-1.0397	-3.473	0.00051461	0.0038102	chr16
TDRD15	-1.0739	-3.4728	0.00051505	0.0038102	chr2
RBFOX2	-1.2789	-3.4725	0.00051563	0.0038102	chr15
RBMY1J	-2.4713	-3.4711	0.00051829	0.0038102	chrY
RPTOR	-0.76999	-3.4702	0.00052006	0.0038102	chr11
TFIP11	-1.4427	-3.4681	0.00052414	0.0038102	chr5
RPS9	-1.5365	-3.4675	0.00052532	0.0038102	chr7
NCBP2	-1.5146	-3.4639	0.00053243	0.0038102	chr1
RNASE12	-1.5982	-3.4634	0.00053339	0.0038102	chr14
AAR2	-1.8404	-3.4631	0.00053399	0.0038102	chr20
PNLDC1	-1.6703	-3.4618	0.00053661	0.0038102	chr17
RPL6	-1.4662	-3.4615	0.00053715	0.0038102	chr5
ZNF638	-1.9176	-3.4584	0.0005434	0.0038102	chr2
FMR1	-1.5495	-3.4578	0.00054456	0.0038102	chrX
TRMT2A	-2.1595	-3.4577	0.0005448	0.0038102	chr22
RNASE13	-2.0855	-3.4561	0.00054812	0.0038102	chr14
RPS20	-1.3993	-3.4545	0.00055129	0.0038102	chr4
RBM45	-1.166	-3.4544	0.00055155	0.0038102	chr2
RBM28	-0.93282	-3.4542	0.00055199	0.0038102	chr6
DDX23	-1.47	-3.4524	0.00055565	0.0038102	chr15
DDX54	-1.1656	-3.452	0.00055641	0.0038102	chr5
CSTF2T	-0.8952	-3.452	0.00055637	0.0038102	chr19
ATXN1L	-1.8161	-3.4519	0.00055666	0.0038102	chr8
CWC27	-1.7397	-3.4507	0.00055905	0.0038102	chr13
RNPC3	-0.85663	-3.45	0.00056067	0.0038102	chr3
PABPC1L	-1.6175	-3.4493	0.00056206	0.0038102	chr2
CSTF1	-1.4234	-3.4488	0.00056303	0.0038102	chr2
RUVBL2	-1.9421	-3.4484	0.00056401	0.0038102	chr7
ADAR	-1.5132	-3.447	0.00056682	0.0038102	chr3
EEFSEC	-1.3531	-3.4466	0.00056776	0.0038102	chr6
CLK4	-1.9318	-3.4456	0.00056987	0.0038102	chr5
AARS	-1.1346	-3.4456	0.00056983	0.0038102	chr8
ALYREF	-2.0629	-3.4437	0.00057377	0.0038102	chr11
EIF5	-1.1649	-3.4422	0.00057701	0.0038102	chr12
DCP1B	-2.0715	-3.4419	0.00057756	0.0038102	chr6
NOVA1	-1.9561	-3.4416	0.00057828	0.0038102	chr12
RBMY1F	-1.8525	-3.4406	0.00058044	0.0038102	chrY
MRPS34	-1.1947	-3.4403	0.00058106	0.0038102	chr17
RPL39L	-1.1652	-3.4395	0.00058272	0.0038102	chr3
XPO7	-2.2237	-3.4391	0.00058362	0.0038102	chr14
APOBEC2	-1.1036	-3.4375	0.00058701	0.0038195	chr17
FXR2	-2.2176	-3.4364	0.00058955	0.0038232	chr11
EIF2S2	-1.5133	-3.4352	0.00059221	0.0038276	chr2
RPL28	-0.93123	-3.429	0.00060587	0.003876	chr7
EXOSC8	-1.0369	-3.4263	0.00061195	0.003876	chr3
CNOT2	-1.0369	-3.4263	0.00061195	0.003876	chr12
ERI2	-1.1639	-3.4253	0.00061414	0.003876	chr7
LSM2	-1.2228	-3.4249	0.00061496	0.003876	chr6
TARS2	-1.1334	-3.4245	0.000616	0.003876	chr3
HNRNPF	-1.1334	-3.4245	0.000616	0.003876	chr6

CPSF4L	-0.97255	-3.4239	0.00061718	0.003876	chr11
DHX36	-1.2224	-3.4228	0.00061968	0.003876	chr3
CPSF3L	-1.2224	-3.4228	0.00061968	0.003876	chr4
MATR3	-1.1933	-3.4217	0.00062238	0.0038803	chr18
PRPF31	-1.8233	-3.4192	0.00062799	0.0039028	chr7
RRP1B	-2.6313	-3.4166	0.00063406	0.0039237	chr17
ZFP36	-2.1298	-3.416	0.00063539	0.0039237	chr7
CSTF2	-1.3684	-3.415	0.00063793	0.0039268	chrX
PIWIL1	-1.9782	-3.413	0.00064241	0.0039289	chr3
MRPS11	-0.9269	-3.4125	0.00064375	0.0039289	chr15
UTP23	-1.376	-3.4118	0.00064526	0.0039289	chr15
GAPDH	-1.995	-3.4114	0.00064637	0.0039289	chr6
RSL24D1	-1.6349	-3.4092	0.00065143	0.0039381	chr9
PUM2	-1.0016	-3.4088	0.0006524	0.0039381	chr12
RPN2	-1.9448	-3.4082	0.00065398	0.0039381	chr2
RBM11	-1.7054	-3.407	0.00065687	0.0039432	chr16
ZNF622	-1.6345	-3.4044	0.00066305	0.0039681	chr5
SSB	-1.1625	-3.4018	0.00066938	0.0039756	chr2
PAPOLG	-1.4	-3.4012	0.0006709	0.0039756	chr11
THUMPD1	-1.8846	-3.4009	0.00067166	0.0039756	chr7
RSL1D1	-0.89188	-3.4005	0.00067252	0.0039756	chr16
TRNAU1AP	-2.2034	-3.3969	0.00068163	0.0040051	chr4
EIF4B	-1.1317	-3.3969	0.00068153	0.0040051	chr15
PHB	-0.92917	-3.3952	0.00068582	0.0040175	chr11
MRPL53	-1.5985	-3.3936	0.00068978	0.0040286	chr6
SUPT5H	-1.9265	-3.3909	0.00069658	0.0040355	chr7
FARSA	-1.2485	-3.3909	0.00069672	0.0040355	chr8
CD2BP2	-1.5744	-3.3907	0.00069721	0.0040355	chr7
EIF6	-1.5941	-3.3858	0.00070978	0.0040366	chr2
SUGP2	-2.3526	-3.3857	0.00071003	0.0040366	chr8
QKI	-1.7751	-3.3852	0.00071125	0.0040366	chr6
PUS10	-0.96495	-3.3846	0.00071284	0.0040366	chr11
EXOSC3	-0.96495	-3.3846	0.00071284	0.0040366	chr4
DDX5	-0.96495	-3.3846	0.00071284	0.0040366	chr11
EXOSC1	-2.0582	-3.3834	0.00071587	0.0040366	chr19
HNRNPK	-1.6139	-3.3834	0.00071593	0.0040366	chr13
PUS7L	-2.0493	-3.3833	0.00071615	0.0040366	chr15
PARP1	-1.5278	-3.382	0.0007197	0.0040449	chr1
RBM10	-1.2754	-3.3797	0.00072575	0.0040491	chrX
TARBP1	-0.89318	-3.3794	0.00072657	0.0040491	chr1
TUT1	-1.8832	-3.3793	0.0007267	0.0040491	chr19
WDR5	-1.2195	-3.377	0.00073286	0.0040717	chr2
RNASE3	-2.2249	-3.3752	0.00073771	0.0040765	chr14
RPL13	-1.2474	-3.3732	0.00074309	0.0040765	chr8
RBM42	-1.6903	-3.3729	0.00074394	0.0040765	chr7
RPL14	-1.0668	-3.3723	0.00074533	0.0040765	chr9
CDC5L	-1.9475	-3.371	0.00074891	0.0040765	chr17
CMTR1	-2.1904	-3.3705	0.0007502	0.0040765	chr6
RNASET2	-2.0703	-3.3701	0.00075132	0.0040765	chr3
PRPF3	-0.99931	-3.3696	0.00075265	0.0040765	chr3
METTL10	-0.99931	-3.3696	0.00075265	0.0040765	chr7

DDX4	-1.5631	-3.367	0.00075989	0.0040926	chr13
SARS	-1.8669	-3.3664	0.00076158	0.0040926	chr3
CPSF4	-1.2745	-3.3657	0.00076362	0.0040926	chr5
TDRD6	-1.6712	-3.365	0.00076536	0.0040926	chr17
MRPS14	-1.5933	-3.3647	0.00076618	0.0040926	chr1
RPL3L	-2.3963	-3.3633	0.00077021	0.0040947	chr17
ESRP2	-1.3699	-3.3631	0.0007708	0.0040947	chr8
RBM5	-1.8822	-3.3609	0.00077691	0.0041064	chr9
MRPL50	-1.9274	-3.36	0.00077931	0.0041064	chr4
CWC15	-1.9281	-3.36	0.00077935	0.0041064	chr9
NUP93	-1.6319	-3.3566	0.0007892	0.0041156	chr8
RINT1	-1.4877	-3.3518	0.00080285	0.0041156	chr5
CELF1	-1.6433	-3.3508	0.00080569	0.0041156	chr2
IPO11	-1.7077	-3.3505	0.00080673	0.0041156	chr13
ASCC1	-1.9556	-3.35	0.00080822	0.0041156	chr10
SRP68	-2.8504	-3.3498	0.00080872	0.0041156	chr11
U2AF2	-1.5101	-3.3498	0.00080884	0.0041156	chr7
PABPC1	-1.0976	-3.3496	0.00080921	0.0041156	chr15
STAU2	-1.3269	-3.3495	0.00080956	0.0041156	chr1
RPL7A	-1.3269	-3.3495	0.00080956	0.0041156	chr2
IPO4	-2.5939	-3.349	0.00081096	0.0041156	chr14
RAE1	-1.8661	-3.3481	0.00081362	0.0041156	chr2
FTO	-2.016	-3.3471	0.00081668	0.0041156	chr8
RNPS1	-0.72456	-3.3469	0.00081715	0.0041156	chr17
YARS	-0.72456	-3.3469	0.00081715	0.0041156	chr4
RRM1	-0.72456	-3.3469	0.00081715	0.0041156	chr11
RNASEH1	-0.96035	-3.3463	0.0008189	0.0041156	chr12
MRPS25	-1.9267	-3.3462	0.00081929	0.0041156	chr6
RPL35A	-1.8353	-3.3431	0.00082854	0.0041513	chr16
DAZAP1	-1.9786	-3.3415	0.00083329	0.0041643	chr10
RPL18	-1.6119	-3.3385	0.00084229	0.0041886	chr7
RBM44	-2.2236	-3.3384	0.00084258	0.0041886	chr1
YTHDC1	-1.6117	-3.3377	0.00084464	0.0041886	chr5
LARP4	-1.9408	-3.3349	0.00085343	0.0042214	chr15
YBX2	-1.7635	-3.3333	0.00085823	0.0042344	chr11
NUDT21	-1.6692	-3.3312	0.00086466	0.004248	chr8
EIF3K	-1.6877	-3.331	0.00086538	0.004248	chr7
CELF4	-1.6304	-3.3293	0.0008705	0.0042543	chr18
NANOS2	-1.3512	-3.3282	0.0008742	0.0042543	chr7
DDX59	-1.9836	-3.3281	0.00087426	0.0042543	chr1
EIF4E3	-1.1537	-3.3278	0.00087544	0.0042543	chr6
RPL27	-1.7063	-3.3263	0.0008802	0.0042666	chr11
BICC1	-1.6103	-3.3246	0.00088533	0.0042666	chr10
RPS7	-1.3508	-3.324	0.00088725	0.0042666	chr12
APOBEC3H	-2.2118	-3.3235	0.00088893	0.0042666	chr22
FARSB	-0.72288	-3.3235	0.00088897	0.0042666	chr1
CNOT4	-1.803	-3.3217	0.00089471	0.0042835	chr6
EIF5A2	-0.99656	-3.32	0.00090018	0.0042886	chr3
SNRPB	-0.99656	-3.32	0.00090018	0.0042886	chr2
SF3B5	-1.4654	-3.3192	0.00090279	0.0042905	chr10
PTRH1	-2.2127	-3.3173	0.00090902	0.0043095	chr5

RPL26L1	-1.9391	-3.3162	0.00091251	0.0043155	chr5
MRPS6	-1.4854	-3.3152	0.00091572	0.0043201	chr16
FAM98C	-1.5293	-3.3144	0.0009184	0.0043205	chr3
CNOT10	-1.8159	-3.3136	0.00092102	0.0043205	chr9
MTPAP	-0.67818	-3.3129	0.00092331	0.0043205	chr18
ADARB1	-1.8968	-3.3115	0.00092806	0.0043205	chr21
HNRNPR	-1.3241	-3.3103	0.00093212	0.0043205	chr4
PSMA1	-1.3241	-3.3103	0.00093212	0.0043205	chr7
DAZ2	-1.1865	-3.3101	0.00093247	0.0043205	chrY
ZNFX1	-0.88202	-3.3098	0.00093362	0.0043205	chr2
PAIP1	-0.67847	-3.3091	0.00093604	0.0043214	chr13
CDK17	-2.3787	-3.3068	0.0009438	0.004335	chr10
PRPF39	-1.375	-3.3062	0.00094577	0.004335	chr14
DDX19A	-1.8349	-3.3061	0.00094604	0.004335	chr8
RNASEH2A	-1.5054	-3.3055	0.00094806	0.004335	chr8
G3BP2	-1.7184	-3.3049	0.00095016	0.004335	chr5
RRP15	-1.6293	-3.3037	0.00095432	0.0043364	chr1
RBM8A	-1.6433	-3.3035	0.00095494	0.0043364	chr3
DCP2	-1.7045	-3.3017	0.000961	0.0043432	chr20
CPSF1	-1.2927	-3.3011	0.00096313	0.0043432	chr15
DDX20	-2.2694	-3.3009	0.00096391	0.0043432	chr3
INTS6	-1.0295	-3.2999	0.00096733	0.0043432	chr14
FASTKD3	-0.80753	-3.2991	0.00097002	0.0043432	chr13
DDX21	-1.55	-3.2986	0.00097162	0.0043432	chr10
EIF4E1B	-1.3742	-3.2984	0.00097227	0.0043432	chr13
RPS2	-1.5498	-3.2978	0.00097436	0.0043432	chr16
CCT4	-1.7049	-3.2971	0.00097687	0.0043444	chr11
EXOSC6	-2.0237	-3.2963	0.00097955	0.0043459	chr16
RBMY1D	-2.1049	-3.2957	0.00098168	0.0043459	chrY
DIS3	-0.88664	-3.2938	0.00098852	0.0043521	chr14
RPS28	-1.414	-3.2937	0.00098884	0.0043521	chrX
ELAVL3	-2.177	-3.2934	0.00098984	0.0043521	chr9
LARS2	-1.2699	-3.2917	0.00099587	0.0043649	chr9
PRPF4	-0.76515	-3.2912	0.00099751	0.0043649	chr4
DDX26B	-1.8594	-3.2907	0.00099949	0.0043649	chrX
SRP14	-1.7219	-3.29	0.0010019	0.0043655	chr2
EXOSC5	-1.2422	-3.2884	0.0010076	0.0043727	chr7
EIF5B	-1.7222	-3.2877	0.0010101	0.0043727	chr1
PRPF4B	-1.834	-3.2876	0.0010103	0.0043727	chr13
SRRM2	-2.058	-3.2862	0.0010154	0.0043852	chr17
SF3A3	-1.398	-3.2836	0.001025	0.0044068	chr4
ENOX1	-1.398	-3.2836	0.001025	0.0044068	chr14
EIF3J	-1.7033	-3.2812	0.0010336	0.0044176	chr2
TGS1	-1.3479	-3.2808	0.0010353	0.0044176	chr4
APOBEC3G	-2.1605	-3.2804	0.0010367	0.0044176	chr22
ISY1	-1.1246	-3.2798	0.0010389	0.0044176	chr6
XPO6	-1.7571	-3.2795	0.0010399	0.0044176	chr7
DHX57	-1.2374	-3.2792	0.0010412	0.0044176	chr17
INTS9	-2.358	-3.2784	0.0010441	0.0044203	chr14
RBM7	-2.0363	-3.2773	0.001048	0.0044205	chr9
RARS	-1.0613	-3.2771	0.0010487	0.0044205	chr2

SMN2	-2.1082	-3.2745	0.0010585	0.0044397	chr5
CWC22	-0.72064	-3.2744	0.0010587	0.0044397	chr2
PSMD6	-1.8489	-3.2741	0.0010601	0.0044397	chr14
IGF2BP3	-1.8162	-3.2717	0.001069	0.0044606	chr6
GEMIN5	-2.0847	-3.2714	0.0010702	0.0044606	chr11
EWSR1	-1.3723	-3.2709	0.001072	0.0044606	chr11
ADAD1	-1.184	-3.2696	0.001077	0.0044632	chr3
EEF1G	-1.8164	-3.2695	0.0010772	0.0044632	chr19
DHX32	-0.95923	-3.268	0.0010832	0.0044782	chr6
EIF4E2	-1.5524	-3.2669	0.0010875	0.0044865	chr19
C12orf65	-1.3257	-3.2653	0.0010936	0.0045023	chr15
CIRBP	-1.7192	-3.2624	0.0011046	0.0045376	chr10
UTP20	-1.3713	-3.2615	0.0011082	0.0045428	chr10
DIS3L	-1.4207	-3.2604	0.0011124	0.0045448	chr9
SRRM4	-2.0352	-3.2602	0.0011133	0.0045448	chr5
CELF5	-2.2209	-3.2592	0.0011172	0.0045511	chr10
PARN	-1.3961	-3.2584	0.0011206	0.004553	chr16
SRSF7	-2.127	-3.2574	0.0011245	0.004553	chr17
SNRNP25	-1.3463	-3.2569	0.0011263	0.004553	chr11
RNF113B	-2.2078	-3.2567	0.0011271	0.004553	chr13
XPO1	-0.62741	-3.2555	0.0011318	0.004553	chr11
POLR2D	-0.62741	-3.2555	0.0011318	0.004553	chr18
BOLL	-2.4641	-3.2543	0.0011366	0.0045563	chr1
DARS	-1.1533	-3.2523	0.0011447	0.0045563	chr1
LARP1B	-1.0919	-3.2523	0.0011448	0.0045563	chr3
RALY	-1.9662	-3.252	0.0011458	0.0045563	chr2
RNMTL1	-1.7376	-3.2518	0.0011467	0.0045563	chr11
DDX42	-0.99338	-3.2496	0.0011558	0.0045829	chr1
AQR	-1.205	-3.2484	0.0011606	0.0045836	chr2
FASTKD2	-2.3502	-3.2476	0.0011637	0.0045836	chr1
U2AF1	-2.2077	-3.247	0.0011664	0.0045836	chr17
LUC7L	-0.6738	-3.247	0.0011664	0.0045836	chr17
TLR8	-2.3857	-3.2466	0.0011678	0.0045836	chrX
PIH1D2	-0.84407	-3.2418	0.0011878	0.0046526	chr9
PARK7	-1.963	-3.2411	0.0011906	0.0046545	chr4
CNOT3	-1.211	-3.2402	0.0011945	0.0046603	chr19
INTS3	-1.9092	-3.2382	0.001203	0.0046839	chr3
PABPC4	-1.4426	-3.2369	0.0012081	0.0046946	chr4
IMP3	-1.2929	-3.2347	0.0012176	0.004722	chr9
CLK2	-1.4835	-3.2314	0.0012319	0.0047603	chr3
MAEL	-2.2941	-3.2313	0.0012324	0.0047603	chr1
A1CF	-1.5479	-3.2298	0.0012388	0.0047648	chr19
TRA2A	-1.4108	-3.2291	0.001242	0.0047648	chr6
EIF2AK4	-2.2943	-3.2288	0.001243	0.0047648	chr2
WARS	-1.4182	-3.2287	0.0012434	0.0047648	chr12
EIF3H	-1.7367	-3.228	0.0012468	0.0047683	chr15
RPS15A	-2.3041	-3.2258	0.0012562	0.0047949	chr14
EFTUD2	-1.7882	-3.2247	0.0012608	0.0048031	chr11
PABPC1L2B	-2.1471	-3.2238	0.0012652	0.0048101	chrX
CMSS1	-1.766	-3.2226	0.0012704	0.0048186	chr3
MTHFSD	-2.4195	-3.2221	0.0012724	0.0048186	chr17

UTP14A	-1.3935	-3.2191	0.0012861	0.0048611	chrX
INTS8	-1.8035	-3.2182	0.0012899	0.004866	chr4
TDRD12	-1.7533	-3.2171	0.0012952	0.0048669	chr7
RBMX	-1.7533	-3.2171	0.0012952	0.0048669	chrX
IFIT5	-1.6041	-3.2158	0.0013007	0.0048784	chr10
MBNL3	-2.0725	-3.2142	0.0013081	0.0048964	chrX
RPS26	-1.4643	-3.213	0.0013135	0.004897	chr12
GEMIN6	-1.4643	-3.213	0.0013135	0.004897	chr2
ERI1	-2.5136	-3.2119	0.0013184	0.0048974	chr8
MRPL22	-1.873	-3.2102	0.0013263	0.0049173	chr11
CASC3	-2.2645	-3.2096	0.0013293	0.0049188	chr11
DDX53	-2.2822	-3.2062	0.001345	0.0049675	chrX
RAN	-1.0579	-3.2049	0.001351	0.0049707	chr5
FAU	-1.0579	-3.2049	0.001351	0.0049707	chr19
SETD1A	-1.6799	-3.2001	0.001374	0.0050457	chr7
SNRPA1	-1.4397	-3.1984	0.0013821	0.0050658	chr7
MRPL10	-1.3424	-3.1975	0.0013863	0.0050716	chr11
DDX56	-1.7696	-3.1955	0.0013958	0.0050759	chr11
RBM24	-1.3166	-3.1949	0.0013988	0.0050759	chr13
CDC40	-1.3918	-3.1935	0.0014054	0.0050759	chr10
RPL30	-1.3918	-3.1935	0.0014054	0.0050759	chr8
LIN28B	-0.89562	-3.1932	0.0014069	0.0050759	chr10
RALYL	-1.8576	-3.1931	0.0014076	0.0050759	chr3
SNRNP35	-1.6032	-3.1931	0.0014076	0.0050759	chr5
TNRC6B	-2.638	-3.1926	0.0014099	0.0050759	chr15
MRPL13	-2.0582	-3.1921	0.0014125	0.0050759	chr15
PTRHD1	-2.165	-3.1902	0.0014217	0.0050759	chr2
PSMD7	-1.7337	-3.1886	0.0014295	0.0050759	chr8
HDLBP	-2.3998	-3.1885	0.0014301	0.0050759	chr1
PCBP1	-1.919	-3.186	0.0014425	0.0050759	chr6
XPOT	-1.8026	-3.1854	0.0014457	0.0050759	chr12
PIWIL3	-2.0262	-3.1846	0.0014494	0.0050759	chr22
PAIP2B	-1.6899	-3.1838	0.0014538	0.0050759	chr6
MRPL34	-0.57365	-3.1833	0.0014563	0.0050759	chr8
HNRNPL	-1.8934	-3.1831	0.001457	0.0050759	chr7
PSMA2	-1.8195	-3.1829	0.0014582	0.0050759	chr13
ACO1	-1.912	-3.1804	0.0014707	0.0050759	chr4
SMN1	-2.2372	-3.1797	0.0014743	0.0050759	chr13
PURG	-1.4843	-3.1794	0.0014759	0.0050759	chr8
EIF5AL1	-1.8311	-3.179	0.0014776	0.0050759	chr10
PINX1	-1.2625	-3.1789	0.0014784	0.0050759	chr14
LRRC47	-1.4384	-3.1786	0.0014799	0.0050759	chr4
ZCCHC24	-1.4384	-3.1786	0.0014799	0.0050759	chr14
SMNDC1	-1.6969	-3.1785	0.0014804	0.0050759	chr19
NSUN7	-0.80444	-3.1772	0.0014868	0.0050759	chr5
NSUN3	-0.80444	-3.1772	0.0014868	0.0050759	chr5
RPS16	-0.80444	-3.1772	0.0014868	0.0050759	chr7
RNASE9	-0.80444	-3.1772	0.0014868	0.0050759	chr14
DICER1	-0.80444	-3.1772	0.0014868	0.0050759	chr12
RNASE7	-0.80444	-3.1772	0.0014868	0.0050759	chr14
RPL3	-0.80444	-3.1772	0.0014868	0.0050759	chr15

RPL5	-0.84476	-3.1767	0.0014895	0.0050759	chr5
EIF2AK1	-0.84476	-3.1767	0.0014895	0.0050759	chr7
U2AF1L4	-0.84476	-3.1767	0.0014895	0.0050759	chr7
XRN2	-0.84476	-3.1767	0.0014895	0.0050759	chr22
RPS4X	-0.84476	-3.1767	0.0014895	0.0050759	chrX
RPS17	-1.8156	-3.1758	0.0014942	0.005083	chr15
NAF1	-2.0325	-3.172	0.0015141	0.005098	chr8
MRPS21	-1.8352	-3.1716	0.0015161	0.005098	chr3
PUF60	-0.7622	-3.1707	0.0015209	0.005098	chr15
RPS23	-0.7622	-3.1707	0.0015209	0.005098	chr5
ZCCHC5	-0.7622	-3.1707	0.0015209	0.005098	chrX
KRR1	-0.88332	-3.1704	0.0015223	0.005098	chr12
BRCA1	-0.88332	-3.1704	0.0015223	0.005098	chr11
DDX52	-0.88332	-3.1704	0.0015223	0.005098	chr11
EIF3A	-0.88332	-3.1704	0.0015223	0.005098	chr19
LARP1	-1.4607	-3.1675	0.0015377	0.00513	chr11
RFC4	-1.4607	-3.1675	0.0015377	0.00513	chr16
INTS10	-1.8588	-3.1671	0.0015398	0.00513	chr8
MRPL37	-2.1083	-3.1659	0.0015459	0.0051415	chr4
SNRNP27	-2.3477	-3.1652	0.0015499	0.0051461	chr6
SAFB	-0.76599	-3.1642	0.0015552	0.0051549	chr17
RNASE11	-1.732	-3.1624	0.001565	0.0051706	chr14
SON	-1.6338	-3.1611	0.0015718	0.0051706	chr16
RPL9	-0.92026	-3.1593	0.0015813	0.0051706	chr4
EFTUD1	-0.92026	-3.1593	0.0015813	0.0051706	chr7
MAK16	-0.92026	-3.1593	0.0015813	0.0051706	chr8
EIF3E	-0.92026	-3.1593	0.0015813	0.0051706	chr15
PTRH2	-0.92026	-3.1593	0.0015813	0.0051706	chr11
UTP15	-0.71785	-3.1555	0.0016024	0.0051967	chr5
TAF2	-0.71785	-3.1555	0.0016024	0.0051967	chr15
DDX27	-0.71785	-3.1555	0.0016024	0.0051967	chr2
EEF1B2	-0.71785	-3.1555	0.0016024	0.0051967	chr1
SLIRP	-1.4368	-3.1553	0.0016035	0.0051967	chr14
SERBP1	-1.8861	-3.1549	0.0016054	0.0051967	chr6
RBM41	-1.4338	-3.1536	0.0016127	0.0052001	chrX
MRPL33	-1.8502	-3.1532	0.0016147	0.0052001	chr2
DDX41	-1.6046	-3.1528	0.0016173	0.0052001	chr13
LSM5	-1.8502	-3.1519	0.0016223	0.0052001	chr6
EIF3M	-0.57206	-3.1517	0.0016231	0.0052001	chr2
DDX43	-2.1262	-3.1513	0.0016252	0.0052001	chr9
METTL14	-2.0291	-3.1502	0.0016314	0.0052049	chr3
CNOT1	-2.2903	-3.1501	0.0016321	0.0052049	chr8
PRPF19	-1.1543	-3.1496	0.0016348	0.0052051	chr19
IPO5	-1.639	-3.1482	0.0016429	0.0052222	chr14
RBM6	-1.8167	-3.1457	0.001657	0.0052476	chr9
DNMT1	-0.76262	-3.1448	0.0016623	0.0052476	chr9
EXO1	-0.9557	-3.1444	0.0016644	0.0052476	chr1
SPATS2	-0.9557	-3.1444	0.0016644	0.0052476	chr15
COPS2	-0.9557	-3.1444	0.0016644	0.0052476	chr2
RPL23	-1.9	-3.142	0.0016778	0.0052813	chr11
LSM6	-1.526	-3.1403	0.0016879	0.0052885	chr12

THOC1	-1.526	-3.1403	0.0016879	0.0052885	chr18
LARP7	-2.0888	-3.1402	0.0016883	0.0052885	chr3
RBMXL3	-2.4539	-3.1396	0.001692	0.0052917	chrX
UTP18	-1.5037	-3.1389	0.0016957	0.0052945	chr11
EIF1	-2.042	-3.1378	0.001702	0.0053058	chr11
DHX33	-1.6757	-3.137	0.0017067	0.0053092	chr11
MTRF1	-2.0653	-3.1367	0.0017086	0.0053092	chr14
PNPT1	-2.0686	-3.1359	0.0017134	0.0053144	chr11
MAZ	-2.3034	-3.1354	0.0017159	0.0053144	chr7
WARS2	-1.9357	-3.1341	0.0017235	0.0053144	chr3
DGCR14	-1.81	-3.1338	0.0017256	0.0053144	chr16
DDX49	-1.74	-3.1337	0.0017261	0.0053144	chr8
CACTIN	-2.5246	-3.1336	0.0017267	0.0053144	chr19
EIF2B1	-1.7653	-3.1325	0.001733	0.0053254	chr5
MRPL11	-1.4116	-3.1319	0.0017367	0.0053254	chr19
RPL7	-1.5251	-3.1316	0.0017385	0.0053254	chr1
RPS18	-1.766	-3.1311	0.0017413	0.0053254	chr17
RRP9	-0.67117	-3.1297	0.0017496	0.0053334	chr9
SMG8	-2.0937	-3.1296	0.0017506	0.0053334	chr17
EDC3	-1.8583	-3.1291	0.0017534	0.0053334	chr9
ENDOG	-1.9157	-3.1289	0.0017549	0.0053334	chr2
CWC25	-2.0936	-3.1269	0.0017668	0.0053389	chr11
GRPEL2	-1.5462	-3.1266	0.0017684	0.0053389	chr18
RPS29	-0.98977	-3.1263	0.0017705	0.0053389	chr12
RPL26	-0.98977	-3.1263	0.0017705	0.0053389	chr11
RPL32	-0.98977	-3.1263	0.0017705	0.0053389	chr3
G3BP1	-1.4576	-3.123	0.0017905	0.0053909	chr11
ZNF768	-2.0478	-3.121	0.0018022	0.0054142	chr16
CNOT6L	-1.6748	-3.1208	0.0018038	0.0054142	chr5
RANGRF	-2.1059	-3.1198	0.0018095	0.0054229	chr11
PUM1	-0.62271	-3.1156	0.001836	0.0054938	chr4
SNRNP48	-1.4797	-3.1149	0.0018402	0.0054978	chr13
RBMY1B	-2.4914	-3.1137	0.0018478	0.005512	chrY
HNRNPDL	-1.6768	-3.1122	0.0018567	0.0055303	chr4
KHDRBS3	-1.5452	-3.1108	0.0018655	0.0055461	chr15
RARS2	-1.8147	-3.1105	0.0018678	0.0055461	chr4
DDX47	-1.7634	-3.1072	0.0018886	0.005588	chr14
RPL7L1	-1.3315	-3.1063	0.0018944	0.005588	chr17
SDAD1	-1.0226	-3.1056	0.0018992	0.005588	chr4
NXT1	-1.0226	-3.1056	0.0018992	0.005588	chr2
DBR1	-0.51787	-3.1056	0.0018991	0.005588	chr9
GAR1	-0.51787	-3.1056	0.0018991	0.005588	chr3
MRPL12	-1.5012	-3.1046	0.0019051	0.0055968	chr11
PABPC3	-1.9602	-3.1039	0.0019096	0.0056017	chr13
TRA2B	-1.5445	-3.1033	0.0019137	0.0056053	chr16
DYNC1H1	-1.8879	-3.1023	0.0019201	0.0056155	chr12
RPS5	-1.8469	-3.1013	0.001927	0.0056272	chr7
RPL37A	-2.0402	-3.1002	0.0019337	0.0056381	chr1
HNRNPH2	-1.6403	-3.0989	0.0019424	0.0056552	chrX
PSMD11	-1.6159	-3.0979	0.0019488	0.0056622	chr17
DNMT3B	-2.3586	-3.0976	0.0019509	0.0056622	chr2

RPS15	-1.6043	-3.0972	0.0019536	0.0056622	chr10
PABPC1L2A	-1.1742	-3.0963	0.0019596	0.0056672	chrX
LSM11	-2.0916	-3.0955	0.0019646	0.0056672	chr11
MRPL20	-2.1416	-3.095	0.0019683	0.0056672	chr4
DDX10	-0.88433	-3.0945	0.0019712	0.0056672	chr9
PIH1D3	-2.0373	-3.0939	0.0019752	0.0056672	chr4
SCAF4	-1.8779	-3.0933	0.0019794	0.0056672	chr16
FBL	-1.5437	-3.0924	0.0019853	0.0056672	chr7
RBM39	-0.62193	-3.0912	0.0019933	0.0056672	chr2
EIF3G	-0.62193	-3.0912	0.0019933	0.0056672	chr9
EXOSC4	-0.62193	-3.0912	0.0019933	0.0056672	chr15
EIF3C	-0.62193	-3.0912	0.0019933	0.0056672	chr7
RPS6	-0.62193	-3.0912	0.0019933	0.0056672	chr4
VCP	-0.62193	-3.0912	0.0019933	0.0056672	chr4
AGO3	-0.84575	-3.0901	0.0020012	0.0056812	chr1
CLK3	-2.4345	-3.0877	0.0020172	0.00571	chr9
EIF3F	-2.2104	-3.0868	0.0020233	0.0057188	chr7
PARP4	-1.7792	-3.0847	0.0020377	0.0057212	chr14
RPL12	-2.1285	-3.0843	0.0020403	0.0057212	chr2
LARP6	-2.0654	-3.0839	0.0020429	0.0057212	chr15
DDX58	-1.4492	-3.0839	0.0020432	0.0057212	chr4
DDX19B	-2.09	-3.083	0.0020493	0.0057212	chr8
COPB1	-1.0542	-3.0828	0.0020507	0.0057212	chr7
EIF4G3	-1.0542	-3.0828	0.0020507	0.0057212	chr4
DHX8	-1.0542	-3.0828	0.0020507	0.0057212	chr11
RPL37	-1.0542	-3.0828	0.0020507	0.0057212	chr15
RBM4	-2.5045	-3.0813	0.0020608	0.0057412	chr19
RPL24	-0.51654	-3.0794	0.0020742	0.0057624	chr3
YTHDF2	-2.078	-3.079	0.0020767	0.0057624	chr4
HNRNPCL1	-2.0646	-3.079	0.0020773	0.0057624	chr1
SLBP	-2.0652	-3.0779	0.0020848	0.0057749	chr5
EIF3B	-1.2015	-3.0758	0.0020991	0.0058063	chr5
CTIF	-1.8445	-3.0718	0.0021281	0.0058779	chr18
HNRNPA0	-2.6905	-3.0708	0.0021348	0.0058882	chr13
RRP7A	-2.1276	-3.0693	0.0021457	0.0059099	chr15
OASL	-1.9863	-3.0686	0.0021508	0.0059155	chr12
AIMP1	-2.3766	-3.0675	0.0021589	0.0059293	chr3
UPF1	-1.5838	-3.0658	0.0021709	0.005954	chr8
MBNL2	-1.3046	-3.0645	0.0021802	0.0059708	chr14
SMG9	-2.3168	-3.0628	0.0021931	0.0059918	chr12
METTL2B	-1.5626	-3.0626	0.002194	0.0059918	chr7
METTL5	-1.6026	-3.0576	0.0022314	0.0060852	chr2
MRPS18C	-2.1107	-3.0563	0.0022405	0.0060929	chr5
CNOT6	-1.9067	-3.0559	0.0022435	0.0060929	chr11
RNASE8	-2.7611	-3.0558	0.0022443	0.0060929	chr14
CARS	-2.1267	-3.0555	0.0022467	0.0060929	chr7
SHFM1	-2.6782	-3.0548	0.0022523	0.0060994	chr6
INTS5	-1.7777	-3.0537	0.0022606	0.0061134	chr19
FTSJ3	-1.5559	-3.0521	0.0022726	0.0061373	chr11
SRSF11	-1.5191	-3.0507	0.0022834	0.0061578	chr3
CPEB2	-2.8621	-3.05	0.0022886	0.0061611	chr4

TNPO3	-1.9062	-3.0497	0.002291	0.0061611	chr6
ENDOU	-1.672	-3.0491	0.0022952	0.0061639	chr15
MRPL49	-2.037	-3.0472	0.0023095	0.0061939	chr19
RP9	-2.3845	-3.0442	0.0023326	0.0062472	chr9
TAF15	-2.1864	-3.0433	0.0023401	0.0062494	chr11
EEF1E1	-1.9054	-3.0422	0.0023485	0.0062494	chr10
MYEF2	-2.2907	-3.0421	0.0023492	0.0062494	chr2
COPZ1	-1.8904	-3.042	0.0023499	0.0062494	chr15
MECP2	-1.6025	-3.0418	0.0023515	0.0062494	chrX
EIF5A	-2.2074	-3.0417	0.0023528	0.0062494	chr11
RRNAD1	-2.5582	-3.0412	0.0023563	0.0062502	chr3
LSM12	-2.5859	-3.04	0.0023656	0.0062662	chr11
GLE1	-0.56983	-3.0372	0.0023882	0.0063004	chr2
SF3B1	-0.56983	-3.0372	0.0023882	0.0063004	chr1
EIF2S1	-0.56983	-3.0372	0.0023882	0.0063004	chr12
RPUSD2	-2.5314	-3.0326	0.0024242	0.0063571	chr2
PAPOLB	-1.1141	-3.0326	0.0024246	0.0063571	chr5
IMP4	-1.1141	-3.0326	0.0024246	0.0063571	chr1
ACIN1	-1.1141	-3.0326	0.0024246	0.0063571	chr14
SMG6	-1.6306	-3.0324	0.0024261	0.0063571	chr11
ANKHD1	-1.4958	-3.0308	0.0024388	0.0063817	chr18
PABPN1	-1.9049	-3.0301	0.0024447	0.0063885	chr14
RPL8	-2.4574	-3.0288	0.0024553	0.0064077	chr15
PRPF8	-1.8895	-3.0284	0.0024589	0.0064083	chr11
RBMX2	-1.6252	-3.0234	0.0024992	0.0065046	chrX
RPS27	-0.51812	-3.0222	0.0025091	0.0065217	chr3
EXOSC7	-1.9342	-3.0171	0.0025518	0.0066063	chr9
DDX39B	-1.934	-3.0169	0.0025541	0.0066063	chr17
NANOS3	-2.4117	-3.0158	0.0025628	0.0066063	chr8
AKAP1	-1.7573	-3.0158	0.0025634	0.0066063	chr11
PURA	-1.7698	-3.0155	0.0025655	0.0066063	chr18
SNRPD3	-1.8403	-3.0106	0.0026075	0.0067054	chr10
AARS2	-2.4703	-3.0071	0.0026377	0.0067741	chr17
SNRNP40	-1.5368	-3.0059	0.002648	0.0067917	chr4
NSUN6	-1.4449	-3.0047	0.0026586	0.0068009	chr10
KRAS	-1.4936	-3.0012	0.0026887	0.0068689	chr6
HNRNPA1	-1.7085	-2.9995	0.0027044	0.0068997	chr12
NARS	-2.0617	-2.9952	0.0027429	0.0069888	chr18
DHX58	-2.2745	-2.9939	0.0027546	0.0069941	chr11
RRP1	-2.7106	-2.9934	0.0027588	0.0069941	chr10
PNO1	-1.0864	-2.9933	0.0027594	0.0069941	chr14
TDRD10	-2.4292	-2.9918	0.0027733	0.00702	chr1
MRRF	-1.7007	-2.9909	0.0027815	0.0070317	chr2
TDRD1	-2.2059	-2.9892	0.0027974	0.0070628	chr19
UTP11L	-0.45743	-2.9877	0.0028108	0.00708	chr4
INTS7	-1.4009	-2.9876	0.0028116	0.00708	chr1
TDRD7	-1.2715	-2.9864	0.0028233	0.0071003	chr4
INTS2	-2.3122	-2.9857	0.002829	0.0071055	chr11
RPL17	-1.8858	-2.9827	0.0028576	0.007168	chr18
STAU1	-2.1708	-2.9795	0.0028869	0.0072321	chr2
RPS12	-1.1703	-2.9782	0.0028993	0.0072517	chr10

RBM46	-1.9609	-2.9779	0.0029022	0.0072517	chr3
NOP10	-2.2528	-2.9763	0.0029173	0.0072801	chr15
UPF3B	-1.376	-2.9749	0.0029307	0.0073042	chrX
ZCCHC2	-2.3328	-2.9728	0.0029513	0.0073412	chr1
MRPL54	-1.8039	-2.9721	0.0029576	0.0073412	chr10
GTPBP3	-1.9438	-2.9711	0.0029669	0.0073471	chr8
EIF1AY	-2.1206	-2.9695	0.0029825	0.0073764	chrY
DDX18	-2.1698	-2.9688	0.0029897	0.0073849	chr1
SRRM1	-0.51455	-2.9642	0.0030346	0.0074766	chr4
RRP36	-0.51455	-2.9642	0.0030346	0.0074766	chr17
EIF2A	-1.1713	-2.9628	0.0030481	0.0075003	chr3
MRPS10	-2.1459	-2.9607	0.0030689	0.0075169	chr17
FTSJ1	-2.2033	-2.9603	0.0030733	0.0075169	chrX
CPSF6	-2.0846	-2.9602	0.003074	0.0075169	chr10
PRPF18	-1.8696	-2.9561	0.0031156	0.0076014	chr2
THOC7	-1.9739	-2.956	0.0031166	0.0076014	chr14
RRP8	-1.8823	-2.9533	0.0031439	0.0076584	chr7
APOBEC1	-2.1809	-2.9511	0.0031662	0.007703	chr6
SNRPG	-1.197	-2.95	0.0031775	0.0077116	chr6
MRPS26	-1.9732	-2.9497	0.0031808	0.0077116	chr2
CCT3	-1.0919	-2.9496	0.0031817	0.0077116	chr1
SNRPE	-1.9635	-2.9471	0.0032077	0.007738	chr1
LUC7L2	-1.9222	-2.947	0.0032085	0.007738	chr6
THOC2	-2.0493	-2.9447	0.0032332	0.0077878	chrX
METTL3	-2.4356	-2.9414	0.0032674	0.0078604	chr14
MRPL38	-2.1917	-2.9403	0.0032787	0.0078778	chr11
CCT8	-1.3624	-2.9395	0.0032875	0.0078863	chr16
HNRNPA2B1	-2.058	-2.9392	0.0032904	0.0078863	chr6
RBM14	-2.1806	-2.9386	0.0032972	0.007893	chr19
SLU7	-1.4642	-2.9372	0.0033115	0.0079172	chr11
DROSHA	-1.8259	-2.9369	0.0033155	0.0079172	chr15
MRPS2	-1.9431	-2.9357	0.0033282	0.0079377	chr2
MRPS16	-1.7184	-2.9347	0.0033384	0.0079522	chr14
CELF3	-2.4169	-2.9342	0.003344	0.0079558	chr3
MEX3B	-2.167	-2.9306	0.0033834	0.0080397	chr7
RBFOX1	-1.9429	-2.9293	0.0033976	0.0080635	chr16
CCDC59	-2.4067	-2.9285	0.0034061	0.008074	chr10
VARS2	-2.1501	-2.9268	0.0034252	0.0081091	chr17
POP5	-1.6697	-2.9258	0.003436	0.0081248	chr5
MRPS18A	-2.3779	-2.9248	0.0034466	0.0081331	chr17
RNASE4	-1.6712	-2.923	0.0034668	0.008158	chr14
FUBP1	-1.223	-2.9214	0.0034845	0.0081897	chr3
FARS2	-1.3238	-2.9153	0.0035534	0.008334	chr6
TNPO2	-2.2286	-2.9152	0.0035545	0.008334	chr8
RRS1	-1.6892	-2.9119	0.0035928	0.0083933	chr1
LSM7	-1.6892	-2.9119	0.0035928	0.0083933	chr19
OAS2	-1.961	-2.9115	0.0035974	0.008394	chr5
RPS14	-1.8006	-2.9104	0.0036093	0.0084076	chr5
MRPL55	-1.9705	-2.9102	0.0036119	0.0084076	chr11
UPF3A	-2.13	-2.9092	0.0036233	0.008424	chr8
RBM15B	-1.956	-2.9078	0.0036394	0.0084513	chr9

MRPS18B	-1.7999	-2.9073	0.0036453	0.0084532	chr17
C9orf114	-1.8502	-2.907	0.003649	0.0084532	chr6
POLDIP3	-1.8664	-2.9022	0.0037058	0.0085746	chr15
MRPL1	-1.5908	-2.8983	0.0037522	0.0086614	chr5
TARDBP	-0.39497	-2.8968	0.0037699	0.0086918	chr4
APOBEC3B	-1.8956	-2.8947	0.0037955	0.0087381	chr22
HNRNPAB	-2.1773	-2.8944	0.003799	0.0087381	chr11
NIP7	-0.51486	-2.8929	0.0038166	0.0087598	chr8
MVP	-2.3539	-2.8925	0.003822	0.0087598	chr7
CPSF3	-1.2482	-2.8925	0.0038218	0.0087598	chr12
ZCCHC17	-2.2781	-2.8915	0.0038345	0.0087741	chr4
EIF4G2	-1.5814	-2.8912	0.0038373	0.0087741	chr7
UTP3	-2.2602	-2.89	0.0038519	0.0087939	chr5
SNRPC	-2.2341	-2.8898	0.003855	0.0087939	chr17
SNRNP70	-1.9685	-2.887	0.0038893	0.0088343	chr7
RBM33	-2.6576	-2.886	0.003902	0.0088387	chr5
EEF2K	-1.6678	-2.8824	0.0039466	0.0089293	chr7
MRPL41	-1.5604	-2.8797	0.003981	0.0089861	chr2
RBM4B	-1.5049	-2.8766	0.0040203	0.0090644	chr19
EXOG	-1.648	-2.8753	0.004037	0.0090914	chr9
XPO5	-2.1767	-2.8748	0.004043	0.0090945	chr17
NANOS1	-2.211	-2.8709	0.0040926	0.0091847	chr19
DAZ3	-2.6027	-2.8687	0.0041221	0.0092258	chrY
DHX9	-0.4557	-2.8684	0.0041252	0.0092258	chr1
NXF1	-0.4557	-2.8684	0.0041252	0.0092258	chr19
RPL11	-1.953	-2.8671	0.0041428	0.0092439	chr4
RBM27	-2.1598	-2.8671	0.0041428	0.0092439	chr18
ADAT3	-2.7858	-2.8666	0.0041493	0.0092477	chr10
CDK11B	-2.2687	-2.8652	0.0041681	0.009279	chr4
AGO1	-2.5528	-2.8634	0.0041918	0.0093211	chr1
HNRNPA3	-2.4412	-2.8611	0.0042215	0.0093763	chr2
ERI3	-2.0231	-2.8603	0.0042322	0.0093892	chr4
ATXN2	-2.3713	-2.8599	0.004238	0.0093914	chr5
MRPL42	-1.9952	-2.8557	0.0042942	0.0094942	chr10
NCL	-1.9952	-2.8557	0.0042942	0.0094942	chr1
OAS1	-2.1391	-2.8551	0.0043021	0.0095008	chr12
TUBGCP3	-1.9856	-2.8527	0.0043352	0.009563	chr8
RBMXL1	-2.0223	-2.8506	0.0043636	0.0096148	chr8
PLD6	-2.0521	-2.8491	0.0043847	0.0096504	chr17
RPL36A	-2.1975	-2.8481	0.0043977	0.0096632	chrX
CPSF7	-2.2124	-2.8474	0.0044085	0.0096632	chr19
TDRD5	-2.1974	-2.8467	0.0044177	0.0096632	chr1
FASTK	-2.034	-2.8465	0.0044205	0.0096632	chr5
LSM3	-2.2505	-2.8406	0.0045027	0.0098198	chr6
HTATSF1	-1.947	-2.8401	0.0045101	0.0098198	chrX
SRSF4	-2.3775	-2.84	0.0045115	0.0098198	chr4
FAM120B	-2.1257	-2.8399	0.0045124	0.0098198	chr17
MRPL51	-2.0324	-2.8383	0.0045355	0.009859	chr6
UBAP2L	-2.3841	-2.8372	0.0045512	0.0098822	chr3
NOL8	-1.2965	-2.8343	0.0045926	0.0099609	chr13
LUC7L3	-0.57585	-2.8333	0.0046071	0.0099777	chr11

SLTM	-1.8748	-2.8329	0.004613	0.0099777	chr9
ELAVL4	-1.6083	-2.8318	0.0046294	0.0099959	chr4
FUBP3	-2.448	-2.8298	0.0046578	0.010035	chr2
UPF2	-2.4203	-2.8298	0.0046571	0.010035	chr2
NUTF2	-2.0341	-2.8291	0.0046685	0.010047	chr16
MRPS27	-2.4209	-2.8284	0.0046784	0.010057	chr13
PARP12	-2.1841	-2.8254	0.0047227	0.010141	chr6
ZCCHC3	-2.5013	-2.8238	0.0047455	0.010179	chr2
MRPL18	-2.1475	-2.8216	0.0047785	0.010238	chr17
LSM4	-2.2276	-2.819	0.0048172	0.010278	chr8
HNRNPC	-2.1471	-2.8158	0.0048657	0.010356	chr14
ZCCHC14	-2.2909	-2.8142	0.00489	0.010396	chr8
DCPS	-2.3609	-2.8133	0.0049042	0.010396	chr9
LSM14A	-1.9638	-2.8133	0.0049035	0.010396	chr7
TRDMT1	-1.5833	-2.8128	0.0049112	0.010396	chr2
DDX60L	-1.5833	-2.8128	0.0049112	0.010396	chr4
CHMP2A	-1.7772	-2.8114	0.0049325	0.01043	chr7
APOBEC3C	-2.019	-2.8107	0.004943	0.010441	chr22
RPS4Y1	-2.4939	-2.8083	0.0049805	0.010508	chrY
PIWIL2	-2.0185	-2.8035	0.0050551	0.010606	chr14
SMG1	-2.2832	-2.8032	0.0050595	0.010606	chr7
EIF4A3	-1.4554	-2.8009	0.0050958	0.01067	chr11
SUGP1	-2.3382	-2.795	0.0051907	0.010857	chr8
THOC3	-1.947	-2.7939	0.0052074	0.010881	chr13
RPL10	-2.2167	-2.7922	0.0052348	0.010926	chrX
UTP14C	-1.4978	-2.7901	0.0052688	0.010949	chr13
SNRPN	-2.0582	-2.7889	0.0052888	0.010956	chr7
DQX1	-2.1595	-2.7871	0.0053187	0.011006	chr6
DDX3X	-2.5328	-2.7858	0.0053401	0.011027	chrX
DND1	-2.0109	-2.7858	0.0053392	0.011027	chr18
DDX6	-2.0574	-2.7805	0.0054274	0.011194	chr9
EIF3L	-1.9605	-2.7802	0.0054326	0.011194	chr15
SRRT	-2.2025	-2.7793	0.0054469	0.011212	chr5
RNF113A	-2.3939	-2.7787	0.0054582	0.011223	chrX
MEX3C	-2.527	-2.7779	0.0054708	0.011237	chr18
FAM98A	-1.8876	-2.7775	0.0054773	0.011239	chr17
RPL36	-2.323	-2.7772	0.0054836	0.01124	chr17
ZFR	-2.0163	-2.7763	0.0054983	0.011256	chr15
RPSA	-1.6585	-2.776	0.0055032	0.011256	chr9
CELF2	-2.6699	-2.7712	0.0055848	0.011375	chr2
DDX39A	-2.7151	-2.7663	0.0056689	0.011534	chr19
MARS2	-2.4721	-2.7656	0.0056814	0.011547	chr1
METTL2A	-0.32681	-2.7645	0.0057009	0.011575	chr17
NUDT16	-2.1236	-2.7636	0.0057161	0.011594	chr9
DAZL	-2.428	-2.7621	0.005743	0.011636	chr17
YBX1	-2.2801	-2.7602	0.0057762	0.011691	chr4
HNRNPD	-2.2357	-2.7593	0.0057924	0.011696	chr5
MRPL23	-2.0952	-2.7579	0.0058169	0.011724	chr7
RPL10L	-2.393	-2.756	0.0058519	0.011783	chr12
ESRP1	-2.2253	-2.7546	0.0058761	0.011816	chr4
SRSF9	-2.2798	-2.7544	0.0058805	0.011816	chr5

PUSL1	-2.4266	-2.7537	0.0058929	0.011829	chr4
TDRD3	-1.8276	-2.7529	0.0059078	0.011846	chr14
LSM10	-2.0073	-2.7508	0.0059444	0.011907	chr4
FAM120C	-2.5395	-2.7494	0.0059707	0.011948	chrX
PSMB3	-1.3643	-2.7473	0.0060093	0.012012	chr11
USB1	-2.1693	-2.7466	0.0060221	0.012026	chr16
RPS3	-0.3928	-2.7451	0.0060495	0.012031	chr7
PRPF38B	-0.3928	-2.7451	0.0060495	0.012031	chr3
YARS2	-0.3928	-2.7451	0.0060495	0.012031	chr12
EIF2B5	-0.3928	-2.7451	0.0060495	0.012031	chr16
MTIF2	-0.32643	-2.7431	0.0060865	0.012055	chr11
PUS7	-0.32643	-2.7431	0.0060865	0.012055	chr5
SNUPN	-0.32643	-2.7431	0.0060865	0.012055	chr9
MRPS24	-2.2128	-2.7427	0.0060938	0.012057	chr11
MRPS15	-2.3413	-2.7416	0.0061142	0.012085	chr4
PCBP3	-2.168	-2.7383	0.0061766	0.012185	chr10
RPS3A	-1.8293	-2.7382	0.0061771	0.012185	chr3
AARSD1	-1.7501	-2.7355	0.0062283	0.012273	chr11
APOBEC4	-1.7848	-2.7302	0.0063301	0.012394	chr1
CPEB1	-2.1054	-2.7294	0.0063448	0.012394	chr7
MRPS30	-2.2568	-2.7284	0.0063645	0.012394	chr13
DHX37	-1.8531	-2.728	0.0063727	0.012394	chr5
SART1	-2.1055	-2.726	0.0064103	0.012394	chr19
SRSF6	-2.593	-2.7259	0.0064131	0.012394	chr2
RPUSD3	-1.9099	-2.7241	0.0064473	0.012394	chr6
RBM25	-2.0917	-2.7195	0.0065383	0.012394	chr12
KIAA0430	-2.7525	-2.7189	0.0065494	0.012394	chr16
GPATCH4	-1.5946	-2.7189	0.0065496	0.012394	chr3
MRPL14	-1.3858	-2.7187	0.0065545	0.012394	chr17
PRPF6	-1.3858	-2.7187	0.0065545	0.012394	chr2
MRPS12	-2.1563	-2.7184	0.00656	0.012394	chr7
MEX3A	-1.9977	-2.7175	0.0065784	0.012394	chr1
SMG7	-1.9553	-2.7153	0.0066216	0.012453	chr1
RBM12B	-2.4416	-2.7137	0.0066537	0.012487	chr8
AGO4	-2.4651	-2.7132	0.0066637	0.012487	chr1
MRPL57	-2.3188	-2.7122	0.0066848	0.012487	chr13
YBX3	-2.7557	-2.7121	0.0066849	0.012487	chr12
TBRG4	-2.0651	-2.7113	0.0067017	0.012487	chr11
MRPL19	-1.8383	-2.7073	0.0067827	0.012609	chr6
CNOT8	-1.7091	-2.7069	0.0067908	0.012612	chr11
SF3A2	-2.0896	-2.7027	0.0068783	0.012763	chr10
NSUN4	-2.1301	-2.6991	0.0069528	0.012889	chr4
FIP1L1	-1.8283	-2.6986	0.0069633	0.012896	chr5
RNASEH2C	-1.8077	-2.6963	0.0070106	0.012971	chr19
RPS21	-1.4067	-2.6902	0.0071402	0.013148	chr2
USP39	-1.983	-2.6878	0.0071934	0.013233	chr6
RRBP1	-2.4761	-2.6843	0.0072671	0.013351	chr2
SPEN	-1.9666	-2.6842	0.0072708	0.013351	chr4
RNASEH2B	-1.7687	-2.6799	0.0073635	0.013508	chr14
MRPL28	-2.4059	-2.6787	0.0073913	0.013546	chr17
RDM1	-0.45791	-2.6767	0.0074341	0.013548	chr11

EXOSC9	-2.541	-2.6749	0.0074752	0.01361	chr3
ATXN2L	-1.9386	-2.6724	0.0075316	0.0137	chr7
FTSJ2	-2.2518	-2.6669	0.0076565	0.013914	chr5
EIF3D	-1.4017	-2.6622	0.007763	0.014084	chr15
EMG1	-1.6637	-2.6621	0.0077649	0.014084	chr6
DHX38	-1.8722	-2.6465	0.008133	0.014725	chr8
SNRPD2	-2.2636	-2.6459	0.0081485	0.014739	chr7
CLP1	-1.9676	-2.6451	0.0081671	0.014758	chr2
PTBP2	-2.2211	-2.6448	0.0081746	0.014758	chr3
RPL22L1	-2.4136	-2.6435	0.0082044	0.014798	chr3
TSEN15	-2.3028	-2.6412	0.0082608	0.014841	chr1
PIWIL4	-2.1774	-2.637	0.0083634	0.015001	chr9
NXF3	-2.5289	-2.6294	0.0085525	0.015312	chrX
PHF5A	-1.7533	-2.6281	0.0085854	0.015357	chr15
RPA1	-1.871	-2.6209	0.0087686	0.015599	chr11
NXF2	-2.3891	-2.6163	0.0088887	0.015798	chrX
SF3B3	-2.182	-2.6108	0.0090332	0.01604	chr8
PELP1	-2.4099	-2.608	0.0091081	0.016158	chr11
XRCC6	-1.4667	-2.6067	0.0091429	0.016194	chr15
GRSF1	-1.9652	-2.6063	0.0091517	0.016194	chr5
NUP98	-1.8354	-2.6063	0.0091535	0.016194	chr7
ELAC2	-1.4862	-2.6047	0.0091946	0.016218	chr11
PCBP2	-1.9795	-2.6008	0.0093016	0.016322	chr15
DHX35	-2.4914	-2.5941	0.0094835	0.016626	chr2
PPARGC1B	-2.4071	-2.5913	0.009561	0.016747	chr18
SNRNP200	-0.3253	-2.5886	0.0096356	0.016802	chr2
NAPA	-0.3253	-2.5886	0.0096356	0.016802	chr7
HNRNPUL2	-0.3253	-2.5886	0.0096356	0.016802	chr19
SREK1	-0.3253	-2.5886	0.0096356	0.016802	chr5
PPIL4	-0.3253	-2.5886	0.0096356	0.016802	chr6
MRPS36	-2.1668	-2.5789	0.0099118	0.017237	chr13
FUS	-2.4206	-2.5771	0.0099625	0.017309	chr7
MRPL2	-2.0039	-2.5757	0.010003	0.017364	chr17
PTBP3	-2.2137	-2.5696	0.010181	0.017626	chr9
MRPS33	-1.8472	-2.5646	0.01033	0.017867	chr6
PRPF38A	-1.8622	-2.5547	0.010627	0.018328	chr4
HNRNPU	-2.2486	-2.5545	0.010634	0.018328	chr1
PPRC1	-1.5046	-2.5526	0.010691	0.0184	chr19
SRSF2	-1.5231	-2.5525	0.010694	0.0184	chr11
NOVA2	-2.538	-2.5394	0.011105	0.019005	chr7
SNRPD1	-2.1137	-2.5364	0.011199	0.01915	chr18
RBMS1	-2.1983	-2.5335	0.011291	0.01929	chr2
FXR1	-2.4539	-2.5296	0.011419	0.019491	chr3
SAMHD1	-2.2506	-2.528	0.011471	0.019563	chr2
GEMIN8	-1.5229	-2.5262	0.011532	0.019646	chrX
CELF6	-2.4386	-2.5259	0.011541	0.019646	chr9
DDX28	-2.3905	-2.5171	0.011834	0.020057	chr8
MAGOH	-2.4393	-2.5164	0.011854	0.020075	chr4
PABPN1L	-1.7712	-2.5077	0.01215	0.020558	chr8
ZNF579	-2.4847	-2.503	0.012315	0.020818	chr19
GEMIN4	-2.4903	-2.5005	0.012402	0.020947	chr11

DDX50	-2.4349	-2.4975	0.012507	0.021015	chr10
TDRD9	-2.1231	-2.4899	0.012776	0.02143	chr12
POLR2I	-2.3627	-2.484	0.012992	0.021754	chr7
MTRF1L	-2.4707	-2.4821	0.01306	0.021848	chr10
DDX60	-1.7882	-2.4796	0.013154	0.021987	chr8
GEMIN7	-2.4141	-2.478	0.013211	0.022064	chr7
ELAVL1	-2.2498	-2.4722	0.013429	0.022312	chr8
HNRNPA1L2	-2.5268	-2.4694	0.013533	0.022467	chr13
IFIT1	-2.5241	-2.4685	0.013568	0.022505	chr19
RBM19	-2.2652	-2.4612	0.013848	0.02295	chr5
RBPMS2	-2.416	-2.4555	0.014068	0.023294	chr9
APOBEC3A	-2.1752	-2.4506	0.014263	0.023417	chr22
ZFR2	-2.1534	-2.4497	0.014297	0.023449	chr10
ANKRD17	-1.9576	-2.4495	0.014307	0.023449	chr5
EIF2S3L	-2.2869	-2.4411	0.014641	0.023977	chr12
KHDRBS1	-2.2872	-2.4404	0.01467	0.024004	chr4
RPS24	-2.3578	-2.4371	0.014807	0.024207	chr14
DCP1A	-1.9977	-2.4308	0.015066	0.02461	chr14
NOL7	-1.9695	-2.4268	0.015234	0.024739	chr13
NELFE	-2.1857	-2.4181	0.015603	0.025317	chr6
BFP	-3.1805	-2.4025	0.016284	0.026073	chr17
DHX16	-1.6091	-2.3996	0.016415	0.026262	chr17
ETF1	-2.4635	-2.3987	0.016454	0.026303	chr18
PSMB2	-0.25252	-2.3923	0.016745	0.026657	chr4
RPS13	-0.25252	-2.3923	0.016745	0.026657	chr7
RPL27A	-0.25252	-2.3923	0.016745	0.026657	chr7
XRN1	-0.25252	-2.3923	0.016745	0.026657	chr3
SRSF1	-0.25252	-2.3923	0.016745	0.026657	chr11
CPSF2	-2.1836	-2.3872	0.016978	0.027005	chr12
SRSF8	-2.5996	-2.3747	0.017563	0.027575	chr11
DHX29	-2.1387	-2.3731	0.017641	0.027675	chr13
NUDT16L1	-2.3047	-2.3719	0.017695	0.027736	chr16
INTS4	-2.0057	-2.3709	0.017747	0.027796	chr7
MSI2	-1.84	-2.3674	0.017915	0.028015	chr11
REPIN1	-2.2384	-2.3596	0.018294	0.028583	chr6
RBM12	-2.0813	-2.3543	0.018559	0.028974	chr2
APOBEC3D	-1.9737	-2.3523	0.018659	0.029107	chr22
RTCB	-2.0171	-2.3494	0.018803	0.029188	chr22
NXF2B	-2.4104	-2.3485	0.018847	0.029188	chrX
RBM34	-2.2374	-2.3386	0.019355	0.029951	chr8
RPS10	-1.6571	-2.3283	0.019897	0.030766	chr17
EIF2B4	-2.426	-2.3247	0.020088	0.031037	chr5
EEF2	-1.9558	-2.3172	0.020493	0.031412	chr10
ZC3HC1	-2.4764	-2.296	0.021675	0.033198	chr6
DAZ4	-2.325	-2.2796	0.022633	0.034392	chrY
RPL19	-2.2061	-2.2779	0.02273	0.034513	chr11
XPO4	-2.4101	-2.2684	0.023305	0.035359	chr14
GEMIN2	-2.2631	-2.2568	0.024018	0.036074	chr14
EXOSC10	-2.2509	-2.2424	0.024935	0.037422	chr4
MARS	-1.7172	-2.2386	0.02518	0.03776	chr10
MRPL45	-2.3332	-2.2362	0.02534	0.03797	chr11

MRPL4	-2.2894	-2.2351	0.02541	0.038047	chr9
SRSF12	-1.978	-2.2313	0.025662	0.038324	chr4
ACTR1A	-2.4039	-2.2298	0.025758	0.038324	chr19
FASTKD1	-2.1125	-2.2292	0.025802	0.038324	chr2
SARS2	-1.9575	-2.2264	0.025991	0.038353	chr7
NARS2	-1.7316	-2.2172	0.026612	0.03924	chr7
NXT2	-1.3341	-2.1659	0.030315	0.044263	chrX
SMG5	-2.4781	-2.1553	0.031142	0.04503	chr3
SRPR	-0.17363	-2.1464	0.031843	0.04594	chr9
U2SURP	-0.17363	-2.1464	0.031843	0.04594	chr3
SYNCRIP	-0.17363	-2.1464	0.031843	0.04594	chr6
CLTC	-0.19177	-2.1155	0.034385	0.048987	chr17
RPL35	-1.9871	-2.0545	0.039929	0.056267	chr2
RPL23A	-1.9727	-2.0502	0.040348	0.056816	chr11
MRPL27	-2.2558	-2.0155	0.043852	0.061129	chr11
THOC6	-1.8607	-1.9687	0.048988	0.067703	chr17
RPL4	-0.087587	-1.8242	0.068117	0.090527	chr15
EIF4A1	-0.087587	-1.8242	0.068117	0.090527	chr17
RPL34	-0.087587	-1.8242	0.068117	0.090527	chr4
RPL18A	-0.087587	-1.8242	0.068117	0.090527	chr19
RNASEK	-0.087587	-1.8242	0.068117	0.090527	chr17
NMD3	-0.087587	-1.8242	0.068117	0.090527	chr3
NACA	-0.087587	-1.8242	0.068117	0.090527	chr12
MRPL15	-0.087587	-1.8242	0.068117	0.090527	chr8
SF3B6	-0.85083	-1.6084	0.10774	0.13881	chr2
RPL15	-0.66083	-1.5457	0.12217	0.15719	chr21
MRPL36	-0.14442	-0.84543	0.39787	0.48765	chr13
RPL38	-0.3205	-0.78664	0.43149	0.52853	chr17
EIF4E	-8.88E-05	-5.72E-06	1	1	chr3
RNASEL	-4.01E-05	-4.41E-06	1	1	chr1
AAVS1	0	0	1	1	chr19
BMS1	4.3616e-05	0.022036	0.98242	0.99008	chr6
NIFK	1.0627	0.65072	0.51523	0.53096	chr2
OAS3	7.1338e-05	0.91016	0.36274	0.44544	chr5
SRSF10	1.6889	1.0841	0.2783	0.3457	chr4
RPS4Y2	8.2186e-05	1.2896	0.19718	0.24779	chrY
PHAX	9.3924e-05	1.4104	0.15841	0.20049	chr18
IPO9	0.25933	1.6172	0.10583	0.13644	chr1
RPL41	0.0068848	1.654	0.098128	0.12778	chr12
ZCCHC9	0.15557	1.7245	0.084609	0.11115	chr13
YY1	12.024	1.8705	0.061414	0.082811	chr12
ARCN1	0.0069232	2.5708	0.010148	0.017584	chr11
BRIX1	0.0069263	2.7172	0.0065838	0.012394	chr5
CLK1	0.0069263	2.7172	0.0065838	0.012394	chr2
CRNKL1	0.0069263	2.7172	0.0065838	0.012394	chr20
DDX1	0.0069263	2.7172	0.0065838	0.012394	chr2
DDX24	0.0069263	2.7172	0.0065838	0.012394	chr14
DDX46	0.0069263	2.7172	0.0065838	0.012394	chr5
DDX55	0.0069263	2.7172	0.0065838	0.012394	chr12
EEF1A1	0.0069263	2.7172	0.0065838	0.012394	chr6
EIF2B3	0.0069263	2.7172	0.0065838	0.012394	chr1

EIF2S3	0.0069263	2.7172	0.0065838	0.012394	chrX
FASTKD5	0.0069263	2.7172	0.0065838	0.012394	chr20
GTPBP10	0.0069263	2.7172	0.0065838	0.012394	chr7
IPO13	0.0069263	2.7172	0.0065838	0.012394	chr1
KPNB1	0.0069263	2.7172	0.0065838	0.012394	chr17
MRPL24	0.0069263	2.7172	0.0065838	0.012394	chr1
NUP153	0.0069263	2.7172	0.0065838	0.012394	chr6
PARS2	0.0069263	2.7172	0.0065838	0.012394	chr1
PRPF40A	0.0069263	2.7172	0.0065838	0.012394	chr2
RPL13A	0.0069263	2.7172	0.0065838	0.012394	chr19
RPL21	0.0069263	2.7172	0.0065838	0.012394	chr13
RPL29	0.0069263	2.7172	0.0065838	0.012394	chr3
RPL39	0.0069263	2.7172	0.0065838	0.012394	chrX
RPS8	0.0069263	2.7172	0.0065838	0.012394	chr1
THOC5	0.0069263	2.7172	0.0065838	0.012394	chr22
TIAL1	0.0069263	2.7172	0.0065838	0.012394	chr10
TSEN54	0.0069263	2.7172	0.0065838	0.012394	chr17
ZCRB1	0.0069263	2.7172	0.0065838	0.012394	chr12
ZNF106	0.0069263	2.7172	0.0065838	0.012394	chr15
MRPS23	0.00038422	17.278	6.8991e-67	6.6886e-64	chr11
MEX3D	0.0020049	19.498	1.1476e-84	2.2252e-81	chr10
MRPS7	0.00012696	1.2141e-05	0.99999	1	chr11
NSRP1	9.0474e-06	5.8704e-07	1	1	chr17
PSPC1	2.5165e-05	7.3399e-05	0.99994	1	chr13
